# One class classification as a practical approach for accelerating π–π co-crystal discovery†

**DOI:** 10.1039/d0sc04263c

**Published:** 2020-12-08

**Authors:** Aikaterini Vriza, Angelos B. Canaj, Rebecca Vismara, Laurence J. Kershaw Cook, Troy D. Manning, Michael W. Gaultois, Peter A. Wood, Vitaliy Kurlin, Neil Berry, Matthew S. Dyer, Matthew J. Rosseinsky

**Affiliations:** Department of Chemistry and Materials Innovation Factory, University of Liverpool 51 Oxford Street Liverpool L7 3NY UK M.S.Dyer@liverpool.ac.uk; Leverhulme Research Centre for Functional Materials Design, University of Liverpool Oxford Street Liverpool L7 3NY UK; Cambridge Crystallographic Data Centre 12 Union Road Cambridge CB2 1EZ UK; Materials Innovation Factory, Computer Science Department, University of Liverpool Liverpool L69 3BX UK

## Abstract

The implementation of machine learning models has brought major changes in the decision-making process for materials design. One matter of concern for the data-driven approaches is the lack of negative data from unsuccessful synthetic attempts, which might generate inherently imbalanced datasets. We propose the application of the one-class classification methodology as an effective tool for tackling these limitations on the materials design problems. This is a concept of learning based only on a well-defined class without counter examples. An extensive study on the different one-class classification algorithms is performed until the most appropriate workflow is identified for guiding the discovery of emerging materials belonging to a relatively small class, that being the weakly bound polyaromatic hydrocarbon co-crystals. The two-step approach presented in this study first trains the model using all the known molecular combinations that form this class of co-crystals extracted from the Cambridge Structural Database (1722 molecular combinations), followed by scoring possible yet unknown pairs from the ZINC15 database (21 736 possible molecular combinations). Focusing on the highest-ranking pairs predicted to have higher probability of forming co-crystals, materials discovery can be accelerated by reducing the vast molecular space and directing the synthetic efforts of chemists. Further on, using interpretability techniques a more detailed understanding of the molecular properties causing co-crystallization is sought after. The applicability of the current methodology is demonstrated with the discovery of two novel co-crystals, namely pyrene-6*H*-benzo[*c*]chromen-6-one (1) and pyrene-9,10-dicyanoanthracene (2).

## Abstract 2

Machine learning using one class classification on a database of existing co-crystals enables the identification of co-formers which are likely to form stable co-crystals, resulting in the synthesis of two co-crystals of polyaromatic hydrocarbons.

## Graphical abstract

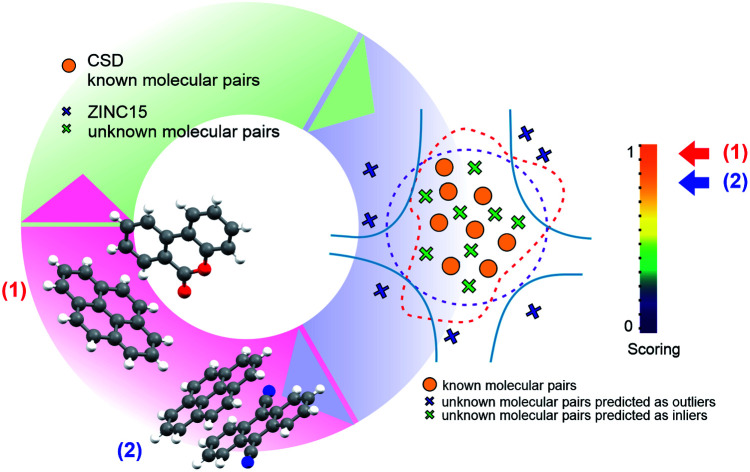

## Introduction

Machine learning approaches are increasingly incorporated into design workflows to explore and better understand the materials space.^1–3^ The ultimate goal is to identify more reliable methodologies and to develop smarter ways to accelerate the discovery of new materials with novel properties. Following the rapidly growing data availability, data driven approaches have taken hold as a tool for detecting patterns in known datasets and performing straightforward predictions. The quality of a machine learning model is highly dependent on the quality and the trends of the available data. Thus, the existence of reliable and complete databases is crucial for the development of predictive frameworks. However, machine learning models still suffer many limitations in terms of defining the appropriate representations of the target materials and/or achieving reliable predictions based solely on known instances or otherwise biased datasets. One-class classifiers are specifically designed to address this “positive examples only” problem that characterises many databases available in materials science (*e.g*., ICSD,^4^ CSD^5^). In the present work, we introduce one class classification as a promising methodology to tackle these drawbacks, using weakly-bound π–π organic co-crystals as a case study. The main goal is the identification of potential new candidates for co-crystallization among a wide range of polycyclic aromatic hydrocarbons (PAHs) and their subsequent synthesis and structural characterisation. Further an understanding about the connection between co-crystallization and chemical/structural properties of the molecules can be gained. As this is the first time one-class classification approaches are implemented in materials design, the existing algorithms are comprehensively investigated and critically discussed. The idea of applying one-class classification in materials science involves the accurate definition of the materials' class of interest, *e.g.*, the known PAHs co-crystals, such that any predictions can be made of novel co-crystals that might belong to the same class. As the interpretability of the machine learning models boosts their trustworthiness, we also investigated the contribution of the selected features on the final decisions for the co-crystal formation. Importantly, the applicability of the presented procedure is demonstrated by the identification of two novel co-formers and by the experimental realization of co-crystals 1 and 2, pyrene-6*H*-benzo[*c*]chromen-6-one and pyrene-9,10-dicyanoanthracene, respectively.

A co-crystal is a crystalline single-phase material composed of two or more different molecular compounds in a specific stoichiometry.^6–8^ These compounds are neither solvates/hydrates nor simple salts and are connected *via* one or more non-covalent interactions, such as hydrogen bonding, π–π stacking, halogen bonds and charge transfer (C-T) interactions.^9^ Co-crystal design has undoubtedly received most attention from the pharmaceutical industry. These compounds may offer the advantage of preserving the pharmacological properties of the Active Pharmaceutical Ingredient (API) whilst improving the physicochemical properties of the potential drug. Consequently, this attention stimulated the development of various theoretical and experimental studies for designing pharmaceutical co-crystals by selecting effective co-formers which are suitable with the API.^10^ Hydrogen bond propensity (HBP), p*K*_a_ rule, Fabian's method for molecular complementarity and Hansen solubility parameters are some of the most effective design approaches.^10^ The selection of the appropriate method is based mainly on the nature of the molecules and the way these molecules are interconnected.^6,11^

Co-crystals are gaining emerging interest in other cutting-edge research fields, ranging from photonic, to optical and electronic materials.^12–15^ It is well-known that most organic molecular crystals are insulators as there is no electronic interaction between the molecules.^16^ However, molecules with electron rich π-orbitals overcome this barrier, thus enabling electron mobility in cases where there is a favourable overlap of π-orbitals in adjacent molecules.^17^ π–π stacking is a common motif for obtaining electronic communication between the molecules and has been proven as an important characteristic of organic electronics (*e.g*., in conjugated polymers).^18,19^ A special category of molecules which self-assemble *via* π–π interactions are the PAHs, which can be regarded as two-dimensional graphite segments.^20^ Hence, PAHs are considered promising candidates for electronic materials and have been extensively used for designing co-crystals with desirable electron mobilities.^12,21,22^ Most of the research on electronic co-crystals is focused on the charge-transfer complexes between a good electron donor and a poor electron acceptor.^21,23,24^ This work suggests a promising pathway to expand the investigation on PAH-based co-crystals where the π–π interactions are the dominant structure-defining forces.

Although π–π interactions are desirable for designing electronic functional co-crystals, they are relatively weak compared to stronger interactions such as hydrogen or halogen bonding. In a recent computational work, Taylor *et al.* emphasized the difficulty in evaluating the thermodynamic stability of weakly-bound co-crystals without any additional group that can form charge transfer systems.^25^ The lack of strong energetic driving forces for co-crystallization makes the formation less favourable, thus these co-crystals are rare. In addition, the weak interactions give rise to shallow energy landscapes associated with multiple configurations of similar energy, hindering the structure prediction. The synthesis of weakly-bound co-crystal materials still remains a challenging task, albeit interaction between aromatic hydrocarbon systems have been suggested as a viable synthetic way on first principle calculations.^26^ To overcome the challenging limitations of predicting the π–π co-crystallization process, we will use data-driven approaches.

**Fig. 1:**
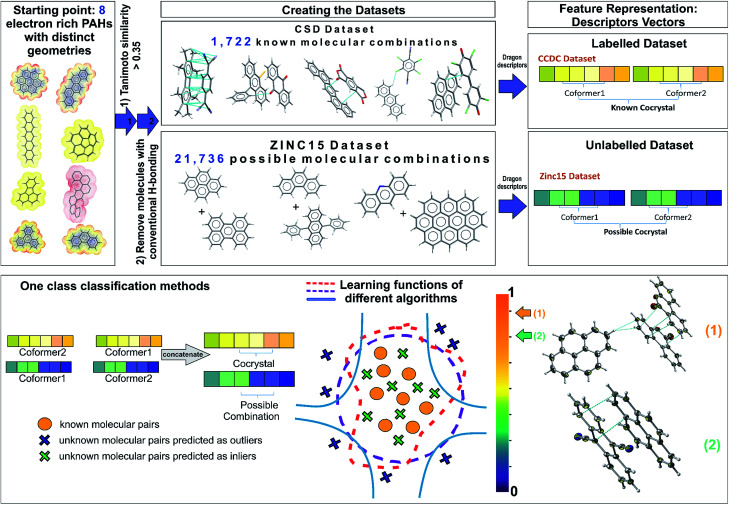
Schematic representation of the one class classification method for ranking possible hydrocarbon molecular pairs according to their probability to form co-crystals. Starting from a representative set of eight molecules that contain the molecular and electronic structure characteristics that reflect the likelihood of forming polyaromatic hydrocarbon (PAH) co-crystals, two different datasets were constructed. The labelled dataset involves all the existing PAH co-crystals in CSD, whereas the unlabelled dataset contains all the possible molecular combinations of PAHs from ZINC15 database. A molecular pair is represented as a concatenation of the molecular descriptors and is used as the input to various one class algorithms. Each of the implemented algorithms fits a different decision function to the labelled data and then scores the unlabelled combinations. The outcome of the models is a score (from 0 to 1) indicating the probability of two molecules forming a stable co-crystal. The known combinations as well as that part of the unlabelled data predicted as inliers have higher scores close to 1, where the points that could be regarded as anomalies (dark blue crosses) have scores below a selected threshold value. In the end, a ranked pool of combinations is produced, significantly reducing the initial dataset of interest. The best performing method is used for predicting the co-former combinations to be tested experimentally. The aforementioned workflow led to the discovery of two novel co-crystals 1 and 2.

The selection of the most appropriate machine learning approach is strongly dependent on the nature of the problem to be solved and the quality of the available data. The Cambridge Structural Database (CSD),^5^ which collects and curates publicly-available crystal structure data worldwide, including existing co-crystals, has been used as the information source for the current study. Each year multiple tens of thousands of new crystal structures are added to the database (53 199 new entries were added in 2019), increasing the demand for developing efficient and effective ways to extract non-trivial, valid and useful information to design new materials. The extracted dataset is composed of all the reported PAH co-crystals, with π–π stacking as the main interaction. After a careful investigation of the aforementioned dataset some observations are made. First of all, this category of co-crystals is a relatively small proportion, *i.e*., 12% of the complete set of co-crystals in the CSD, as a consequence it becomes more challenging to extract clear patterns that dominate these combinations. Secondly, there is a sparsity of negative experimental co-crystallization observations due to the lack of publications explicitly reporting the failure of molecular combinations to co-crystallize. Thus, a publication bias is created imposing an imbalance towards the target class due to the non-existence of negative data. Finally, there is an internal constitutional bias as most of the data are on a subset of heavily studied systems, rather than uniformly distributed over all possible systems, chemistries and structural families. Herein, we introduce a general approach to tackle the biased datasets. Our approach, as demonstrated in Fig. 1, is based on one class classification, a well-known method that has been applied to many research themes, such as novelty/outlier detection, concept learning or single class classification.^27^ However, it has not yet been employed in materials design problems. Contrary to other data-driven methods used for co-crystal design,^11,28^ one class classification does not require the generation of a large number of negative examples from unsuccessful experiments,^11^ and is able to involve the available molecular descriptors to derive chemical understanding of the predictions.^28^

As one class classification is imbalance tolerant, no specific distribution of the target class, PAH co-crystals, has to be assumed and thus one of the major problems in materials science regarding the lack of negative examples is tackled. The objective of one-class classification approaches is to accurately describe the ‘normality’, namely the distribution of the known dataset. It is assumed that the majority of the training dataset consists of ‘normal’ data.^29^ Thus, the one class classification algorithms learn to accurately describe the positive/known data. Deviations from this description are seen as anomalies and thus belong to a different class. The known data class is well characterized, and these instances are used as the training set. In this way the classifiers are focused on the deviations from the known distribution rather than focusing on the discrimination task between the classes. In this context, as labelled data we refer to all the positive combinations extracted from the CSD database (1722 molecular combinations, Fig. 1), whereas the unlabelled data are the pairs generated from the ZINC15 database (21 736 possible molecular combinations, Fig. 1). Each molecular pair is represented as a concatenation of molecular descriptors covering a wide range of properties. The presented predicted pairs refer to the molecules we have chosen based on their molecular similarity to the representative set of starting molecules (see Methods, Extracting the labelled dataset), however the list can be easily extended by including new molecular pairs in the training (labelled) dataset. The implemented algorithms for one-class classification (anomaly detection) are separated into eight traditional and one neural network and are discussed in ESI† (Section 2).

## Methods

### Extracting the labelled dataset

The labelled dataset of existing co-crystals in the CSD database was extracted using the CSD Python API (Application Programming Interface), version 2.0 (December 2018). As a starting point, eight molecules with extended polyaromatic systems are used as a representative set for searching the CSD and generating the co-crystal space of interest (>1700 molecular combinations). The selection of these representative eight initial molecules is performed on the basis of promising electronic properties (*e.g.*, known organic semiconductors) and distinct geometry (*i.e.*, the set is diverse in shape and symmetry). The names of the initial molecules as well as their 6 letter CSD Refcode were: coronene (CORONE), picene (ZZZYOC04), pentacene (PENCEN), triphenylene (TRIPHE), phenanthrene (PHENAN), fluoranthene (FLUANT), corannulene (CORANN01), dinaphthol-anthracene (DNAPAN). The similarity search function of the CSD Python API is applied to those molecules, using the standard CSD fingerprint similarity search with a Tanimoto similarity threshold of >0.35 (ref. ^30^) and accepting only neutral organic molecules with known SMILES identifiers. The 1722 entries in the resulting list are crystal structures that include either one of these molecules or molecules that are structurally similar to them (based on CSD molecular fingerprint similarity). The search aims to identify all the co-crystals that have as co-formers PAHs whilst the main interaction between them is π–π stacking. Each co-crystal in CSD can be represented as a combination of simplified molecular-input line-entry (SMILES)^31^ separated with a full stop *e.g.*, ‘c1cc2ccc3cccc4ccc(c1)c2c34.N#CC(C#N)=C1C=CC(C=C1)=C(C#N)C#N’ representing pyrene-TCNQ. Using this form, we can count the number of different molecules in the asymmetric unit and take into consideration the molecular stoichiometry of the co-formers. Combinations including common non-aromatic solvents are excluded. However, aromatic solvents are accepted *e.g.*, benzene, as the interactions in this case are only π–π stacking and these combinations might hold important information about the predictions this work is interested in. Finally, the molecular combinations are filtered using Pipeline Pilot (version 2017)^32^ by applying a SMARTS^33^ filter that removes molecules with acidic hydrogens, making sure that the main interaction among the co-crystals is π–π stacking. The whole process is schematically described in the ESI (Fig. S1†).

### Designing the unlabelled dataset

The dataset with the promising combinations of molecules is constructed using the ZINC15 database,^34^ which includes all the purchasable organic molecules. The molecules were taken from a version downloaded in August 2018. The same initial molecules used for the CSD search were used and the database was searched based on molecular Extended Connectivity Fingerprints (ECFP4) with a Tanimoto similarity threshold of >0.35.^35^ After filtering out the molecules with acidic hydrogens using Pipeline Pilot, the ZINC database reveals 209 molecules with calculated Dragon descriptors that match the selected similarity criteria with the initial molecules. All the possible combinations of these 209 molecules are taken into consideration, resulting in a dataset with 21 736 unique pairs.

### Dataset bias

Bias in natural science datasets,^36^ as well in CSD,^28^ has been reported before. Bias is a very general term and found in many categories. The studied dataset shows compositional bias due to the recurrence of some molecular components in the observed co-crystals. A different type of bias can be found considering the different molecular ratios, as the majority of the co-crystals of interest have 1 : 1 stoichiometry. In order to design co-crystals whose formation is driven by π–π stacking, the training set used was biased towards molecular combinations that are connected with that type of interaction. In some respect, we need this bias to build a target specific approach for detecting weakly interacting molecular pairs. However, our dataset is unbalanced as there are some popular co-crystal co-formers that tend to appear many times in π–π stacking pairs, *e.g.*, benzene, toluene, pyrene, which leads to these molecules being over-represented in the highest scored pairs. One objective of this study is thus to identify new co-crystal forming molecules that do not correspond to a previous database entry.

### Feature generation and engineering

In this context, features are defined as the molecular descriptors that uniquely represent each molecule. The chemical space of interest can be defined by the appropriate set of numerical descriptors that capture the characteristics and/or properties of the molecules. With *n* linearly independent descriptors, an *n*-dimensional space is defined. A careful selection of the appropriate descriptors is critical for the rational design and implementation of any machine learning method.^37^ Each molecule is represented as an *n*-dimensional vector with *n* being the number of the available descriptors calculated with Dragon software,^38^ version 6.0/2012. Traditional one-class classification approaches require extensive feature engineering as it is desirable to reduce the dimensions of the problem before the analysis. The dimensionality reduction is performed following the standard good practices for removing descriptors that are highly correlated to each other or describe similar properties.^39^ Features that are correlated more than 0.92 as well as those that have variance lower than 0.4 were removed from each co-former's dataset. The feature selection process was performed according to the molecular complementarity approach.^40^ All the pairwise correlations between the molecular pairs were calculated, after removing co-crystals containing benzene-like solvents to avoid possible bias on the feature importance. The pairwise correlations were calculated with both Pearson and Spearman methods^40^ and the *p*-values were used to verify that the correlations are statistically significant. We regard as important and unbiased features those with both Pearson's and Spearman's correlations above 0.4 and *p*-values below 10^−3^. Finally, each single molecule is represented by a 24-dimensional space of the highly pairwise-correlated descriptors (Table S2†). Thus, the molecular pairs are the concatenation of the individual vectors of each single molecule. All the labelled molecules were standardized to [0,1] using the scaling methods provided from sci-kit learn, such that all the numerical features will belong to the same range. The scaler is fitted to the known molecules that form co-crystals. Then the trained scaler is implemented to transform each molecule in the molecular pairs in both the labelled and the unlabelled datasets, such that there will be a consistency among them and the same molecules will get the same representation independent of which pair they belong to.

### Traditional one class classification

Eight different algorithms were selected from the PyOD and sklearn library representing the wide range of the one-class classification (anomaly detection) categories as described above: Gaussian Mixture Models (GMM), Local Outlier Factor (LOF), k-nearest neighbors (kNN), Isolation Forest (Iforest), One Class SVM (OCSVM), Histogram Based Outlier Score (HBOS), Cluster-based Local Outlier Factor (CBLOF) and Feature Bagging (with LOF as the basis algorithm) (ESI Section 2 and Table S3†).^41^ Each algorithm has its internal scoring function, depending on the cost function it tries to minimize. For achieving better predictive performance and ensuring the robustness of our method the models were combined in an ensemble way. For consistency with the GMM model from the scikit-learn library,^42^ the scores from the PyOD library were multiplied by −1 to have higher scores for the inliers and lower for the outliers. Each model was initially trained and optimized separately to provide an anomaly score to the input data. Then the scores of the pretrained models were normalised between [0,1] and averaged, following the methodology from the combo library^43^ so that the outputs become comparable.

### Hyperparameter tuning

As the performance of the algorithms is highly dependent on the choice of the hyperparameters, *i.e.*, algorithm variables, the optimization step is crucial for achieving the highest possible accuracy. The tuned hyperparameters of each method are presented in ESI (Table S3†). For the machine learning models, the optimization step is about searching for the hyperparameters with the lowest validation loss. Bayesian optimization was used *via* the Hyperopt library.^44^ The main idea behind Hyperopt is to get more points from the regions with high probability of yielding good results and less points from elsewhere. Hyperopt library was implemented for each of the eight algorithms from the PyOD & scikitlearn library,^41,42^ to find the best set of parameters to maximize the average accuracy of the k-fold cross-validation.

### Deep learning approach

Using the traditional one class classification algorithms as baselines, the application of a deep learning method was investigated for extending the dataset to the whole *n*-dimensional space (*n* = 3714, *i.e.*, 1857 descriptors for each molecule in the pair). In that way the predictions are not only based on a few pairwise correlated descriptors. That is very important as the co-crystal design problem is complex and thus higher-order interacting features might have a key role in the co-crystal formation. The main advantage of using a neural network in this context is that the extensive feature engineering part can be omitted, as the network can learn relevant features automatically. The most broadly used deep learning approaches for one class classification rely mainly on Autoencoders. An Autoencoder is a neural network that learns a representation of the input data by trying to accurately reconstruct the input with minimum error. It is considered to be an effective measure for separating inlier and outlier points.^45^ Autoencoders are used for learning the representation of the labelled data and then the unlabelled data are reconstructed using the same weights from the target class. The decision of whether a new datapoint is an inlier or an outlier is made based on the reconstruction error. High reconstruction error indicates that a sample is most probably an outlier, whereas when we have low reconstruction error the samples most probably belong to the same distribution as the labelled data. Autoencoders have the objective of minimizing the reconstruction error, but do not target one class classification directly. For designing a more compact methodology, the adapted approach incorporates both an Autoencoder for representational learning which is jointly trained with a Feed Forward Network targeting one-class classification.

### Deep one class architecture

The Deep Support Vector Data Description (DeepSVDD) architecture used in this paper is adapted from the work of Ruff *et al.*^29,46^ The aim of DeepSVDD is to find a data-enclosing hypersphere of minimum size, such that the normal datapoints will be mapped near the center of the hypersphere whereas anomalous data are mapped further away. The objective of DeepSVDD is to jointly learn the network parameters together with minimizing the volume of the hypersphere. The deep learning protocol followed by DeepSVDD is a two-step process. The first step, *i.e.*, the pretraining step, is composed by a Convolutional Autoencoder for effectively capturing the representation of the data. During the pretraining, the center of the hypersphere is calculated and is fixed as the mean of the network representations of the known data.^29^ During the second step, the latent dimension of the Encoder is connected to a Feed Forward Neural Network with the specific task of minimizing the loss function (distance from the center of the hypersphere). The same pretraining and training steps as in the DeepSVDD method were used for our problem settings, whereas the Convolutional Autoencoder was substituted with the SetTransformer Autoencoder adapted from Lee *et al.*^47^ The implemented set-input architecture uses a self-attention mechanism that allows the encoding of higher-order interactions and is able to directly perceive the order invariance among the pairs. All the known data (molecular pairs) are considered to belong to the hypersphere and they are scored based on their distance from the center, thus the lower the score the closer to the center and the more of an inlier is the data point. Likewise, the unlabelled data are assigned scores based on their distance from the pre-defined center. All the scores are multiplied by −1 and normalized from 0 to 1 so that they are comparable to the other models and give scores close to 1 for the inliers, whereas the points scored close to 0 are the anomalies.

### Model evaluation

The evaluation of the classification performance for one-class classifiers differs from multi-class classification as only the probability density of the positive class is known. That means that the model can only be optimized and validated by minimizing the number of positive class instances that are not accepted by the one-class classifier (false negatives).^27^ Opposed to the binary classifiers, where the decision of the class is made based on a set threshold, usually 0.5 (if a point scores below 0.5 it belongs to the first class else to the second), in one class classification the threshold is defined only from the known class. That is set using a parameter (here referred as contamination), which defines the amount of noise we expect to have in our known class. Herein, we accept that parameter as 0.05, meaning that 95% of the known data are inliers and only a very small part of them that deviated from the rest can be regarded as outliers. The evaluation of the models was performed using five-fold cross validation on the labelled dataset. The labelled dataset is split into five parts (folds) where 4/5 are used for the training and the remaining part is used for the validation. The process is repeated five times, each time selecting a different fold and the evaluation is performed using accuracy metrics from version 0.22 of the scikit-learn package. The final accuracy is calculated by taking the mean of the five accuracy scores of the validation set.

### Model interpretability

To better understand the features that are important for the neural network categorization of the molecular pairs in one class, we used SHAP (SHapley Additive exPlanations).^48^ This interpretability method is based on the calculation of the game theoretically optimal Shapley values, which are indicative of the contribution of each feature to the final prediction. One of the advantages of using SHAP is that it offers both local interpretability, by looking at how the features in each individual combination will affect the decision, as well as global interpretations, by aggregating the local values. We are thus able to know which features in a specific molecular pair affect the decisions more and extract general views on the features that influence and dominate the overall design of that type of material.

### Co-former ratio predictions

For the prediction of ratios between co-formers, the binary classification approach was implemented. The scikit-learn 0.22 version of the XGBoost classifier was trained on the known co-crystal molecular ratios. We are interested in detecting if a co-former combination will be found in 1 : 1 or higher ratio. The 1 : 1 molecular ratio was assigned to label ‘0’, whereas the higher ratios were labelled as ‘1’. As the majority of CSD co-crystals were found in 1 : 1 ratio we have an imbalanced dataset. To overcome this bias, SMOTE algorithm from the IBM package imbalanced-learn was used to generate artificial datapoints that could belong to the underrepresented class such that the two classes will become balanced.^49^ The optimum set of parameters were selected with the Hyperopt algorithm.

### Pareto optimization

Pareto optimization simultaneously identifies the optimal values in a set of parameters and was used to select and prioritise the co-formers to be experimentally tested. In our case the parameters that were optimised are the score from the model and the similarity to 7,7,8,8-tetracyanoquinodimethane (TCNQ). This two-parameter optimization was implemented to drive the decision making for the experimental screening.

### Euclidean distance and 2D visualization

The similarity between the experimentally synthesized co-crystals and the rest of the labelled dataset is measured using the Euclidean distance of the high-dimensional descriptor vectors. As such, the closest feature-wise known structures to the synthesised co-crystals were detected. Furthermore, the labelled dataset was projected in two dimensions using the Uniform Manifold Approximation and Projection (UMAP) algorithm.^50^ UMAP is a dimensionality reduction technique based on Topological Data Analysis and is aiming to preserve both local and global topological structure of the data. The UMAP parameters were selected in a way such that the maximum of the distance is preserved when moving from the higher to lower dimensions. The distance preservation was measured by calculating the Pearson correlation coefficient of the distance matrix using the whole dimensionality and the distance matrix after the dimensionality reduction. The most effective settings were as follows (n_neighbours = 80, min_dist = 0.1, Euclidean distance metric) resulting in Pearson correlation coefficient of 0.748.

## Experimental section

### Materials and chemicals

9,10-Dicyanoanthracene (CAS RN:1217-45-4, >98.0%) was purchased from TCI UK Ltd.; Pyrene (CAS RN: 129-00-0, >97%) was purchased from TCI UK Ltd.; 6*H*-benzo[*c*]chromen-6-one (CAS RN: 2005-10-9, 96%) was purchased from Fluorochem Ltd. All chemicals were used directly without further purification. No safety hazards were encountered during the described Experimental procedures.

### Co-crystal growth

#### Preparation of co-crystal pyrene-6*H*-benzo[*c*]chromen-6-one (1)

Pyrene (20 mg, 0.1 mmol) and 6*H*-benzo[*c*]chromen-6-one (20 mg, 0.1 mmol) were dissolved in dichloromethane (6 mL) and heated at ∼45 °C for 16 hours under continuous stirring. After heating for 16 hours the resulting mixture was filtered (using Whatman filter paper). The filtered solution was allowed to stand at room temperature for slow evaporation in open air (partially covered). Colourless plate-like crystals of the co-crystal pyrene-6*H*-benzo[*c*]chromen-6-one (1) appeared after 3–4 days.

#### Preparation of co-crystal pyrene-9,10-dicyanoanthracene (2)

Co-crystal 2 was synthesized following an analogous procedure as described for 1 using pyrene (20 mg, 0.1 mmol) and 9,10-dicyanoanthracene (23 mg, 0.1 mmol). Orange column-like crystals of the co-crystal pyrene-9,10-dicyanoanthracene (2) appeared after 1–2 days.

### Characterization methods

Diffraction data for co-crystal 1 and 2 were collected using a Rigaku AFC12K goniometer employing graphite monochromated Mo Kα (*λ* = 0.71073 Å) radiation generated from a Rigaku 007HF molybdenum rotating anode microfocus X-ray target source and using a Saturn 724+ CCD detector. All data reduction and processing was performed using the CrysAlisPro software package and empirical absorption corrections using spherical harmonics were implemented in the SCALE3 ABSPACK scaling algorithm.^51^ The structures were solved using either direct^52^ or dual-space^53^ methods and refined by full matrix least-squared on F^2^ with SHELXL2015.^54^

## Results

The one class classification framework as expressed by the various implemented algorithms is discussed below. The two different workflows followed involve (i) the application of traditional algorithms designed for one class classification after extensive feature engineering to reduce the dimensionality of the problem and (ii) the design of a deep learning methodology for handling the specific co-crystals dataset, considering them as pairs of data, and avoiding feature engineering by solving the problem in higher dimensions. As traditional algorithms we are referring to the provided algorithms from PyOD/scikit-learn libraries and as Deep One Class to the deep learning model that was built by combining an Attention-based Encoder and DeepSVDD network. In both workflows a two-step process is employed. First the algorithms were trained and optimized on the known data and then they were used for scoring both the labelled and unlabelled molecular combinations. High scores are an indication for inliers, whereas the lower the score the higher the probability for a point to be an outlier.

**Fig. 2:**
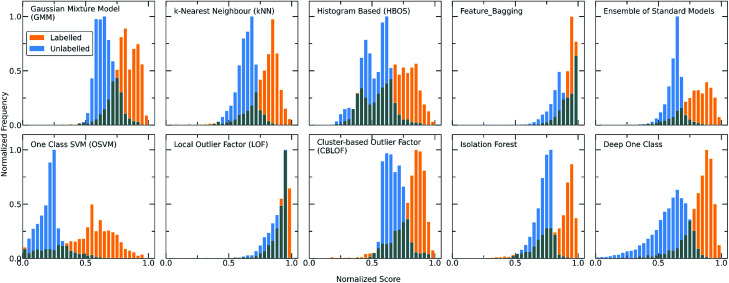
Score distributions of the labelled (orange) and unlabelled (light blue) data using all the discussed one-class classification algorithms. Each algorithm employs a different scoring function to assign scores to the molecular combinations, giving in all the cases higher scores to the labelled combinations (training set) whereas only a certain part of the unlabelled combinations (test set) receives high scores and can be regarded as inliers. As the number of unlabelled data is significantly higher than the number of known data, the *y* axis (showing the frequency) is normalized to [0,1] (for visualization purposes). The output scores of all the models are also normalized to [0,1]. A clearer and more definite separation among the two different datasets can be observed for both the Ensemble and Deep One Class methods, with Deep One Class covering a bigger range of scores and thus enabling a better separation.

The score distribution of both the labelled and unlabelled data for all the implemented algorithms is presented in Fig. 2. It can be observed that the labelled and unlabelled data form two overlapping classes. The unlabelled data consist of both positive and negative examples in an unknown proportion. Consequently, a certain part of the unlabelled data is expected to belong to the known class *i.e.*, are inliers. Moreover, in the labelled data there is a small proportion of examples that significantly differs from the rest of the data and is regarded as noise of the normal class, *i.e*., outlier examples. The impact of the class noise is mitigated using one class classification, as a percentage of the labelled data are regarded as outliers during the hyperparameter optimization process (see Methods). In general, for both the traditional and deep one class classification workflows, (i) the labelled data show higher scores with all the methods, (ii) each method has a different way of scoring the samples and deciding for whether a point is a normality or anomaly and (iii) only a certain part of the unlabelled data receives high scores. Differences arise between the algorithms because each is based on different definitions on what an oulier/inlier means, *i.e.*, an outlier is a point far from other points (kNN), an easily splittable point (Isolation forest), not part of a large cluster (Cluster-based outlier detection) or a point far away from the center of a hypersphere (DeepSVDD). Moreover, the traditional approaches differ from the deep approach in terms of the dimensionality of the features and the way the molecular pairs are perceived by the models. To this end, the two workflows are investigated separately.

### Traditional one class classification

The main characteristic of the traditional algorithms is the need for dimensionality reduction. For that reason, the important features were manually extracted based on molecular complementarity (highly pairwise correlated descriptors among the two molecules). The resulting 24 descriptors include the number of bonds (*n*BT), the number of heteroatoms (*n*Het), electrotopological characteristics (MAXDN, MAXDP, DELS), *i.e.*, combination of electronic features and topological environment for given atoms, the topology and polarizability of the molecules (*e.g.*, SpMax4_Bh(m), SM1_Dz(e)). A detailed list with the selected descriptors and their correlations can be found in the ESI (Table S2†). As each molecule is represented by a vector belonging to these 24 descriptors, the molecular pair is the concatenation of two vectors. Importantly, for extensively covering the co-crystal space, the two individual vectors were concatenated in both directions in the training set as the input should be invariant to the position of the molecule in the vector (Fig. S11†). For instance, in the representation of the pyrene-TCNQ pairs (co-crystal: PYRCBZ02), the score should be the same whether the input is given as pyrene-TCNQ or TCNQ-pyrene.

**Fig. 3:**
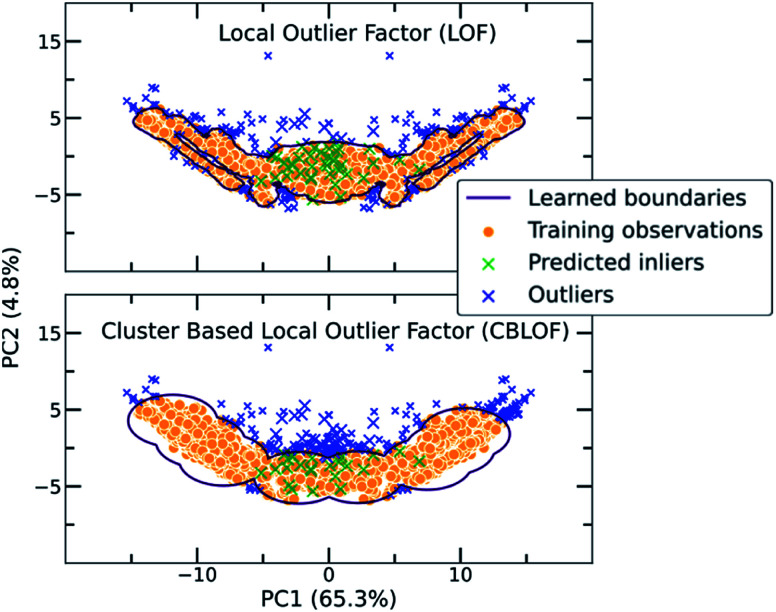
Two examples of traditional one class classification algorithms, namely Local Outlier Factor (LOF) and Cluster Based Local Outlier Factor (CBLOF), visualizing the way the boundaries around the co-crystal space are drawn. The labelled co-crystals dataset was projected to two dimensions using Principal Component Analysis (PCA). PCA was applied after the calculation of the scores in all dimensions. 50 random points of the unlabelled dataset were selected for visualizing their position in the two-dimensional space. Points marked as green crosses are identified as inliers, whereas blue crosses are the outliers. It is observed that each algorithm is implementing a different scoring function and thus the decision boundaries that separate inliers from outliers differ.

A short description about how each of the traditional one class classification algorithms regards an outlying point as well as the selected hyperparameters can be found in the ESI (Table S3†). As a showcase, the way two different algorithms differentiate inliers from outliers is presented in Fig. 3, where the co-crystal dataset was projected in the two dimensional space using Principal Component Analysis (PCA).^55^ The bidirectionality of the training set is also observed by the symmetry of the projected data.

**Fig. 4:**
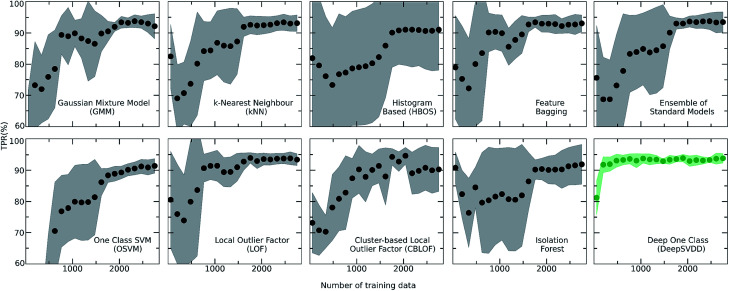
Learning curves of all the implemented algorithms showing the performance of the models while the size of the training set increases. The highlighted grey area represents the standard deviation of each model. The validation metric used is the True Positive Rate (TPR), *i.e.*, number of correctly predicted inliers/total size of the training set in each fold of the *k*-fold (*k* = 5) cross validation. It is observed that the Deep learning model (DeepSVDD) outperforms the traditional algorithms as it has higher accuracy and low standard deviation.

For achieving more reliable and robust predictions, the eight traditional one class classification algorithms were combined in an ensemble way by averaging their output. Thus, the final scores of both the labelled and unlabelled data were calculated by the ensemble. The distribution of the ensemble scores, after being normalized to [0,1], are shown in Fig. 2. It is observed that the ensemble separates better the labelled from the unlabelled data in comparison to the individual traditional algorithms. That is an indication that the ensemble is a better classifier as the balance point above which the amount of labelled data is maximum and the number of unlabelled data is minimum is easier found.^56^ Numerically, that means that the scores around 0.7 can be regarded as good and promising scores for identifying novel molecular pairs and that the 5434 out of the 21 736 possible combinations are the top scored combinations. The performance of each algorithm was calculated by the True Positive Rate (TPR), defined as the average of correctly predicted inliers resulting from five-fold cross validation. As illustrated in Fig. 4, all the algorithms achieve a high accuracy on the True Positive Rate and perform quite well on unseen data. However, the Gaussian Mixture Model (GMM) and the Histogram-based Model (HBOS) are less robust as indicated by the higher variation in the total accuracy (Fig. 4 and S12†). The effect that the addition of data in the training set has on the accuracy is also investigated after calculating the learning curves of each algorithm. For the correct sampling of the bidirectional dataset in the different training set sizes, it should be ensured that equivalent pairs exist in each subset.

### Deep one class approach

Despite the fact that the aforementioned one class classification models show high accuracy (%) as indicated by the True Positive Rate (Fig. 4), they require substantial feature engineering. By decreasing the dimensionality, the complexity of the model is lowered. However, substantial information might get lost and some key descriptors might be removed. These limitations led to the development of deep learning approaches for automatically learning relevant features with the specific purpose of one-class classification.^29,57^ As such, a deep learning model was employed for processing the co-crystals dataset.

**Fig. 5:**
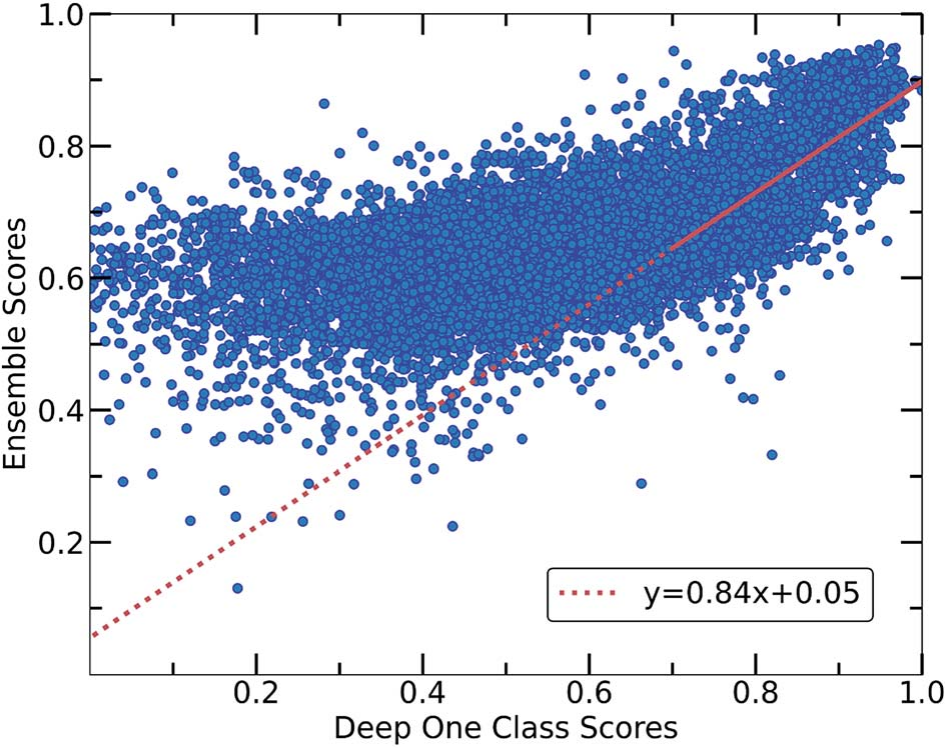
Correlation between the scores of the Ensemble and Deep One Class methods. Both workflows show a good correlation in the general distribution of scores, with Deep One Class covering a wider range of scores and enabling in that way a better separation between inliers and outliers. A significant correlation exists for the high score pairs, showing that both methods could be reliable in the high-score region.

The whole feature dimensionality is the input to the deep learning model concatenated in a way such that the order invariance is preserved (see SetTransformer in the ESI† Section 3). The way the SetTransformer extracts the features is key for capturing the complexity of the problem. SetTransformer ‘looks’ in all the features across a single molecule as well as in all the features of the pairing molecule. In that way the latent dimension holds information for the relation between the descriptors for each molecular pair. A detailed description of the way SetTransformer captures the relations among the descriptors is shown graphically in the ESI (Fig. S9†). It can be observed that the deep learning model outperforms the traditional methods as the accuracy (%) is higher and the standard deviation is minimum. Moreover, there is a clearer separation of the labelled from the unlabelled data and thus a balance point between them was more easily found (Fig. 2, DeepSVDD). The two workflows are also compared scores-wise (Fig. 5). It can be seen that there is a good agreement (correlation) in high scores, whereas in the lower scores area there is not a clear correlation as the ensemble method gives a narrower range of scores and higher scores for low-scoring examples in the Deep case.

In every classification problem, a threshold should be specified above which the datapoints that belong to the normal class can be found. We set that threshold at 0.7 and thus all the molecular pairs with scores higher than 0.7 are regarded as reliable inliers with a high probability to exist. That threshold was selected based on the good agreement between both workflows for scores above 0.7. Moreover, it is a good balance point as the majority of the labelled data receive scores above that threshold whereas only the top quartile of the unlabelled data can be found in that area. In cases where a better separability is achieved,^58^ the amount of misclassified data (FP: False Positives) is minimized significantly, thus the selection of the threshold (on 0.7) could be regarded a reasonable decision boundary.

### Predicted molecular pairs

After testing and evaluating the possible algorithms for one class classification, the better performing method was employed for the final decision on the ranking. Taking into consideration not only the better accuracy of the deep approach, but also that there was no need for extensive feature engineering, the better separation of the labelled and unlabelled data and the clearer decision boundary, the final ranking is based on the deep learning method.

**Fig. 6:**
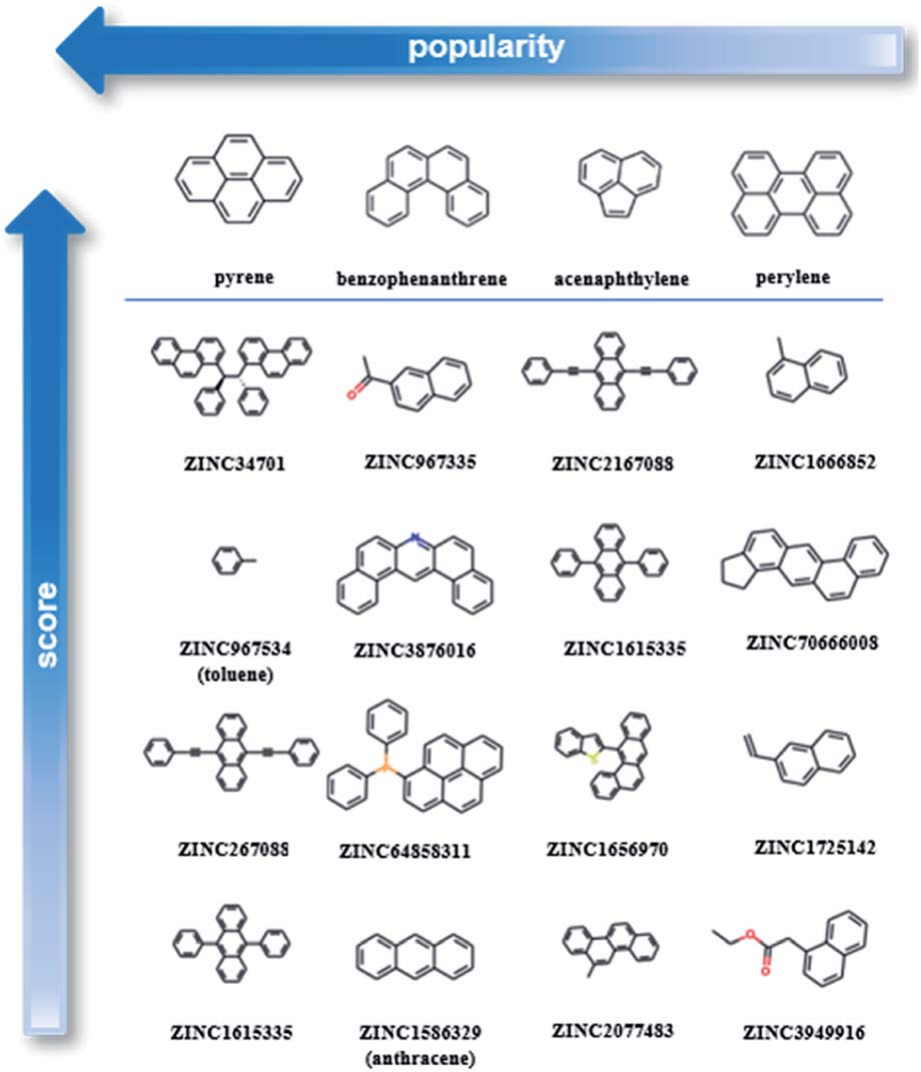
Molecular pairs formed by the most popular co-formers as predicted using deep learning approach. Pyrene was identified as the most popular co-former as the majority of the possible pyrene co-crystals were assigned with high scores. The arrows indicate the direction of higher score (vertical arrow) and higher popularity (horizontal arrow).

As our training dataset consists of many co-crystals with one aromatic solvent (*i.e.*, benzene or toluene) and one highly branched molecule (*i.e.*, molecules with nonlinear backbone), it is expected that the top scored pairs will follow the same trend. As shown in Fig. S14† the two top scored predictions are those between toluene and two of the most highly branched molecules of the ZINC15 list. As the purpose of the model is to learn the underlying patterns on the labelled dataset and then detect molecular pairs with similar patterns in the unlabelled dataset, that scoring is quite reasonable. If we want to remove the solvents and look at other high-score subsets, we can perform a search under different constraints, such as for finding the higher scored combinations (i) after removing the one ring molecules, (ii) after removing both the one-ring molecules and molecules with heteroatoms and (iii) when looking at the good combinations containing one of the eight starting PAHs. Detailed tables containing high-ranking pairs of possible categories of interest with their scores can be found in ESI (Tables S5–S11†). The popularity (frequency) of the co-formers forming high-scored pairs is also measured by counting the number of times each co-former appears in the pairs of the top quartile. The most frequent co-formers in their higher scored pairs are presented in Fig. 6. As shown the co-formers that appear more frequently in the predicted dataset are pyrene, benzophenanthrene, perylene and acenaphthylene.

### Model interpretability

The reliability of any machine learning model is enhanced when the model's decisions are related to physical properties. Following the traditional one class classification workflow, the features associated with the final predictions are already known after the extensive feature engineering process. On the other hand, an understanding about the features that played a key role in the deep learning approach is a more challenging task, as the complexity of the model is higher. Using the Shapley Analysis, the feature weights are expressed as Shapley values. A detailed description of the Shapley workflow can be found in the ESI.† To this end, features that play a key role in the scoring for the deep learning approach are retrieved and analysed. The aim of this process is to identify molecular properties or characteristics that might provide a chemical understanding to the model's decisions and assist the experimental screening process. As for many of the Dragon descriptors it is hard to extract a physical meaning, the correlations among the most significant descriptors with those that are more general and understandable are calculated.

According to Shapley analysis, the most important features that the inliers have in common and dominate the decisions are related to the descriptors B06[C–C], ATS6i, B08[C–C], ChiA_Dz(p), Eig06_AEA(dm) and SpMin5_Bh(s). Whereas, B06[C–C] and B08[C–C] can be easily related to the molecular length, as they describe the topological distance between two carbon atoms, *i.e.*, the presence of connected carbon atoms at specific positions on a molecular graph, the other descriptors are not directly related to a molecular property, for that reason, their physical meaning is extracted after calculating the correlations between them and the other Dragon descriptors, that are higher than 75% (Table S13†). Interestingly, they are highly correlated with more general and easily accessible molecular properties. These chemically meaningful descriptors refer to (i) electronic properties, such as the sum of first ionization potentials (*S*_i_), sum of atomic Sanderson electronegativities (*S*_e_), sum of atomic polarizabilities (*S*_p_) (ii) molecular size, such as McGowan volume (*V*_x_), sum of atomic van der Waals volumes (*S*_v_), (iii) molecular shape, regarding the molecular branching (Ram, eta_B), (iv) polarity (Pol, SAtot) and (v) molecular weight (*M*_W_). The correlations and a more detailed description of these descriptors are summarized in ESI (Table S13†). Interestingly, these descriptors are also relevant to the majority of the extracted descriptors after the feature engineering process (Table S2 and S13†).

**Fig. 7:**
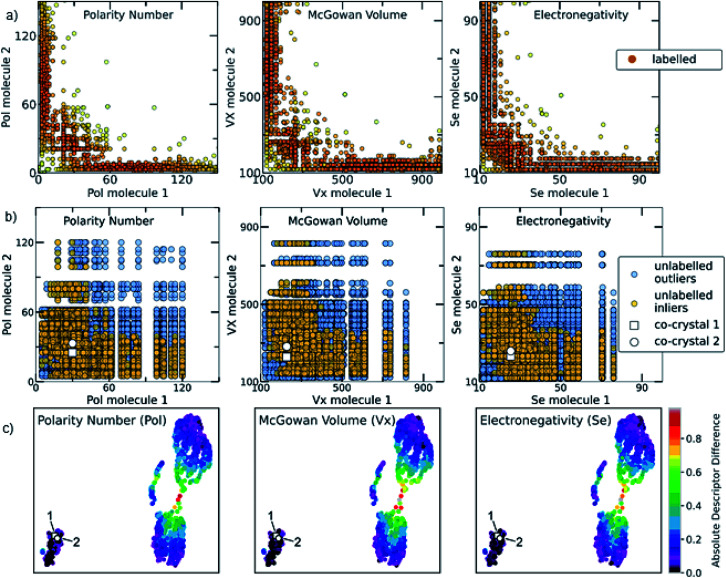
(a) Scatterplots showing the distribution of representative descriptors among the molecular pairs on the labelled dataset, extracted from CSD. The plotted descriptors are those identified as the most general and highly correlated to the descriptors extracted using the Shapley analysis (see Model interpretability and Section 7 in the ESI†). (b) The distribution of the same descriptors for the unlabelled data is shown. Blue circles represent the whole unlabelled dataset extracted from ZINC15 (21 736 points) and yellow-orange represent the top quartile of the unlabelled data having scores above 0.7 and are regarded as inliers. It can be clearly seen that the predicted inliers follow the distribution of the labelled dataset, especially in the densest area. This is an indication that the deep learning model can effectively learn the trends of the labelled data and is able to score the unlabelled data based on the significant patterns of the labelled data (training set). The white square and white circle denote the two experimentally synthesized co-crystals (see Experimental section in Methods and *in silico* prediction and experimental realization section in Results). Both synthesized co-crystals lie into the densest area regarding the polarity and electronic descriptors. (c) UMAP 2D illustration of the labelled dataset containing the extracted from CSD co-crystals and the projection of 1 and 2 to the known co-crystal space. The datapoints are colored according to the absolute difference of the descriptors identified as important. It can be observed that the whole dataset consists of two main areas: one area in which the molecular pairs have molecules with similar properties, *i.e.*, shape, polarity and electronegativity, in which 1 and 2 belong and a second area involves molecular pairs with significant difference in these properties.

The relationship among some of the important interpretable descriptors in the molecular pairs is illustrated in Fig. 7 and S22–S26† for both the labelled and the unlabelled datasets. It should be noted that the distribution trend of the labelled and unlabelled dataset can change according to the studied descriptor. In Fig. 7a, the dominating trends on the labelled dataset can be observed with darker orange color indicating the densest area with more molecular combinations. Two main areas are extracted from the labelled dataset. The first area includes molecular pairs where both molecules have low values of the same property, *e.g.*, in the Polarity plot the area 0 < Pol < 60, where both molecules could have similar values. The second area includes molecular pairs with higher difference on their property values, *i.e.*, when one molecule has a low value of one descriptor then the pairing molecule has a higher value for the same descriptor, complementing the first molecule. This observation is also verified by the UMAP visualization of the dataset (Fig. 7c, see also Euclidean distance and 2D visualization section in Methods), where the two discrete areas are captured. In the UMAP plot the smaller cluster to the bottom left represents the area in which molecules with similar values are found, the two synthesized co-crystals 1 and 2 (*vide infra*) are located in this area. This observation does not apply for all the available descriptors (see Fig. S35†). These observations are also compared with a previous study by Fabian that focused on the CSD co-crystal dataset.^40^ Fabian's statistical analysis of the data at that time concluded that the majority of co-crystals in CSD (CSD, version 5.29, November 2007) are formed by molecules of similar size and polarity.^40^

Our analysis shows a more complex scenario. Size, shape and polarity, identified as important factors of co-crystallization, have similar property values only in the low value region, in agreement with Fabian's conclusions. However, in the high value regions the trend drastically changes; molecules having high size, shape and polarity values tend to pair with molecules having low values of these parameters. It should be noted that Fabian had also observed and chosen not to focus on the smaller subset where the co-formers are dissimilar due to their lack of relevance for his domain of focus, *i.e.* pharmaceutical co-crystals and predominantly hydrogen-bonding ones. Our study needed to consider the dissimilar pairs as in PAHs co-crystals feature dissimilarity is common. Interestingly, our machine-learning modelling approaches were capable of taking into account a more complex relationship between descriptors, so it wasn't necessary to simplify the analysis to just one subset of the co-crystal dataset in terms of descriptor space. As shown in Fig. 7, the distribution of property values in the high scoring pairs (inliers) in the unlabelled dataset (Fig. 7b) are predicted to follow the same patterns at the labelled dataset (Fig. 7a) indicating that the deep learning model effectively learnt the trends of the labelled dataset and was able to score the unlabelled dataset based on those trends.

The dominating features as expressed with global Shapley values can give a general picture of the dataset. However, it should be noted that a better understanding for specific groups of pairs that might be of interest can be attained when focusing on them explicitly. The advantage of using Shapley values is that local explanations are given to each individual molecular pair or to a subset of interest among the molecular pairs. As a case study, the pyrene-cocrystal family is investigated, aiming to extract some general patterns about the important molecular characteristics that drive a good match for co-crystal formation with pyrene (Fig. S21†). The dominating features in the known co-crystals with pyrene are presented in Fig. S21.† It was found that the existence of heteroatoms such as oxygen and/or nitrogen groups on various topological distances, as indicated by the B03[C–O], B02[C–O], B02[C–N] and B05[C–N] descriptors or the existence of halogen atoms as indicated by the X% descriptor (Fig. S21†) play a key role in the assignment of high scores in these combinations. Furthermore, the aromaticity as represented by the ARR descriptor was a factor that contributed to high scores.

The key findings from the model interpretation and feature analysis can be summarized below:

(i) Shape, Size and Polarity were detected as important factors for co-crystallization, which is in accordance with previous understanding about the co-crystals of CSD. However, Fabian's observations are relevant only for low values of these properties. We observe that there are no cases in the labelled data and in the inlier part of the unlabelled dataset where both molecules have very high values of polarity and/or volume. This could be an indication for factors prohibiting co-crystallization. In cases, where high polarity or volume values are assigned to one molecule the pairing molecule usually has a low value of that property.

(ii) PAH co-crystals seem to deviate from empirically established rules and trends observed for organic co-crystals in general. Thus, a deeper understanding of their properties can only be gained when they are studied separately. As PAHs lack hydrogen bonding, other types of interactions appear as stabilizing factors for co-crystallization. For instance, in the pyrene-based co-crystals the existence of O and/or N groups has been identified as a key parameter as the majority of molecules that form co-crystals with pyrene contain these groups. The existence of these groups can drive the formation of C–H⋯N, C–H⋯O and C–H⋯X (X = halogen groups) which will probably stabilize the co-crystal formation.

(iii) There is not a ‘magic’ descriptor or set of some descriptors that can directly predict co-crystallization. The synergy among many descriptors will led to a successful combination. The more parameters, and the more the relationships among them, that are taken into consideration, the more reliable the predictions we can attain. This is the reason the implementation of the appropriate ML tools could save significant amount of time and guide the synthetic work, as this is the only way where the relationship among a large number of properties is simultaneously considered.

(iv) The selection of pairs for co-crystallization screening is a challenging task. However, when there is a certain category of interest, we can extract the important features that dominate the known co-crystals and then select for experimental screening molecules that both have high score (as the score is based on the interaction among all the known descriptors) and some of the properties that are extracted as important. It should be noted that a pair of molecules might have a good score and good values of a property of interest but not give a successful result as a property that is not considered from the model is affecting the experiment, *e.g.*, the solubility of the molecules in several solvents is not considered in the ML model at the moment.

### Molecular ratios prediction

**Fig. 8:**
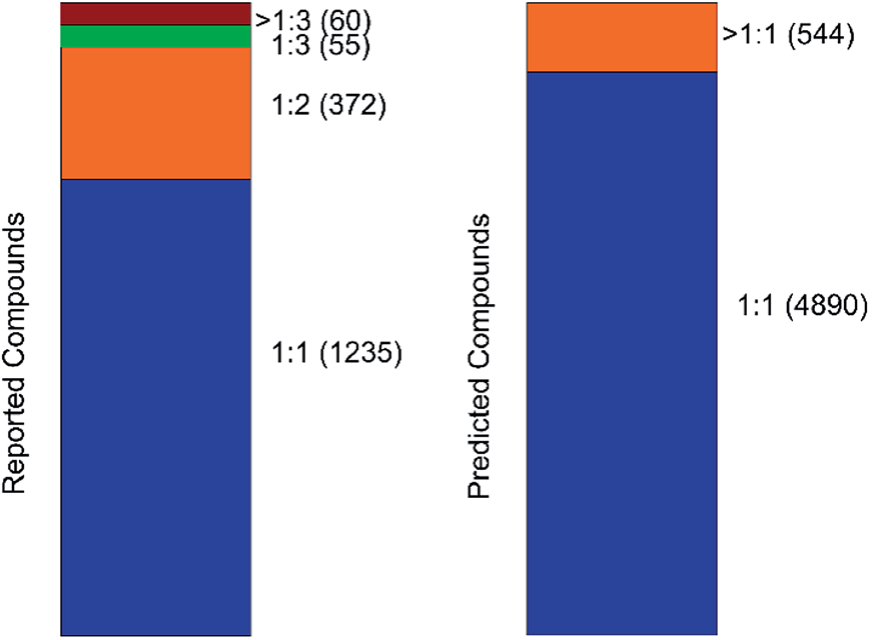
Barcharts illustrating the molecular stoichiometry on the reported (left, labelled dataset) and on the predicted (right, inliers) compounds of the co-crystal dataset. It can be observed that the dominating ratio is 1 : 1, resulting in a highly imbalanced dataset towards molecular ratios.

An important parameter that should be taken into consideration in co-crystals design is the stoichiometry of the co-formers. The molecular ratio is going to affect the crystal packing and thus contribute to possible materials properties. To this end, the labelled co-crystals dataset was further tested for molecular ratio prediction. The molecular ratio of all the combinations was extracted during the labelled dataset construction (see Methods). The dominating ratio in the dataset is 1 : 1 as shown in Fig. 8, resulting in a highly biased dataset towards the molecular ratios. The problem setting was adjusted for performing binary classification and investigated whether the molecular ratio is going to be 1 : 1 or higher. We assigned label ‘0’ to all the molecular pairs having 1 : 1 ratio and ‘1’ otherwise. The problem was solved using SMOTE technique for balancing the two classes of the dataset such that they have equivalent amount of data having 1 : 1 ratios and data having ratios different to 1 : 1.

The labelled dataset was split into a training and a test set with the latent representation being the input to a binary classifier. The model showed strong predictive power, with accuracy on both the training and test sets of about 94% and no overfitting on the training data (Fig. S19†). The same model was then implemented for predicting the molecular ratios in the inlier pairs. The high accuracy of the ratio prediction on the unlabelled dataset were further verified by the experimental results. The ratio of compound 1 was predicted to be different from 1 : 1 and indeed the ratio of the synthesized co-crystal was found 1 : 2. Furthermore, the ratio of compound 2 was predicted to be 1 : 1 and likewise it was found to be 1 : 1 experimentally.

### *In silico* prediction and experimental realization

**Fig. 9:**
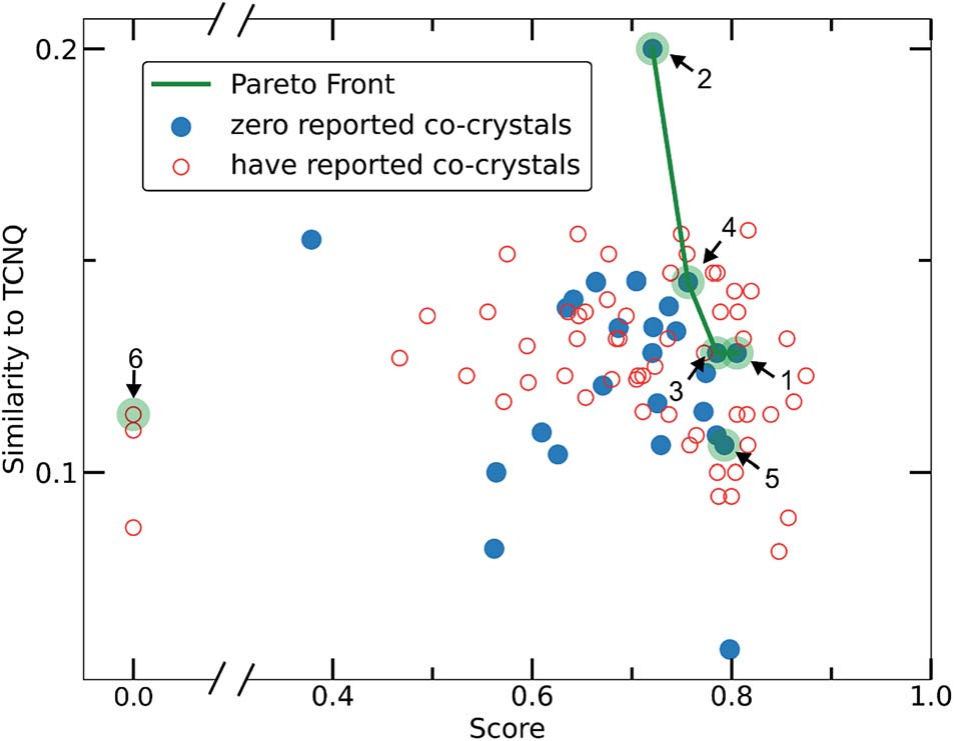
Scatterplot illustrating the selection criteria for the experimental screening process. Pareto optimization was implemented having as the main task the optimization of two objectives, (i) the score of the deep learning model and (ii) the Tanimoto similarity to TCNQ. Each point represents a molecule that could be used as the second co-former in pyrene co-crystals. Red empty circles stand for molecules that are already known to form co-crystals in the CSD, whereas molecules represented with filled blue circles have zero reported co-crystals. The molecules selected and experimentally tested are highlighted in green circles. 1–4 are on the pareto front and 5 is the highest scoring co-former off the Pareto front. 6 is an outlier.

To narrow down the selection of potential co-formers from those identified using the single class classifier model, we chose pyrene as a fixed component because both the existing data (*i.e.*, CSD database) and the model output reveal its popularity and versatility as a co-former, *i.e.*, pyrene can co-crystalize with a diverse range of molecules forming high score pairs. The total 207 possible pyrene-containing co-crystals identified by the single class classifier model (Fig. 9) were narrowed down to a subset of 29 pairs where the second co-former has zero examples of known co-crystals with any other molecule (blue points in Fig. 9). Pareto optimization (see Methods and ESI† in Section 8) was used to identify the most suitable candidate co-formers for experimental investigation by simultaneously optimizing (i) the highest predicted score of the pyrene co-crystals and (ii) the highest Tanimoto similarity with the well-known acceptor molecule 7,7,8,8-tetracyanoquinodimethane (TCNQ), which is extensively studied for its interesting electronic properties both in the crystalline form and as a co-crystal.^21,26,59–65^ From the Pareto front (Fig. 9 green line) 1–4 are identified as the optimal candidates and 5 is the highest scoring co-former off the Pareto front.

**Fig. 10:**
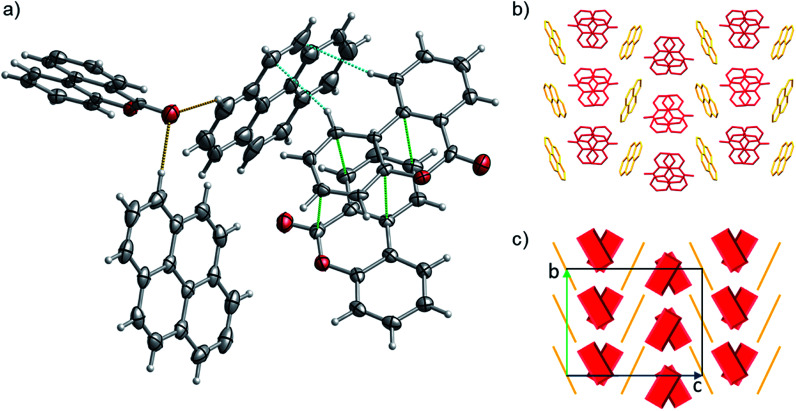
(a) Portion of the crystal packing of 1. Horizontal axis, *c*; vertical axis, *a*. Atom color code: carbon, grey; oxygen, red; hydrogen, light grey. The displacement ellipsoids are drawn at 50% probability level with the hydrogen atoms showed in the ball and stick mode for clarity. The weak C–H⋯π interactions between neighbouring molecules are represented as dashed light blue lines, C–H⋯O the interactions as dashed brown lines and the distances between 6*H*-benzo[*c*]chromen-6-one dimers are represented as dashed green lines. (b and c) representation of the γ-type crystal packing between the pyrene (highlighted with yellow color) and 6*H*-benzo[*c*]chromen-6-one (highlighted with red color) molecules.

**Fig. 11:**
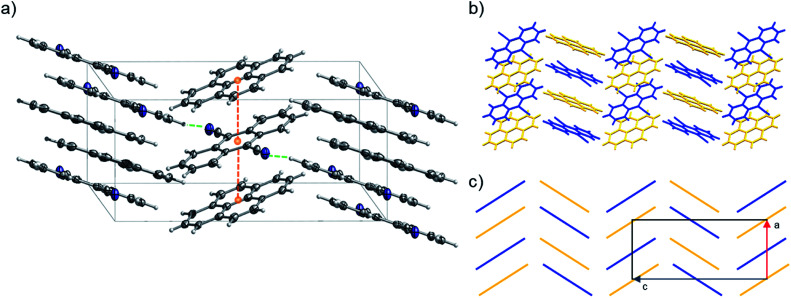
(a) Portion of the crystal packing of 2 viewed in perspective along the [010] direction. Horizontal axis, *c*; vertical axis, *a*. The displacement ellipsoids are drawn at 50% probability level with the hydrogen atoms shown in the ball and stick mode for clarity. Atom color code: carbon, grey; nitrogen, blue; hydrogen, light grey. The weak C–H⋯N lateral interactions between neighbouring molecules are represented as dashed green lines, the distances between the centroids (orange spheres) of pyrene and 9,10-dicyanoanthracene molecules are represented as dashed orange lines. (b and c) Representation of γ-type flattened herringbone crystal packing between the pyrene (highlighted with yellow color) and 9,10-dicyanoanthracene (highlighted with blue color) molecules.

Out of the Pareto optimal candidates, pyrene-6*H*-benzo[*c*]chromen-6-one (1) and pyrene-9,10-dicyanoanthracene (2) (Fig. 9) have been successfully isolated as co-crystals (see Experimental section for the synthetic details and Fig. 10 and 11) with 1 and 2 being the first examples of co-crystals containing 6*H*-benzo[*c*]chromen-6-one and 9,10-dicyanoanthracene as co-formers.

The next Pareto optimal candidates for experimental investigation were pyrene-1,2,3,4,-tetrahydrophenanthrene-4-one (3), pyrene-1-vinyl-naphthalene (4) and pyrene-9-phenylanthracene (5) (Fig. 9). However, 3, 4 and 5 did not lead to any new co-crystals with pyrene when following an analogous synthetic procedure to that of 1 and 2. While 3, 4 and 5 could be seen as potential negative results, and could be fed back in to the model to improve its predictive power, it should be specified that more rigorous screening of the crystallization conditions is required. The current machine learning model does not take into consideration all possible chemical factors that might affect the reaction outcome (*e.g.*, solubility of the co-formers). For instance, working with 5 we noticed that the physical form of 1-vinyl-naphthalene is liquid at room temperature and shows low miscibility with pyrene under the conditions tested. Following a different synthetic approach and using 1-vinyl-naphthalene and/or pyrene in excess might be a successful way towards the predicted co-crystal.

We considered it important to also test combinations that were predicted as outliers where the co-formers have numerous reported co-crystals. The pair pyrene-triphenylene (6, outlier score 0.0, Fig. 9) satisfies these criteria, since pyrene has 130 examples of known co-crystals and triphenylene has 20 reported examples in the CSD database, but the pair (pyrene-triphenylene) is not known as a co-crystal. Following a similar synthetic procedure using dichloromethane (a common solvent choice for the formation of known pyrene-containing and triphenylene-containing co-crystals) as described for 1 and 2 (see Experimental section in Methods), did not lead to any new phases. Nonetheless, for 6 a more rigorous screening of a wider range of experimental conditions should be tested. We recognize that six examples do not provide statistically significant evidence to fully validate the model experimentally. However, this initial experimental screening was performed for the exemplification of the model and it is noteworthy that two novel co-crystals with high scores were synthesized.

Finally, our study plays a key role in the expansion of knowledge around co-crystallization as it points that the existence of heteroatoms (such as oxygen and/or nitrogen), the shape of the co-formers and the extent of branching are the most dominating structural factors that synthetic chemists should take in consideration when working in the formation of pyrene based co-crystals.

### Description of crystal structures

The detailed crystallographic data for co-crystal 1 and 2 are listed in Table S15 in the ESI.† Pyrene-6*H*-benzo[*c*]chromen-6-one co-crystal (1) (CCDC ref. 2014577) shows a 1 : 2 stoichiometry of pyrene to 6*H*-benzo[*c*]chromen-6-one, notably verifying what was predicted by the one class classification approach (*i.e.*, 1 is predicted in a higher than 1 : 1 ratio). 1 has a complex packing, stabilized by both π–π stacking and T-shape interactions (Fig. 10 and S27–S28†), as unveiled taking advantage of Aromatics Analyser tool embedded in Mercury.^66^ Its structure can be classified as γ-type, where infinite stackings of the 6*H*-benzo[*c*]chromen-6-one molecules are alternated by ribbons of pyrene molecules in a A-BB-A motif.

Pyrene-9,10-dicyanoanthracene co-crystal (2) (CCDC ref. 2014576) crystallizes with a 1 : 1 stoichiometry confirming the predicted ratio (*i.e.*, in 2, the stoichiometric ratio of pyrene and 9,10-dicyanoanthracene was predicted to be 1 : 1). 2 can be classified as a γ-type structure with each infinite stack consisting of alternating pyrene and 9,10-dicyanoanthracene molecules (Fig. 11 and S29–S32†). Weak C–H⋯N lateral interactions between (i) molecules of pyrene and 9,10-dicyanoanthracene and (ii) molecules of 9,10-dicyanoanthracene, stabilize the stacks (Fig. 11a and S29†).

### Structural comparison with crystal structures in CSD

After the successful synthesis of 1 and 2, a comparison with the already known co-crystals was performed. 1 and 2 are the first examples of co-crystals containing 6*H*-benzo[*c*]chromen-6-one and 9,10-dicyanoanthracene as co-formers. For this reason, the crystal structures of 1 and 2 were initially compared to the pyrene-based co-crystals found in the CCDC database, specifically to the 130 reported entries (the list of pyrene co-crystal and their structural details can be found in Table S16 and Fig. S33 of ESI†). The majority of the pyrene co-crystals, together with 1 and 2, belong to the same cluster in the UMAP 2D visualization of the labelled dataset (Fig. S34 and S35 of ESI†), namely the one characterized by similar properties of shape, polarity and electronegativity as found in the model interpretation (see Fig. 7). Interestingly, both 1 and 2 adopt the γ-type motif, a complex arrangement where both stacking and T-shape interactions coexist. This occurrence is in contrast to what is reported for the 130 pyrene-based co-crystals (see Fig. S33 in ESI†), which usually crystallize in herringbone or β-type packing, 58% and 34% respectively. Hence, using one class classification approach to predict new materials allowed us not only to identify new co-formers and to synthesize two new co-crystals, but also to explore and to enlarge the rare subgroup (∼8%) of γ-type pyrene co-crystals. Our attention then moved to the most similar known co-crystals. To do that the Euclidean distance (*i.e.*, the distance between two vectors in the Euclidean space) between the vectors of the synthesized pairs and the vectors of the known molecular pairs in the labelled dataset was calculated. The resulting closest known pairs are presented in Fig. S36 of ESI.†1 is compared to the eight most similar co-crystals in the CSD database (Table S17 and Fig. S36 in ESI†).^67–72^ Contrary to 1, all eight compounds are in a 1 : 1 stoichiometry with the two co-formers and have simpler packing motifs than 1 (Fig. 10). In particular, they are characterized by A⋯B⋯A π⋯π stacking prompting to γ-type^67–71^ or β-type^69,71,72^ motifs (see Table S17† for the main interaction distances). As in 1, lateral C–H⋯O interactions are observed in all the studied compounds (Table S17 in the ESI†). In line with 1, the structural motifs of 2 have been compared to the most similar co-crystals in the CSD database (Table S18 in ESI†).^73–79^ All eight co-crystal examples found are in a 1 : 1 stoichiometry and show a γ-type or β-type packing motif. Moreover, C–H⋯N or C–H⋯O lateral stabilizing interactions (see Table S18 in the ESI for the main interaction distances†) can be observed in the compounds. We can notice that all the co-crystals identified as the closest in Euclidean space are characterized by the same main interactions (*i.e.*, γ-stacking and C–H⋯N/C–H⋯O lateral interactions) of 1 and 2.

## Conclusions

We have proposed a general framework for tackling some of the limitations that application of machine learning in materials science is currently facing. Instead of assuming the availability of densely and uniformly sampled data, we focus our attention on identifying the most effective way to handle imbalanced datasets. Given the relative abundance of data available in existing structural databases, the use of machine learning is very attractive to provide effective prediction for properties relating to the solid-state. The drawback here is that the existing databases constructed of published literature typically only include positive results, with scientists very rarely publishing such clear details of experiments that did not work. This means that, from a machine learning perspective, only one class (*i.e.*, the positive outcome) is well defined by the data. Recent research from a range of groups has attempted to tackle this unbalanced data problem for prediction problems like co-crystallization,^11,40^ solvate formation^80,81^ and crystallisability.^82,83^ In general, these groups have attempted to get around the problem by using either sparse or somewhat unreliable negative data from alternative sources to produce a trained model. Our work illustrates that one class classification can overcome these limitations and learn how to effectively describe a certain class of interest, showing the potential to significantly advance many areas of chemical research.

As such, we highlight the implementation of one class classification as a methodology for dealing with the ‘only positive data’ challenge in materials design. We report as a case study the prediction of new molecules which have not previously been recognised as co-formers in the unique and limited class of materials, the π–π interconnected co-crystals. In the attempt to improve our understanding about one class classification, a broad overview about the current methods and concepts is given. The problem is initially investigated using traditional one class classification algorithms in lower dimensions after extensive feature engineering. Further on, we demonstrate that by using a Deep One Class approach, the manual feature engineering could be avoided and we can not only achieve higher accuracy, but also the incorporation of more feature interactions among the co-formers. In this way, all the features that might lead to the formation of stable co-crystals are taken into consideration and the relationships among them are extracted. Co-crystallization emerges as a difficult task for both computational predictions and experimental screening, particularly for cases of limited strong directional forces that could give a strong indication for a successful outcome. In our contribution, we show that the implementation of the appropriate data mining strategy combined with the extraction of a reliable dataset can leverage the synthetic attempts and lead to the successful discovery of new materials. Moreover, an in-depth understanding of the machine learning model with a rationale about the predictions is sought after for advancing our knowledge on the chemical factors that favour co-crystal formation.

Currently, many steps towards explainability of machine learning models have been made. Therefore, for a computational strategy to be reliable it is important to incorporate interpretability for rationalizing the predictions. SHAP calculations were carried out for interpreting the scoring of the deep learning model by assigning feature weights. Consequently, a better understanding of the features that dominate the known molecular pairs is gained and meaningful information regarding the characteristics of the molecules that can relate to π–π stacking is extracted. Shape, size and polarity were detected as important factors for co-crystallization, which is in accordance with previous understanding about the co-crystals of CSD. However, our analysis reveals a more complex scenario, where co-crystallization is feasible for molecules having similar low values of these properties or coupling molecules with low and high values of the same feature. Overall, it can be concluded that the rules that dominate the co-crystal formation are far more complex than just some general properties and many parameters should be taken into consideration.

The computational strategy followed is able to successfully extract the patterns that dominate the known co-crystals and predict a range of potential combinations showing similar trends with the labelled data. Therefore, the number of experiments as well as the time frame required to obtain new compounds can be significantly reduced by focusing on co-formers with high scores and possible interesting properties. A realistic picture of the single class applicability is demonstrated by the identification of two molecules that have not previously been recognized as co-formers. The co-formers of 1 and 2 are characterized by similar shape/size, polarity and electronic characteristics, confirming the ability of the model to learn and reproduce the key-features of the labelled dataset. Interestingly, 1 and 2 crystalize in the rare γ-packing type which represents only the 8% among pyrene co-crystals, pointing out the power of our model in exploring, understanding and expanding the targeted labelled dataset. Overall, using the proposed machine learning strategy we were able to successfully overcome the limitations of an ‘only positive example’ problem in the π–π interconnected co-crystals dataset with the identification and experimental realization of two co-crystals (pyrene-6*H*-benzo[*c*]chromen-6-one (1) and pyrene-9,10-dicyanoanthracene (2)), both containing molecules which have not previously been reported as co-formers in the CSD.

## Conflicts of interest

There are no conflicts to declare.

## Supplementary Material

SC-012-D0SC04263C-s001

SC-012-D0SC04263C-s002
