# Supplementary material for: One class classification as a practical approach for accelerating π–π co-crystal discovery
Source: Chem Sci. 2020 Dec 8;12(5):1702–19. doi: 10.1039/d0sc04263c (PMC8179233; doi:10.1039/d0sc04263c)
Supplement: SC-012-D0SC04263C-s001 [file SC-012-D0SC04263C-s001.pdf]

Electronic Supplementary Material (ESI) for Chemical Science.
This journal is © The Royal Society of Chemistry 2020

Supporting Information

One class classification as a practical approach for accelerating π-π co-crystal discovery

Aikaterini Vriza,[a,b] Angelos B. Canaj,[a] Rebecca Vismara,[a] Laurence J. Kershaw Cook,[a] Troy D. Manning,[a] Michael W. Gaultois,[a,b] Peter A. Wood,[c] Vitaliy Kurlin,[d] Neil Berry,[a] Matthew S. Dyer*[a,b] and Matthew J. Rosseinsky[a,b]

[a] Department of Chemistry and Materials Innovation Factory, University of Liverpool, 51 Oxford Street, Liverpool L7 3NY, UK.

[b] Leverhulme Research Centre for Functional Materials Design, University of Liverpool, Oxford Street, Liverpool L7 3NY, UK.

[c] Cambridge Crystallographic Data Centre, 12 Union Road, Cambridge CB2 1EZ, UK.

[d] Materials Innovation Factory and Computer Science department, University of Liverpool, Liverpool, L69 3BX UK.

Supplementary Material

Table of Contents

	Glossary of technical terms	S3
1.	Generating the datasets:	S4
	1.1 Co-crystals extraction from Cambridge Structural Database (CSD)	
	1.2 Designing the unlabelled (ZINC15) dataset	
	1.2.1 Filtering with Pipeline Pilot	
2.	One Class Classification Review	S8
	2.1 Standard One Class Classification Algorithms	
	2.1.1 Feature Engineering	
	2.2 Deep One Class (SetTransformer-DeepSVDD)	
	2.3 Evaluation and comparison	
3.	Visualizing the predicted pairs	S20
4.	Predicting Molecular Stoichiometry	S28
5.	Interpretability	S30
	5.1. Shapley values for the co-crystal dataset	
	5.2. Shapley values for the pyrene co-crystals	
	5.3. Important correlations between descriptors	
	5.4. Descriptor distributions	
6.	Experimental Realization (Pareto Optimization)	S43
7.	Experimental Section	S48
8.	Comparison with known structures	S52
	8.1 Pyrene-based co-crystals	
	8.2. UMAP projection of the co-crystals space	
	8.3. Euclidean Distance Calculation	
SI References		S62

Glossary of technical terms

Labelled dataset: The known co-crystal combinations that were extracted from Cambridge Structural Database (CSD)

Unlabelled Dataset: The dataset of possible molecular combinations that was designed from ZINC15 Database

Two dimensional descriptors: Descriptors calculated from the two-dimensional representation of a molecule (molecular graph)

Bidirectional Dataset: A dataset constructed by concatenating the descriptor vectors in both directions (a,b) and (b,a)

ECFP4: Extended Connectivity Fingerprint

1. Generating the datasets

1.1 Cocrystal extraction from Cambridge Structural Database (CSD)

Starting from eight representative polyaromatic hydrocarbons (PAHs) we extracted all the co-crystals that include as a co-former either these or their structurally similar molecules. The structural similarity was measured with Tanimoto similarity (> 0.35). The multi-component crystal structures that contain solvent molecules were removed, keeping only the benzene like solvents, as they might hold information about π-π interactions. The solvents list implemented was the default CCDC most common solvent list.

Table S1. Initial Polyaromatic Hydrocarbons (PAHs) for co-crystals extraction.

CCDC Search Identifier	Zinc Search Identifier	Actual Name	Molecular structure
CORONE	ZINC0000001580987	CORONENE	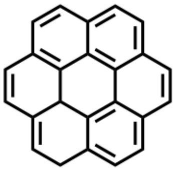
ZZZOYC04	ZINC000001598876	PICENE	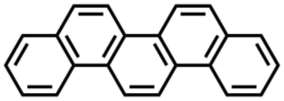
PENCEN	ZINC000001581013	PENTACENE	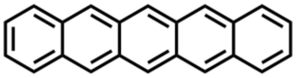
TRIPHE	ZINC000001688068	TRIPHENYLENE	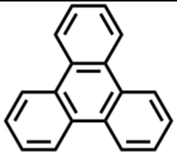
PHENAN	ZINC000000967819	PHENANTHRENE	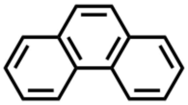
FLUANT	ZINC000008585874	FLUORANTHENE	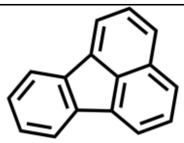
CORANN01	ZINC0000079045456	CORANNULENE	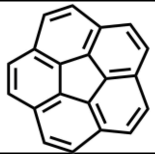
DNAPAN	ZINC0000167079286	DINAPHTHO,(1,2 a:1',2'-h) ANTHRACENE	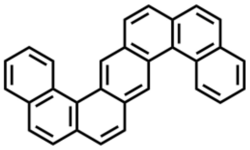

Searching the Cambridge Structural Database:

For the extraction of the PAH co-crystals, the Python API functionality of CCDC was employed. The two main search functions used are the similarity and substructure search.[1] The similarity search is based on the comparison of molecular fingerprints and works as following: given a query molecule, in our case the molecules were given as SMILES strings, a 2D structure-based search is performed to determine molecular components that are similar to the input. For each separate molecule in a crystal structure a molecular fingerprint of 2040 bits is generated, using all atom and bond paths up to ten atoms in a molecule.[1] That search reveals not only single molecules but also combinations of molecules, potentially because of the large fingerprint space used.

The similarity search function of the CSD Python API was applied to the starting PAHs, using the standard CSD fingerprint similarity search with a Tanimoto similarity threshold of > 0.35. The extracted structures were then filtered by removing duplicate structures (polymorphs), as there are several polymorphs for some co-crystals but as our machine learning workflow is based on the two-dimensional descriptors we only considered the two different types of molecules that exist in a structure and not the packing. The INCHI number of each molecule was implemented for the filtering as INCHI numbers are more unique whilst two different SMILES might represent the same molecule. After removing the duplicates, the extracted molecules were split into categories based on the number of times the molecules in the pair appear. In that way we can measure the molecular stoichiometry. For the category including only single components the substructure search was further applied to detect any potential combinations that were not found from the similarity search. The same filters were applied as for the similarity search.

A substructure search was implemented to search for structures containing a required component, which was in our case the co-crystals containing at least one of the starting PAHs or any molecule similar to them as found from the similarity search.

After obtaining the final co-crystals dataset the structures that include common solvents are removed, except from those containing benzene-like solvents that might hold important information about π-π interactions.

The percentage of the extracted PAH co-crystals connected with π-π stacking out of the whole co-crystals dataset was measured after calculating the number of existing co-crystals in the CSD database. The whole CSD was searched for structures containing two different molecules using the same search settings as for the extraction of PAHs co-crystals:

settings.only_organic = True

settings.not_polymeric = True

settings.has_3d_coordinates = True

settings.no_disorder = True

settings.no_errors = True

settings.no_ions = True

settings.no_metals = True

We identified 13,817 co-crystals including co-crystals containing benzene-like solvents (solvates), meaning that the 1,722 PAH co-crystals connected with π-π stacking compose the 12% of the total.

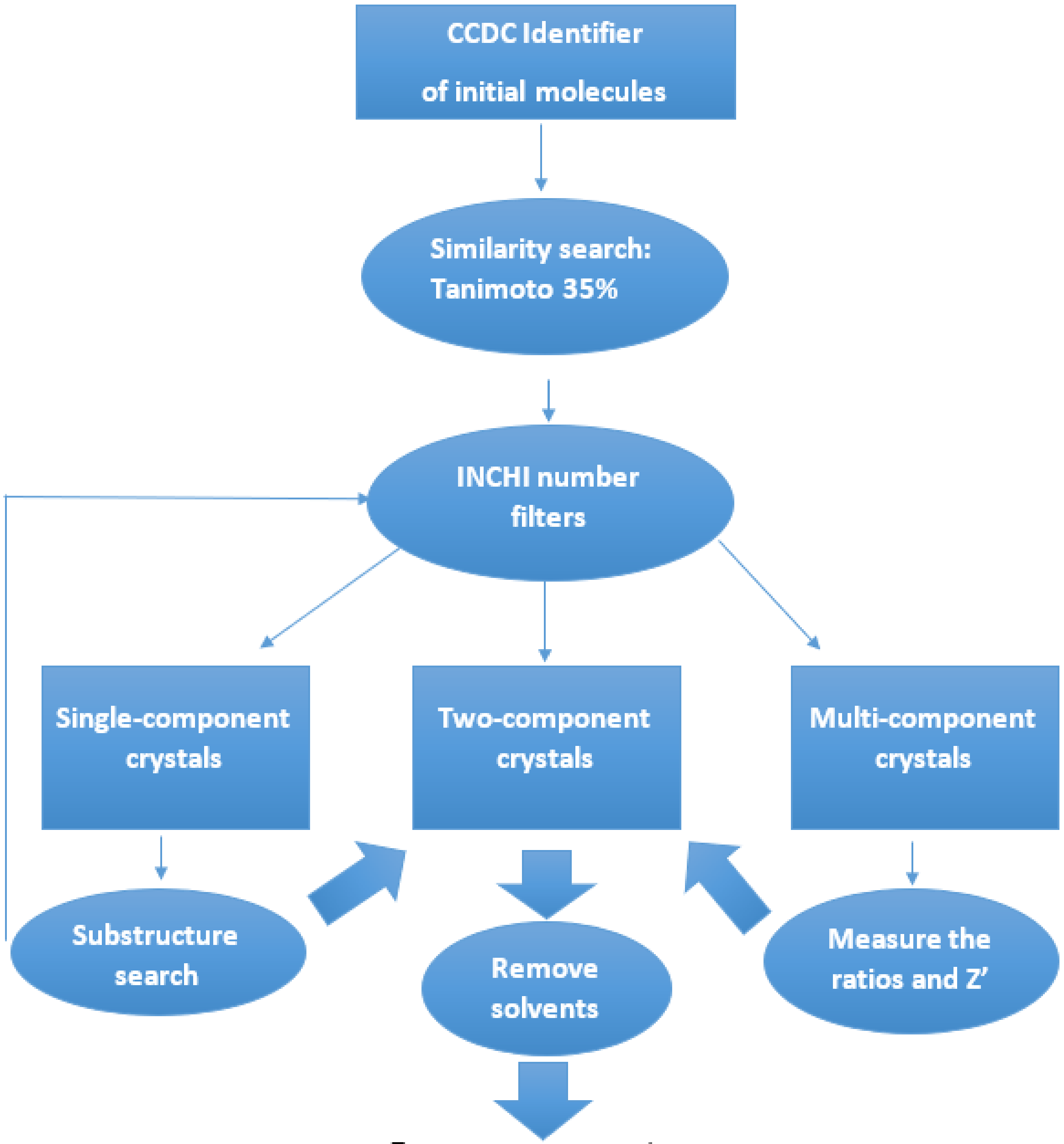

Two-component co-crystals

CCDC Identifier	First_molecule	Second_molecule	Z'	Ratio
YANGIF	C1N2CN3CN1CN(C2)C3	IN1C(=O)CCC1=O	3	1:2
…..				
…				

Figure S1. Flow diagram for PAH co-crystals extraction. The search starts with 8 representative PAHs and Python API CCDC is employed for extracting all the co-crystals that are formed from these 8 molecules or molecules that are similar to them on the basis of molecular fingerprints (ECFP4 > 0.35 Tanimoto Similarity). The extracted dataset was further filtered for removing co-crystals containing molecules with acidic parts.

1.2 Designing the unlabelled (ZINC15) Dataset

A search of the ZINC15 database for molecules similar to the eight initial molecules of Table 1 on the basis of molecular fingerprints with a Tanimoto similarity threshold of > 0.35, which are purchasable and do not contain incompatible functional groups, afforded a library of 210 candidate molecules. All the possible order invariant pairwise combinations of these candidates compose the unlabelled dataset. Similarity search in ZINC15 is based on 512 bit ECFP4 fingerprints[2], meaning that the atomic environment between two under comparison molecules is four bonds length with size of fingerprint is 512 bits. It is well discussed that different libraries present significant structural variations and thus the ECFP features can have quite different values[3]. The small overlap between Zinc and CSD databases can be explained in that way, especially if we consider how CSD database performs the similarity search.

1.2.1 Filtering with Pipeline Pilot

The filtering for incompatible functional groups in both the labelled and unlabelled dataset was performed using Pipeline Pilot[4] with the following workflow.

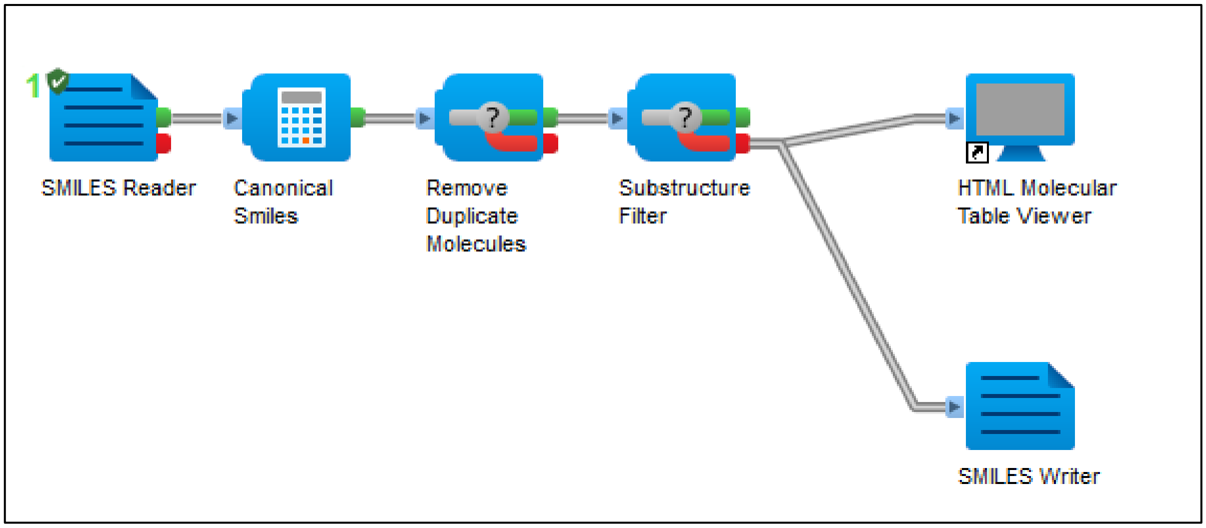

Figure S2. Pipeline Pilot workflow.

Substructure Smarts Filter

```
[$([OH]-*=[!#6])]
[NX3;H2,H1]
[OX2H]
[CX3H1](=O)[#6]
[SX2H]
[nH]
[CX4][F,Cl,Br,I]
[#6]1[O][#6]1
```

Figure S3. Substructure SMARTS[5] filter for detecting the molecular combinations with at least one molecule with acidic hydrogens.

2. One class classification Review

Distribution based. Methods in this category are basically inspired from statistical modelling. They deploy some standard distribution model (*e.g.*, normal distribution) and flag as outliers the instances that deviate from the model, whereas inliers are those that follow the same distribution.[6] Typical examples are the Autoencoders and the Gaussian Mixture models.

Density based. These methods assume that normal data points occur around a dense neighbourhood. The local outlier factor (LOF) approach is one of the well-known algorithms in this category, where normal points get low LOF values as they belong to a local dense neighbourhood. The density of a neighbourhood is estimated using the distance to the *k* nearest neighbours, with *k* being the minimum number of neighbours used for defining the local neighborhood.[7]

Distance based. Among other distance based methodologies, k-nearest neighbour algorithm ranks each point on the basis of its distance to its kth nearest neighbor.[2,7] The lower the distance the closer to the normal data is the point.

Clustering based. Clustering Based Local Outlier Factor (CBLOF) is an algorithm developed for considering both the size of clusters and the distance between points and the closest cluster. Each datapoint is then assigned a score/outlier factor based on these considerations.[9]

Support Vector Machine. One class support vector machine algorithm (OCSVM) is an extension on the well-known support vector machine technique. The planar approach of OCSVM is about finding a linear boundary to maximally separate all the data points from the origin, whereas the spherical approach designs a spherical boundary in feature space around the data (the hypersphere) and the algorithm tries to minimize the volume of the hypersphere.[10]

Histogram-based. For each single feature, a univariate histogram is constructed where the height of the bins gives an estimation of the density. Then, the score of each point is calculated by combining all the histograms using the corresponding height of the bins where the point is located.[11]

Forest-based. Whilst most of the aforementioned models are essentially used to profile the normal labelled data, this model is focused on isolating anomalous instances. The isolation forest algorithm is recursively randomly partitioning a randomly selected feature between its minimum and maximum values. The number of recursive partitions, represented as a tree structure, required to isolate an instance is equivalent to the path length from the root node to the terminating node. The instances with short path lengths are regarded as anomalies with the anomaly score being computed by the mean anomaly score of the trees in the forest.[12]

Ensemble-based. The ensemble technique involves a number of base detectors being fitted to different sets of features of the dataset and the outliers are identified based on the probability of each point being an anomaly. Representative model of this category is the feature bagging algorithm.[13]

Deep One Class. In contrast to traditional approaches which make use of heuristics or statistical methods, deep learning approaches stack multiple processing layers one above another with each layer providing higher order interactions among the features. Deep learning approaches specifically designed for one class classification are not yet very widespread. The majority of the existing models involve neural networks being trained to perform tasks other than one class classification which are then adapted for use in the one class problems. Deep networks designed for one class (anomaly detection) involve the objective function of a traditional one class approach. However, they are trained deeper *i.e.,* using more layers and in higher dimensions for fitting the appropriate function to the normal data. Deep learning models could easily handle more complex molecular representations as inputs, *e.g.*, SMILES strings or 3D molecular configurations.[14]

All the aforementioned algorithms were tested for solving the co-crystal prediction problem. It should be highlighted that there is no single 'best' method for dealing with one class classification tasks. The appropriateness of each algorithm is highly associated with the problem to be solved and the available dataset.

2.1 Standard One Class Classification Algorithms

2.1.1 Feature Engineering

Feature engineering based on correlations between the Dragon descriptors of the conformers of the known dataset. The most important descriptors were found to be the following:

Table S2. Pairwise correlations of the most important Dragon descriptors.

Dragon Descriptor	Description	Pearson Correlation	Spearman Correlation	p-value
nBT	molecular weight	0.403	0.620	$< 10^{-5}$
nHet	number of heteroatoms	0.515	0.685	
ZM1V	first Zagreb index by valence vertex degrees	0.528	0.729	
DBI	Dragon branching index	0.548	0.654	
ICR	radial centric information index	0.546	0.422	
MAXDN	maximal electrotopological negative variation	0.440	0.600	
MAXDP	maximal electrotopological positive variation	0.426	0.626	
DELS	molecular electrotopological variation	0.414	0.629	
CIC0	Complementary Information Content index (neighborhood symmetry of 0-order)	0.298	0.515	
J_D/Dt	Balaban-like index from distance/detour matrix	0.323	0.424	
SM1_Dz(Z)	spectral moment of order 1 from Barysz matrix weighted by atomic number	0.551	0.627	
SM1_Dz(v)	spectral moment of order 1 from Barysz matrix weighted by van der Waals volume	0.404	0.479	
SM1_Dz(e)	spectral moment of order 1 from Barysz matrix weighted by Sanderson electronegativity	0.480	0.558	
HyWi_B(s)	hyper-Wiener-like index (log function) from Burden matrix weighted by I-State	0.744	0.682	
SpMax4_Bh(m)	largest eigenvalue n. 4 of Burden matrix weighted by mass	0.541	0.571	
SpMax3_Bh(s)	largest eigenvalue n. 3 of Burden matrix weighted by I-state	0.422	0.482	
SpMax7_Bh(s)	largest eigenvalue n. 7 of Burden matrix weighted by I-state	0.439	0.542	
P_VSA_v_2	P_VSA-like on van der Waals volume, bin 2	0.501	0.684	
P_VSA_s_6	P_VSA-like on I-state, bin 6	0.522	0.704	
Eta_F_A	eta average functionality index	0.434	0.438	
Eig02_AEA(dm)	eigenvalue n. 2 from augmented edge adjacency mat. weighted by dipole moment	0.530	0.539	
Eig03_AEA(dm)	eigenvalue n. 3 from augmented edge adjacency mat. weighted by dipole moment	0.609	0.572	
nHAcc	number of acceptor atoms for H-bonds (N,O,F)	0.449	0.620	
Uc	unsaturation count	0.520	0.551	

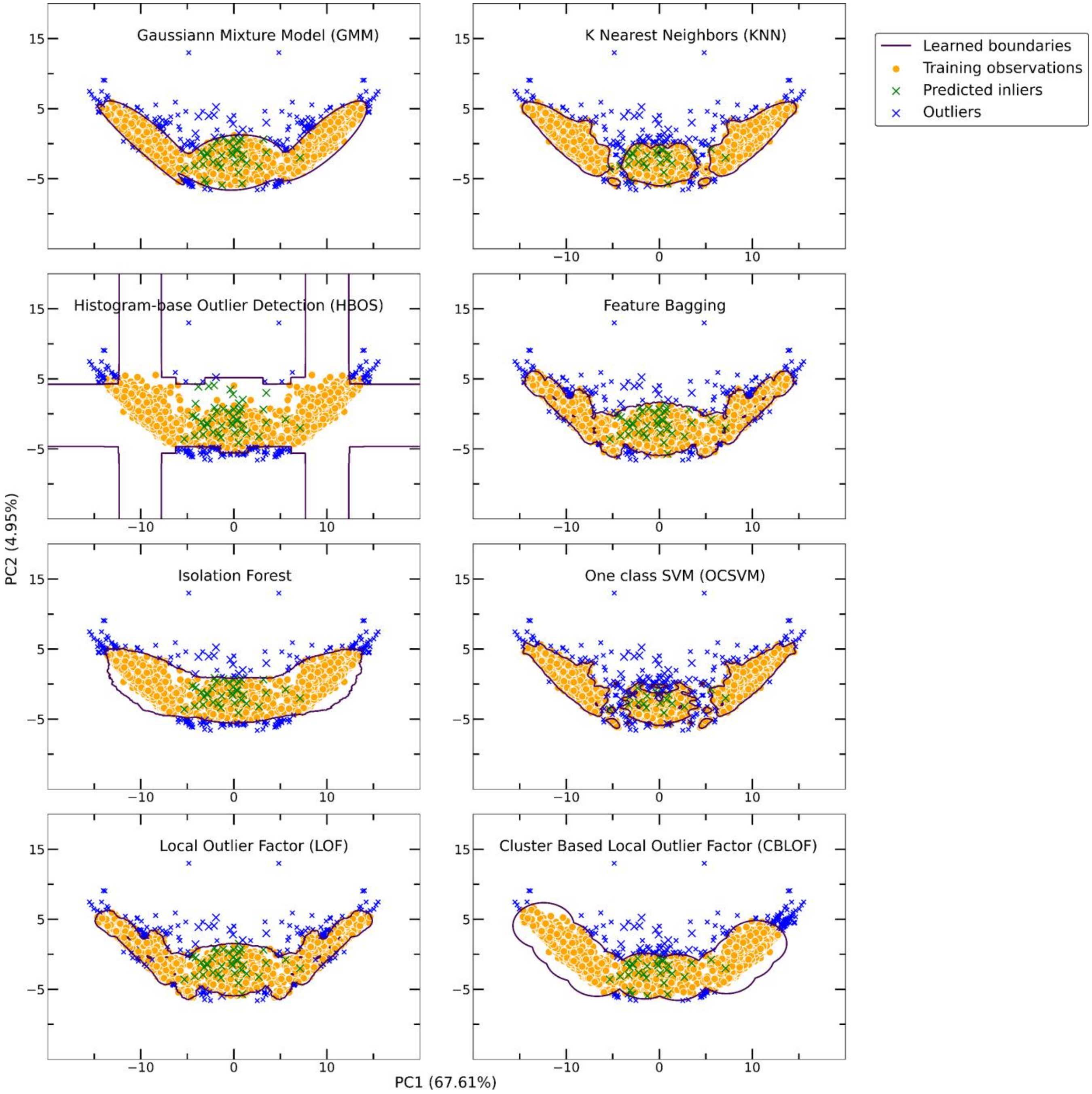

Figure S4. A demonstration of the effect the implemented one class classification/anomaly detection algorithms have on the initial dataset when projected in two-dimensions. Principal Component Analysis (PCA) was employed are the dimensionality reduction techinique. The expained variance is 67.61% for the first Principal Component and 4.95% for the second. All the dimensions are implemented (3700). The outliers found each time either belong to to labeled dataset as noise or to the unlebeled dataset as the outling part.

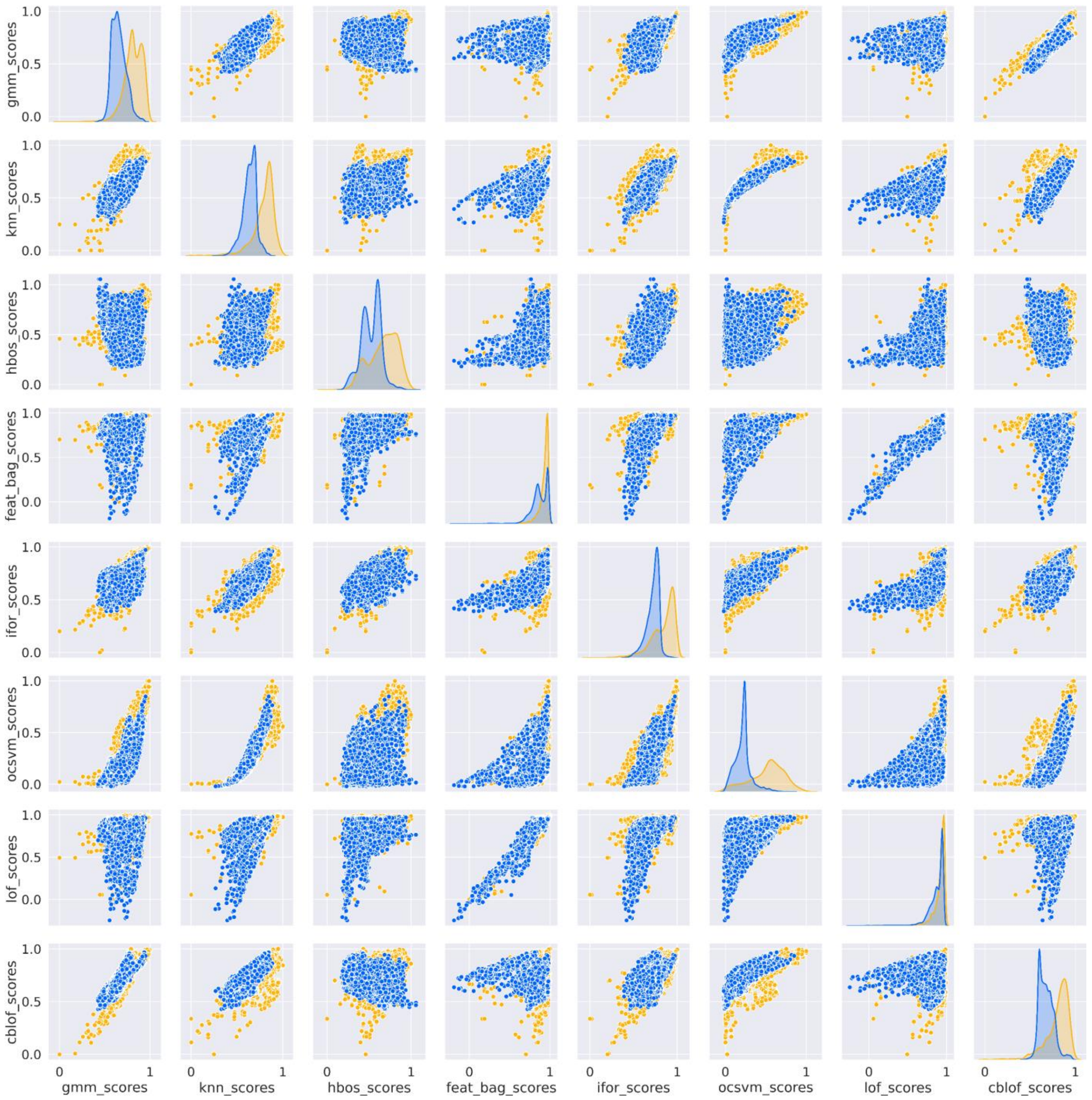

Figure S5. Pairwise correlations of the scores of the known (yellow) and unknown (green) data using standard one-class classification algorithms. Each algorithm uses a different scoring function to assign scores to the molecular combinations, giving in all the cases higher scores to the known (training set) whereas only a certain part of the unknown combinations (test set) is getting high scores and can be regarded as inliers.

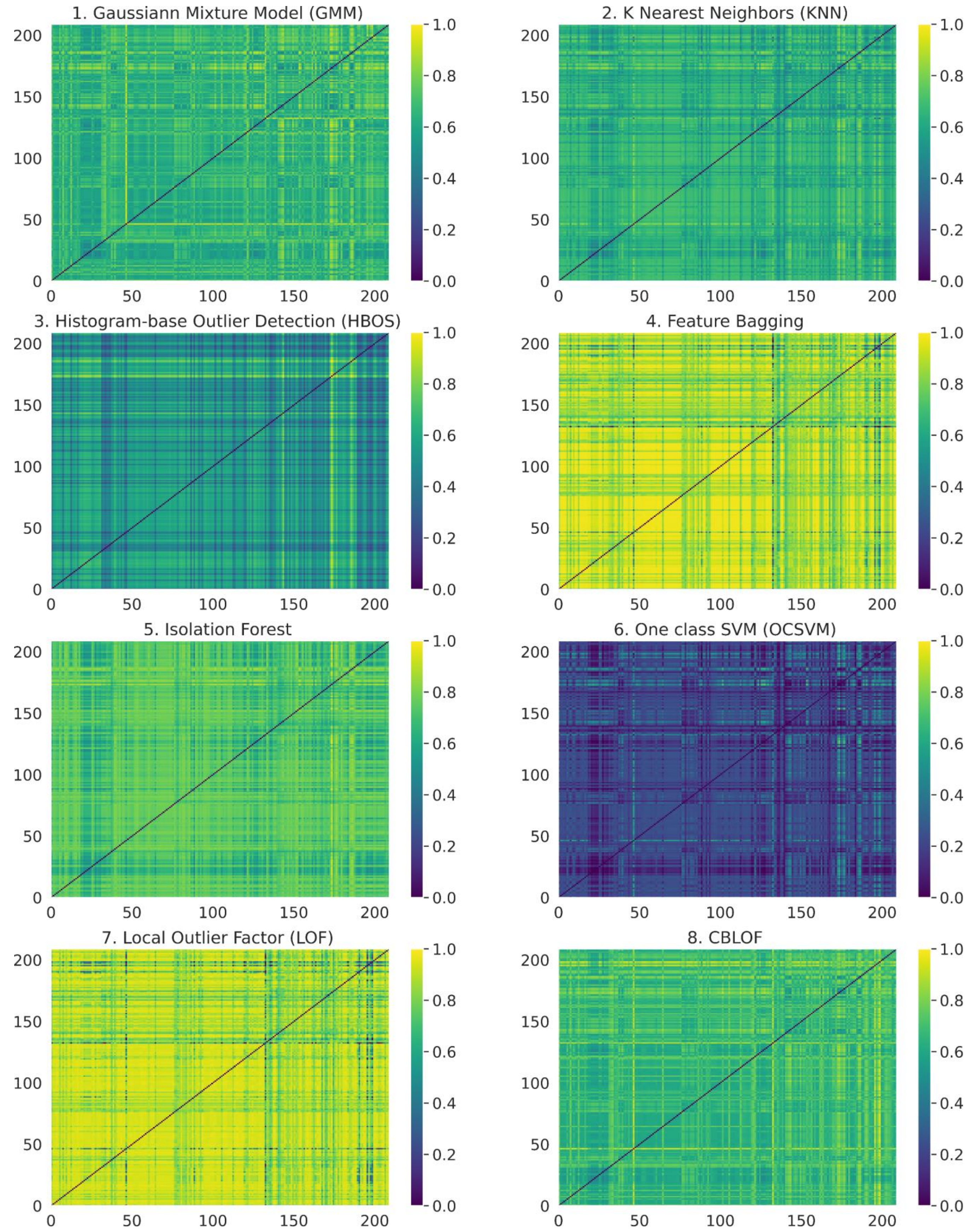

Figure S6: Heat-maps with the scoring of each algorithm on the unlabelled dataset.

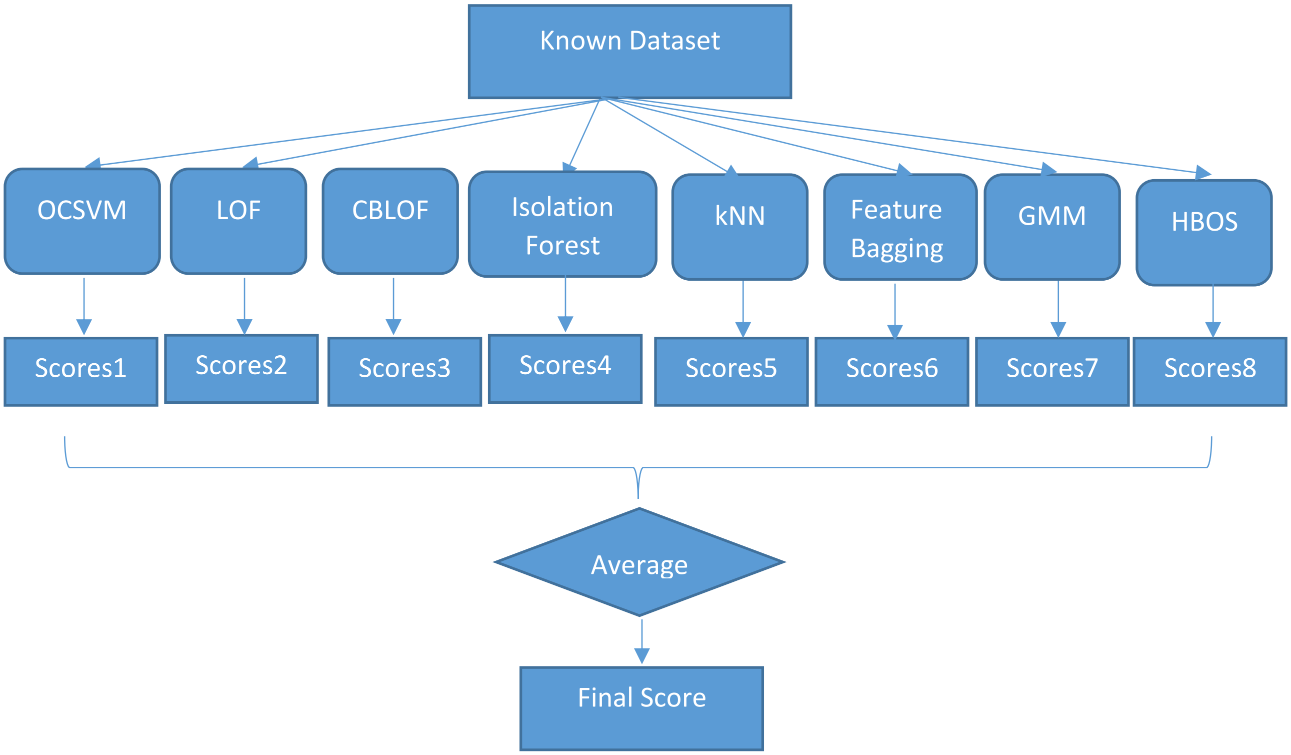

Figure S7. Illustration of ensemble learning technique used for combining the scores of each of the standard models.

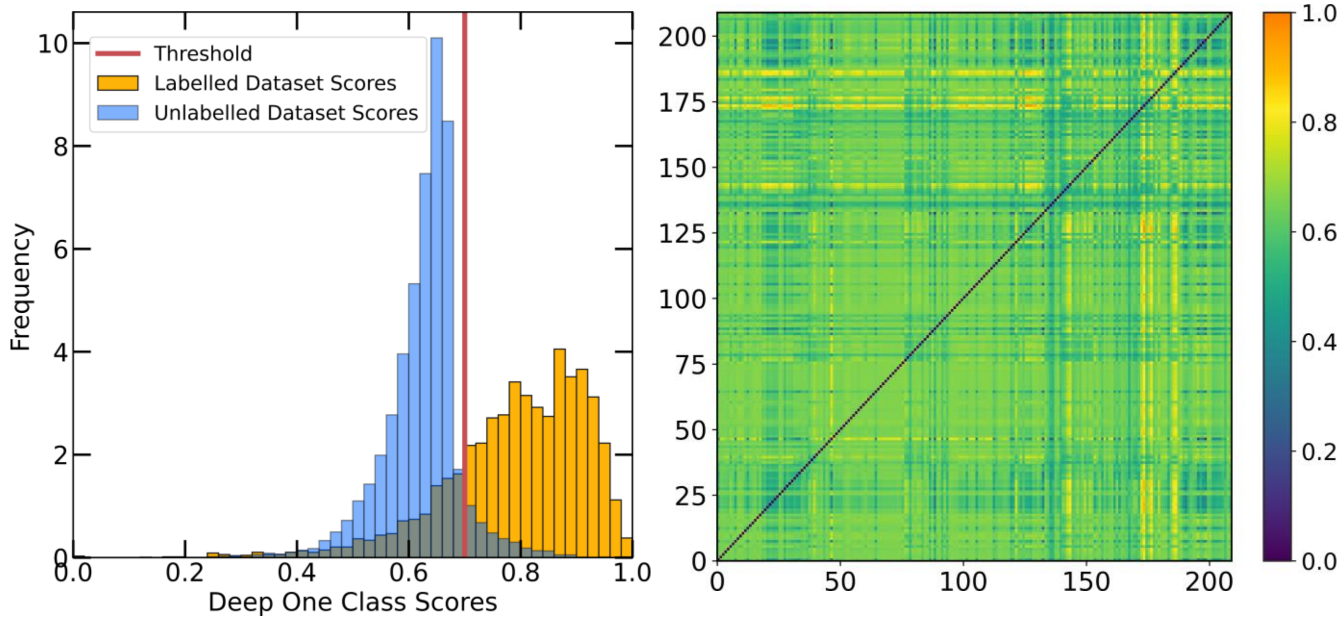

Figure S8. Labelled/Unlabelled scores distribution and test scoring matrix using the ensemble of one-class classification algorithms.

2.2 Deep One Class (SetTransformer-DeepSVDD)

The neural network architecture is adapted from Ruff *et al*,[15] namely DeepSVDD. The convolutional autoencoder used on DeepSVDD network was replaced with an attention based autoencoder which is permutation invariant, namely SetTransformer. The architecture of SetTransformer was adapted from Lee *et al*[16] and was used for learning the representation of the molecular pair such that they will be perceived as order invariant vectors. SetTransformer includes two stacked SABs (Set Attention Block) and one PMA (Pooling by Multihead Attention) layers in the encoder followed by two linear decoder layers. The first part of the encoder independently acts on each element of the vector (SAB) and then on the second part the encoded features are aggregated to produce the desired output. The decoder part is only used for the initialization of the weights and then is not further employed in the training. The loss function of DeepSVDD is referred to the minimization of the volume of a hypersphere that includes the normal data. In our case as normal data we regarded all the known co-crystals extracted from CCDC. The hyperparameters of the network (number of epochs and learning rate) were selected based on k-fold cross validation such that after minimizing the volume of the hypersphere significantly, the majority of the datapoints of the hold-out test are found in the hypersphere

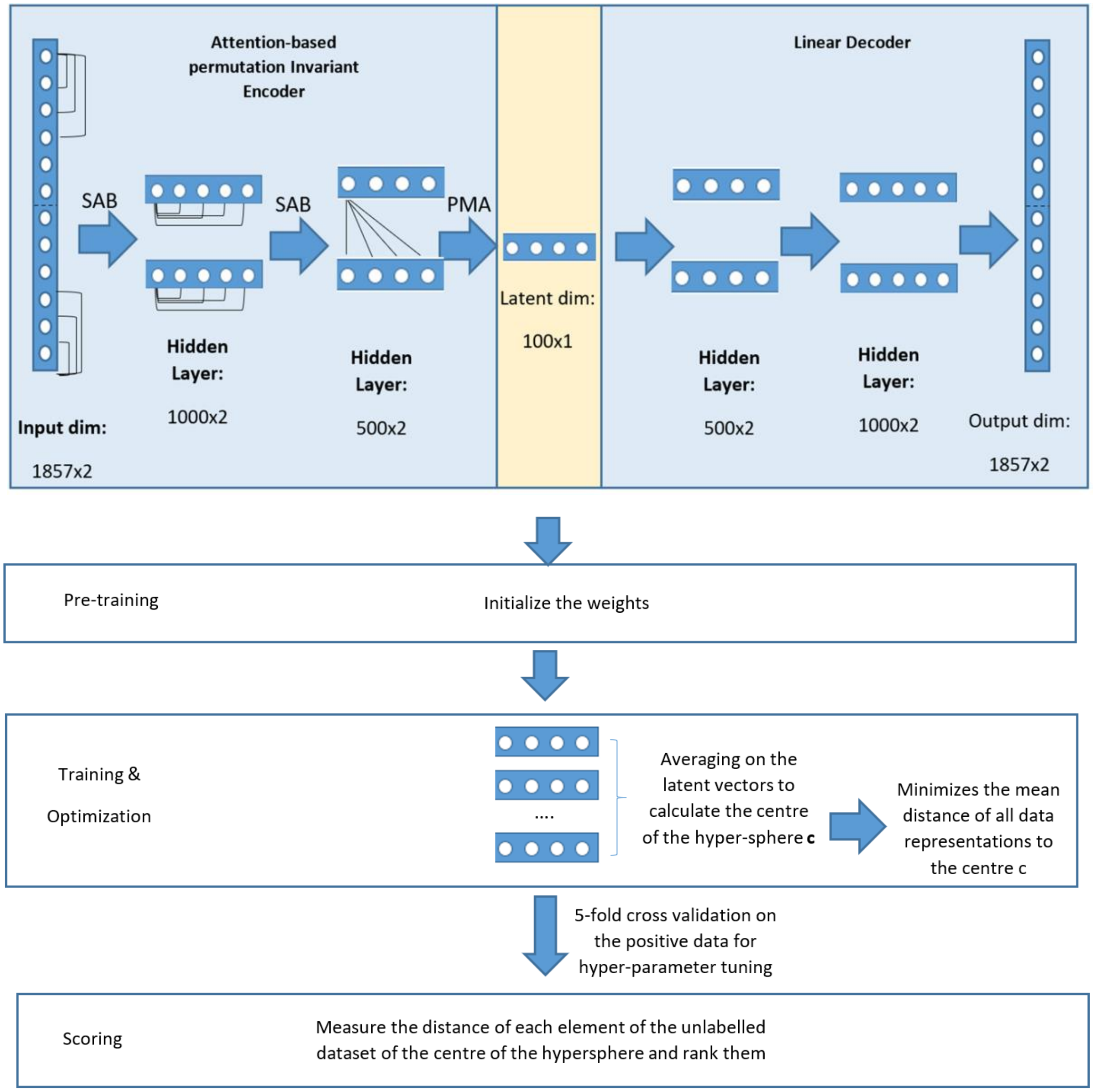

Figure S9. Neural Network Architecture.

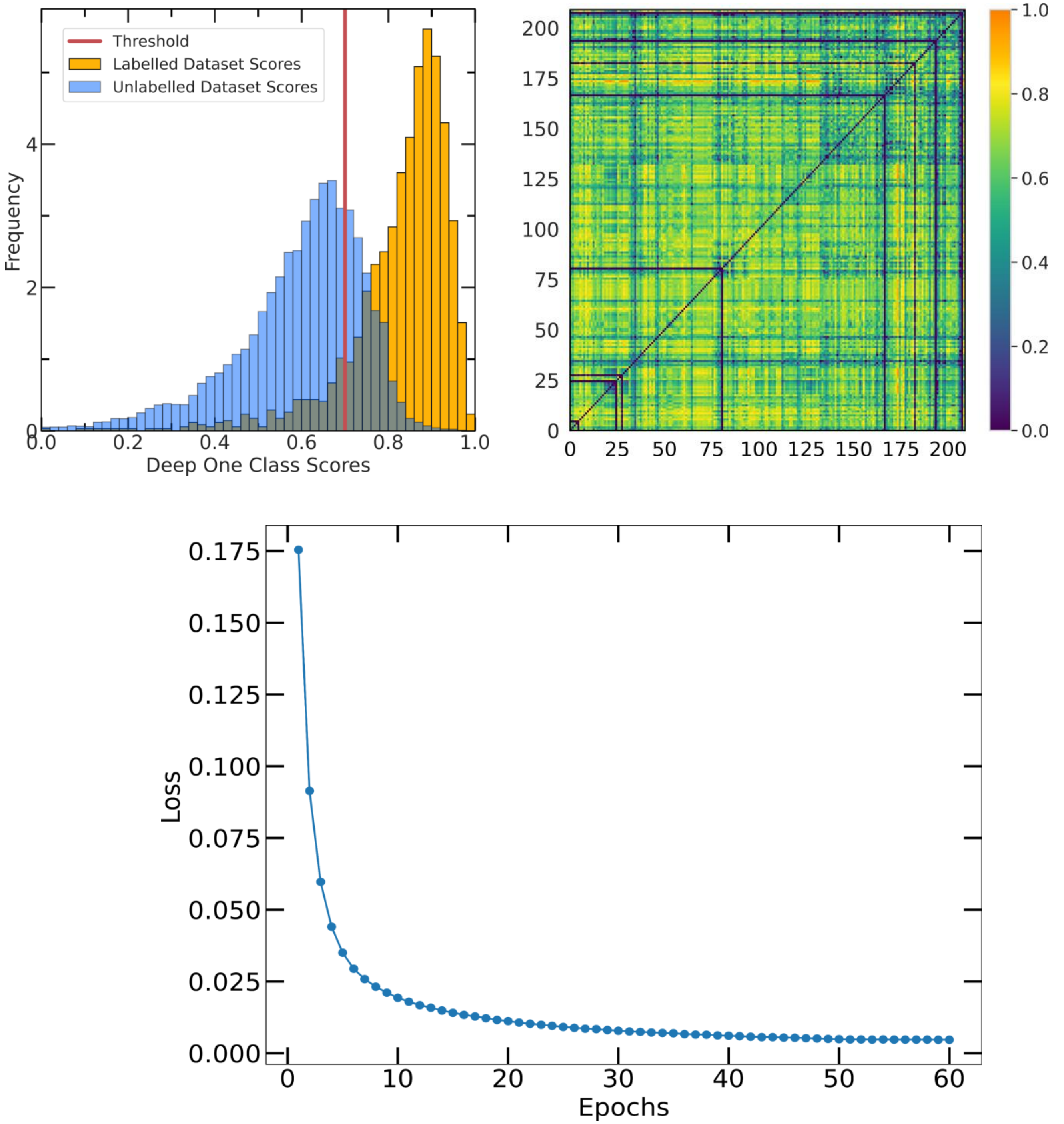

Figure S10. a) Labelled/Unlabelled scores distribution and test scoring matrix using the ensemble of one-class classification algorithms. b) training loss after 60 epochs implementing DeepSVDD network.

The reproducibility of the model was checked after performing the pretraining and training steps for 30 times with a varying number of seeds keeping 10% as a validation set each time. The mean Pearson correlation of the predicted scores was 0.96 with mean standard deviation 0.0017 That is an indication that there is high reproducibility of the results and thus for being able acquire the same results each time the seed was set to 0.

2.3 Evaluation and Comparison of the models

Table S3. Short description and hyperparameter settings of the models used for one-class classification. Bayesian optimization was implemented to determine the best performance of a single model.

<table>
<tr><th>Model</th><th>Model Description</th><th>Tuned hyperparameters</th><th>Explanation</th></tr>
<tr><td rowspan="3">CBLOF</td><td rowspan="3">Cluster-based algorithm. Calculates the distance between points and the closest cluster.</td><td>alpha=0.9</td><td>Ratio of the number of samples in large clusters to the number of samples in small clusters.</td></tr>
<tr><td>beta=4</td><td>Coefficient for cluster size</td></tr>
<tr><td>n_clusters=10</td><td>Number of clusters</td></tr>
<tr><td rowspan="2">kNN</td><td rowspan="2">Measures the distance of each observation to its k-nearest neighbour</td><td>n_neighbors=17</td><td>Number of neighbours</td></tr>
<tr><td>method=’mean’</td><td>The average of all k neighbours is used as the outlier score</td></tr>
<tr><td rowspan="2">HBOS</td><td rowspan="2">Calculates the density of an area based on the height of the constructed histogams</td><td>n_bins=15</td><td>Number of bins</td></tr>
<tr><td>alpha=0.7</td><td>Regularizer for preventing overflow</td></tr>
<tr><td>Feature Bagging (LOF is set by default as the basis algorithm)</td><td>Selects a subset of features which induce diversity to a base detector</td><td>n_neighbors=8</td><td>Number of neighbours</td></tr>
<tr><td>Iforest</td><td>Builds an ensemble of random trees for a given dataset and calculates the average path length</td><td>n_estimators=400</td><td>Number of estimators</td></tr>
<tr><td rowspan="2">OCSVM</td><td rowspan="2">Estimates the support vector of the known distribution</td><td>kernel=’rbf’</td><td>Kernel type to be used</td></tr>
<tr><td>nu=0.08</td><td>Regularization parameter</td></tr>
<tr><td>LOF</td><td>Measures the local deviation of density of a given sample with respect to its neighbours</td><td>n_neighbors=10</td><td>Number of neighbours</td></tr>
<tr><td rowspan="2">GMM</td><td rowspan="2">Attempts to find a mixture of multi-dimensional Gaussian probability distributions that best model the input dataset.</td><td>n_components=6</td><td>Number of components</td></tr>
<tr><td>covariance_type='spherical’</td><td>each component has its own general covariance matrix</td></tr>
<tr><td rowspan="3">DeepSVDD</td><td rowspan="3">Considers that all known points belong to a hypersphere, the volume of which should be minimized</td><td>Batch_size=200</td><td>Batch size of input</td></tr>
<tr><td>Num_epochs=60</td><td>Number of epochs: a single pass through all the training data</td></tr>
<tr><td>lr=10^{-5}</td><td>Learning rate</td></tr>
</table>

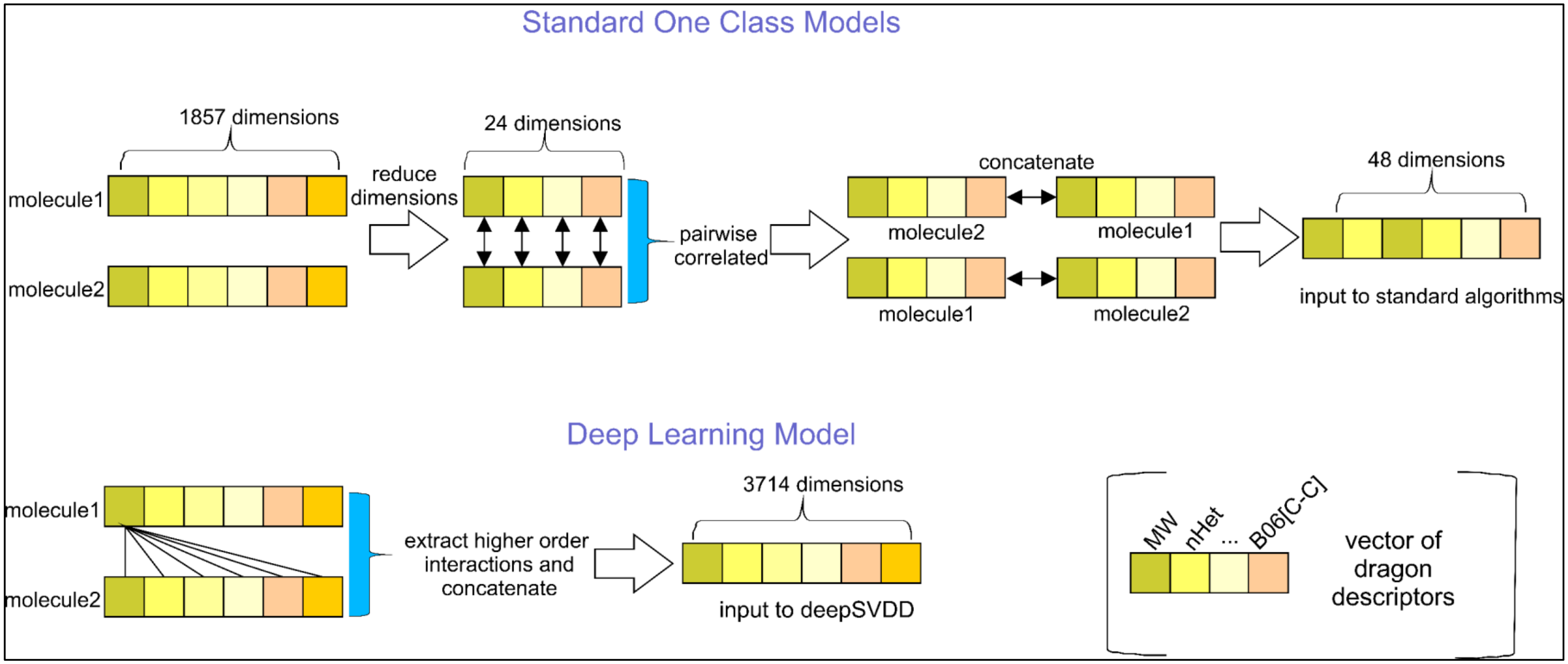

Figure S11. Illustration of the input on the algorithms.

As there are no negative data (known outliers) for performing the typical evaluation steps, which would be based on measuring the AUC or APR, we measure the performance both of the neural network and the traditional classifiers based on the True Positive Rate (TPR%), *i.e.*, the percentage of correctly classified positive data. In more detail, the labelled dataset was split in five-fold using k-fold cross validation, with four-folds being used for the training and one-fold (hold-out data) for the validation. As we assume that 95% of the labelled data are normal with a 5% of noise, a threshold is set as the score above which 95% of the labelled data is scored. Both the k-fold cross validation and the response of the models in the increasing amount of data are investigated for deciding the best model.

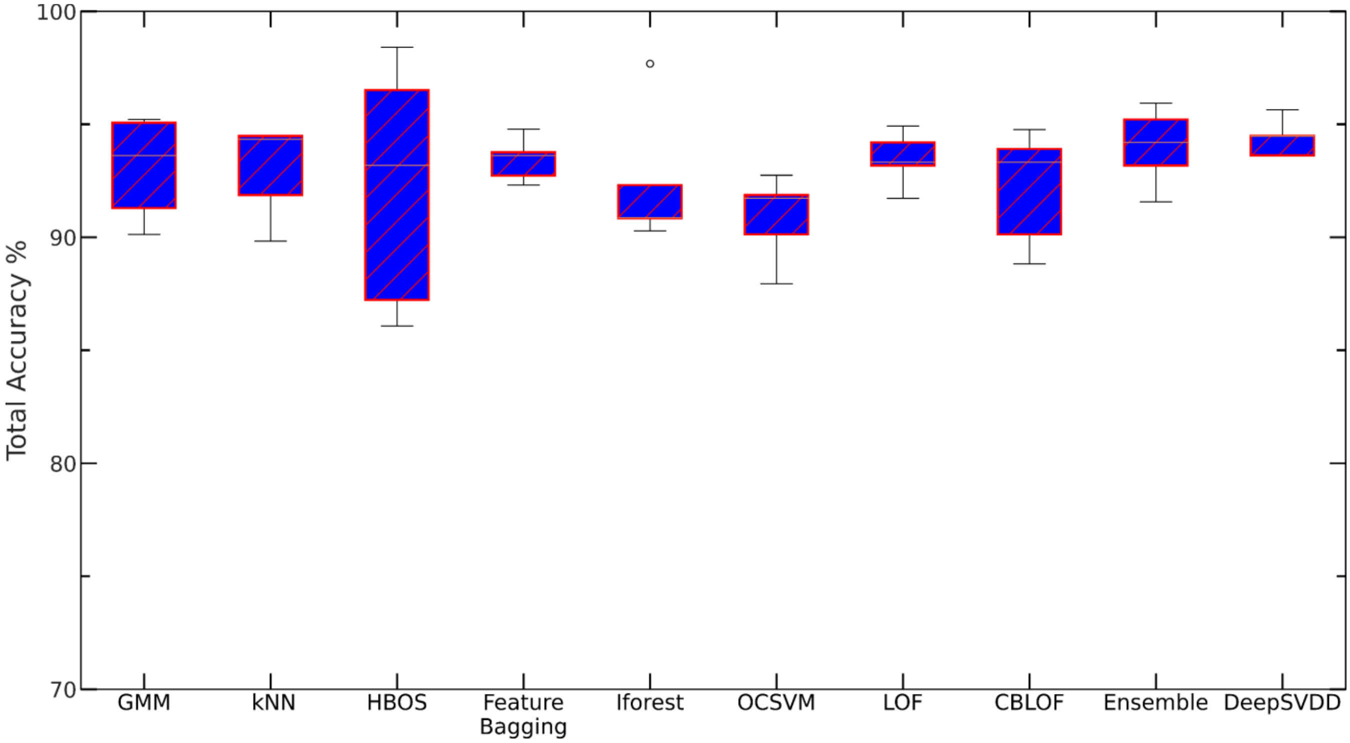

Figure S12. Box plots showing the accuracy of the models after k-fold cross-validation using five folds.

Table S4. Evaluation metrics for the implemented models.

Model	Accuracy (TPR%)	Deviation (%)
**GMM**	93.05	2.03
**kNN**	93.00	1.87
**HBOS**	92.27	4.90
**Feat_Bagging**	93.43	0.85
**Iforest**	92.21	2.31
**OCSVM**	90.88	1.69
**LOF**	93.46	1.07
**CBLOF**	91.52	2.99
**Ensemble**	94.01	1.53
**DeepSVDD**	94.36	0.74

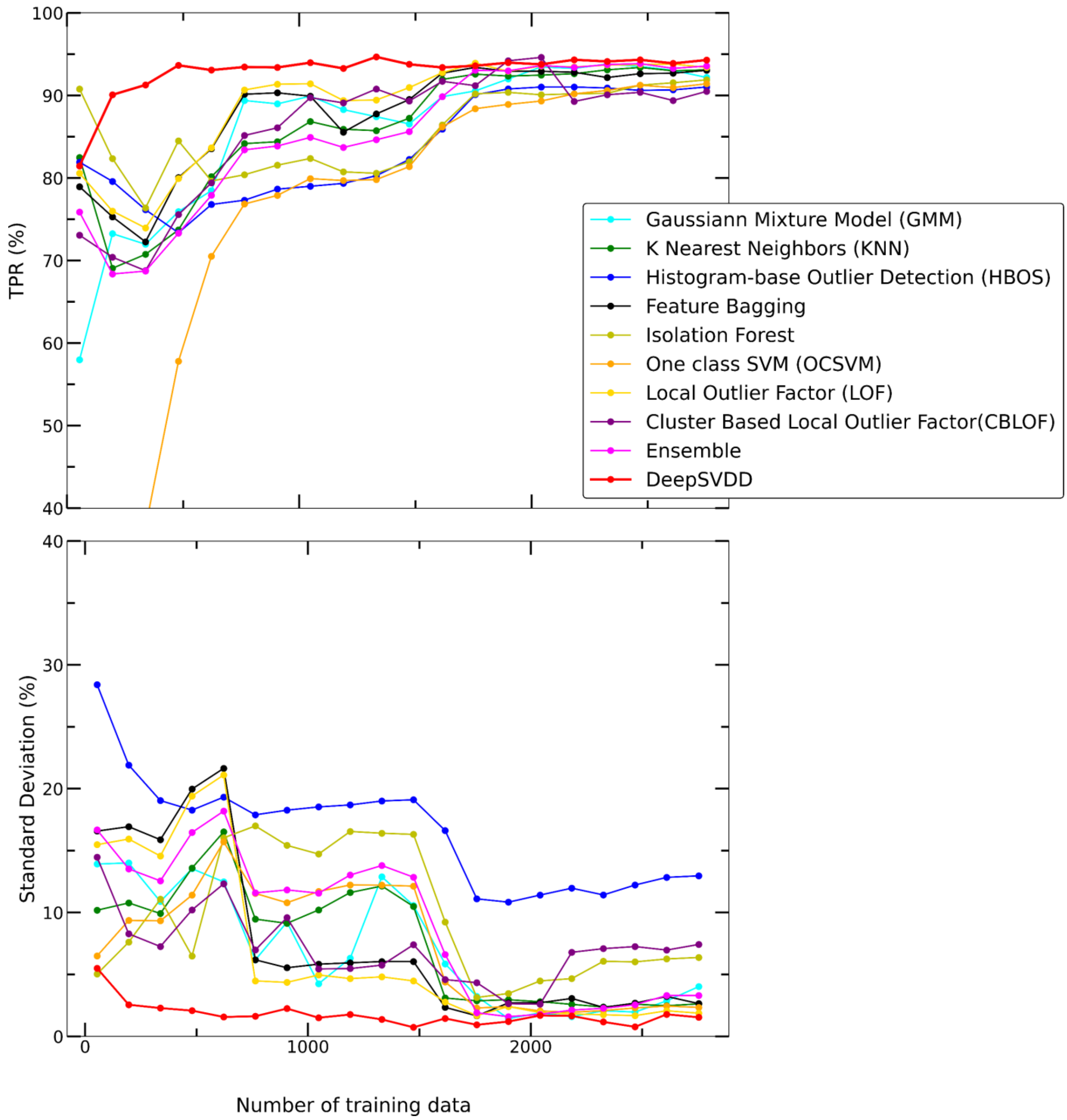

Figure S13. Learning curves (TRP %) and standard deviation (%) of all the implemented models.

3. Visualizing the predicted pairs

The analysis of the outcomes of the two workflows, *i.e.,* the standard approaches and the deep one class, is performed as following:

i) The top scored pairs are shown and compared with the closest scores-wise structure of the labelled dataset (training set).

ii) The most popular co-formers are identified by counting how many times each co-former is found in high score pairs (upper quartile)

iii) The predicted pairs are separated into lists based on the following criteria:

a) No constrains
b) Pairs after removing solvents
c) Pairs including one of the initial PAHs
c) Pairs without solvents and heteroatoms
e) Pairs including heteroatoms
f) Pairs including 1,6 dicyanoanthracene, the most similar (Tanimoto Similarity) molecule to TCNQ (well known for the electronic properties)
g) Pyrene-cocrystals

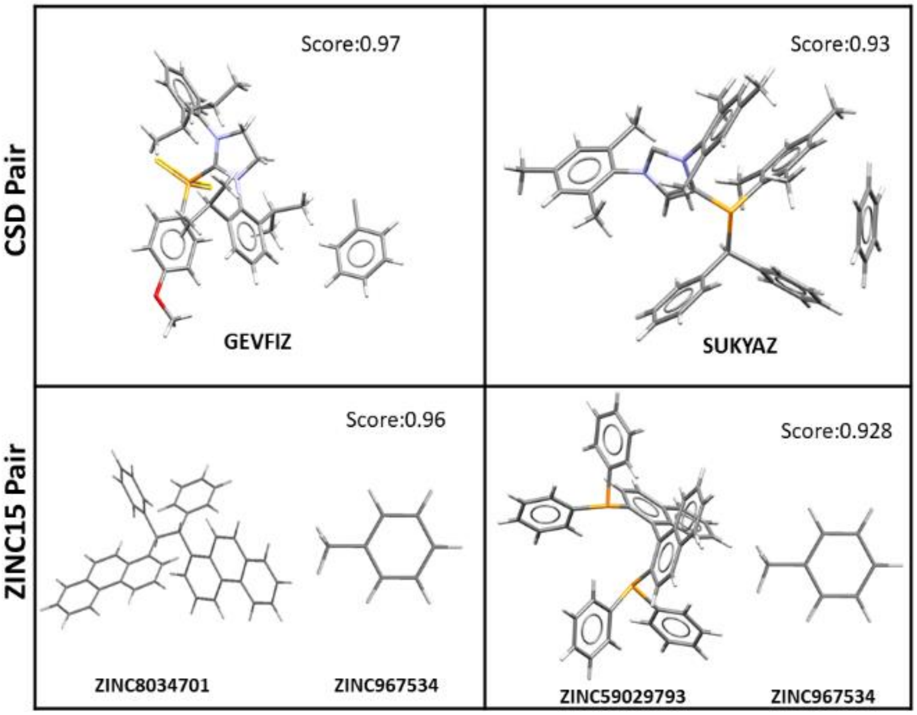

Figure S14. Examples of top scored combinations and most similar score-wise CSD entry for both workflows followed. On the first row we can observe the existing CSD molecular pairs, whereas on the second row is the closest in score predicted pair among those in ZINC15. Some of these trends could be seen in one of the high score pairs shown in Figure 6. It is easily observed that in this pair, the one co-former has both high molecular weight and distinctive branching index, whereas the pairing molecule lacks both a high molecular weigh and branching. This is in good accordance with the trends seen in the molecular weight and branching index between pairs. Obsiously the high score is not only based on these two descriptors as a wide range of different descriptors is taken into consideration of the deep learning model.

3.1. Ensemble Predictions

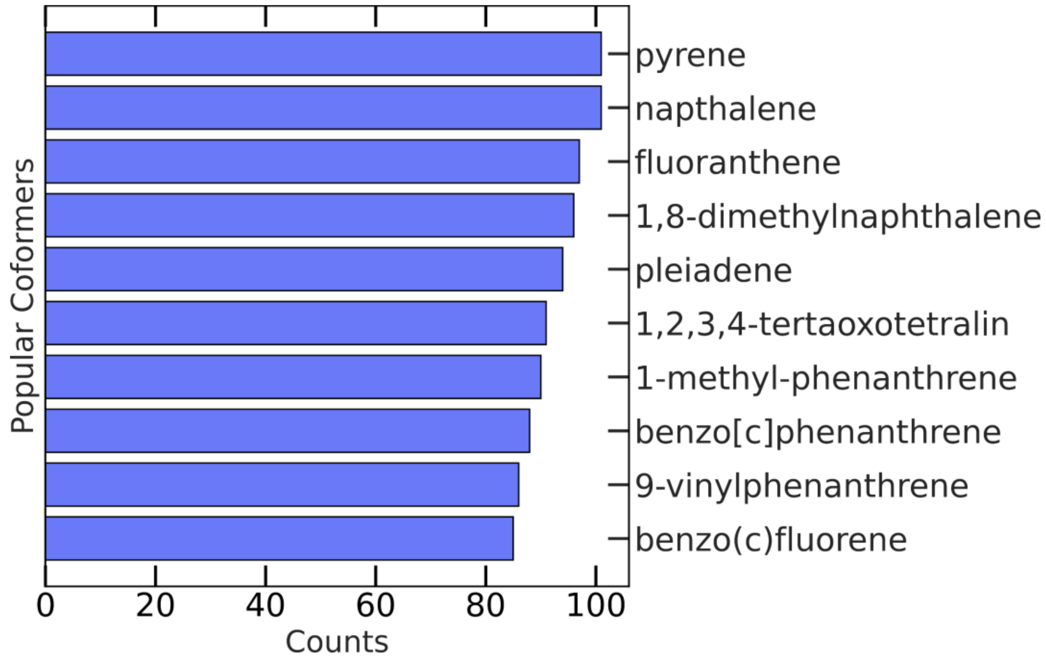

Figure S15. Bar chart of the top ten co-formers forming high scoring pairs according to the ensemble, that belong to the top quartile of the unlabelled dataset.

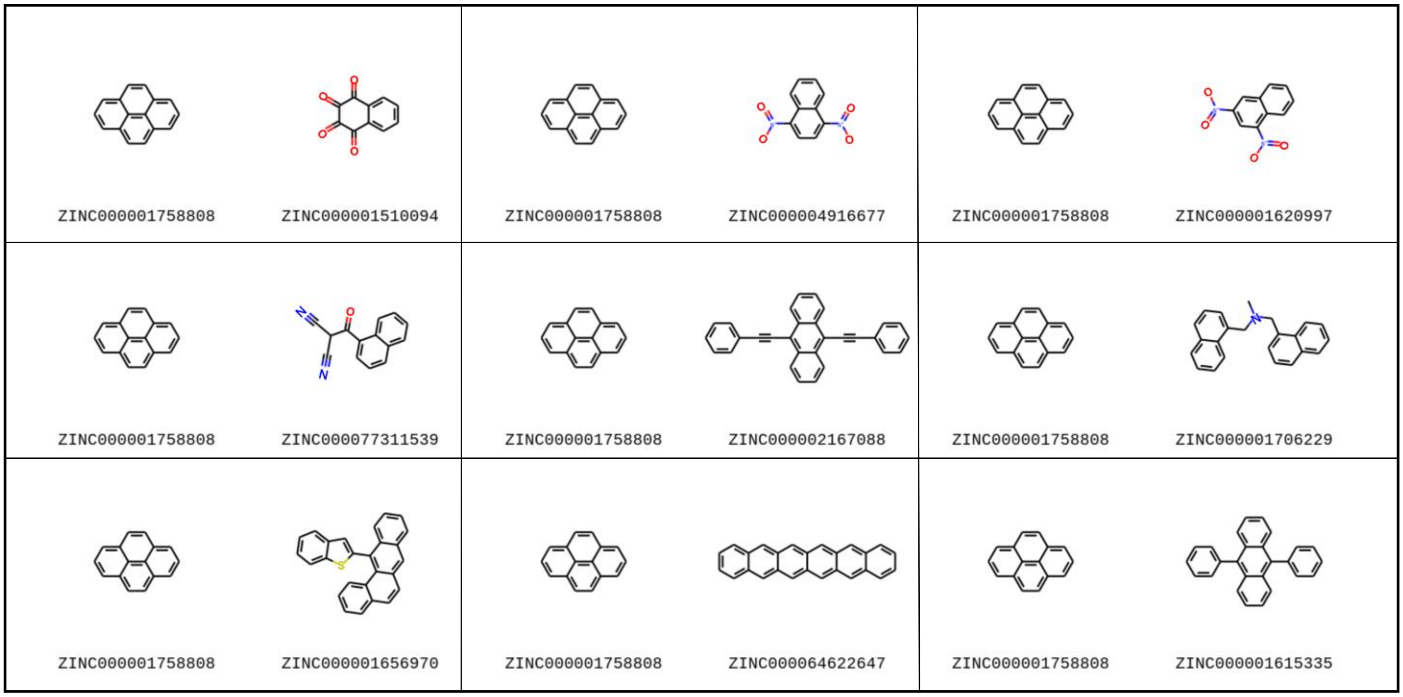

Figure S16. Predicted high score pairs with pyrene as a co-former, after removing the solvents, as predicted by the ensemble method.

3.2. Deep One Class predictions

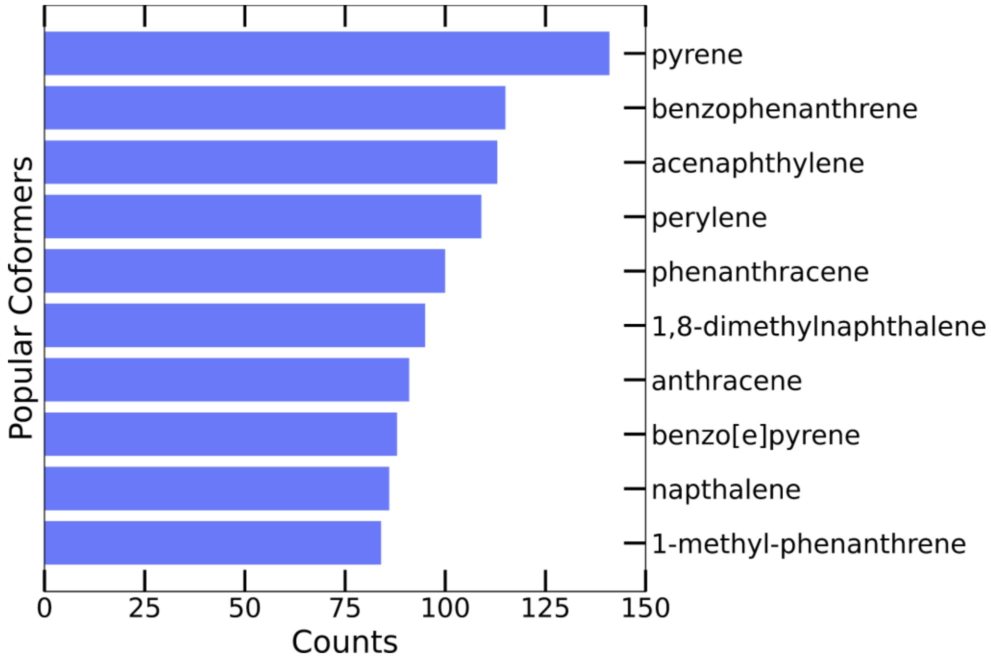

Figure S17. Bar chart of the top ten co-formers forming high scoring pairs that belong the top quartile of the unlabelled dataset. Pyrene appears as the most popular co-former among the top quantile.

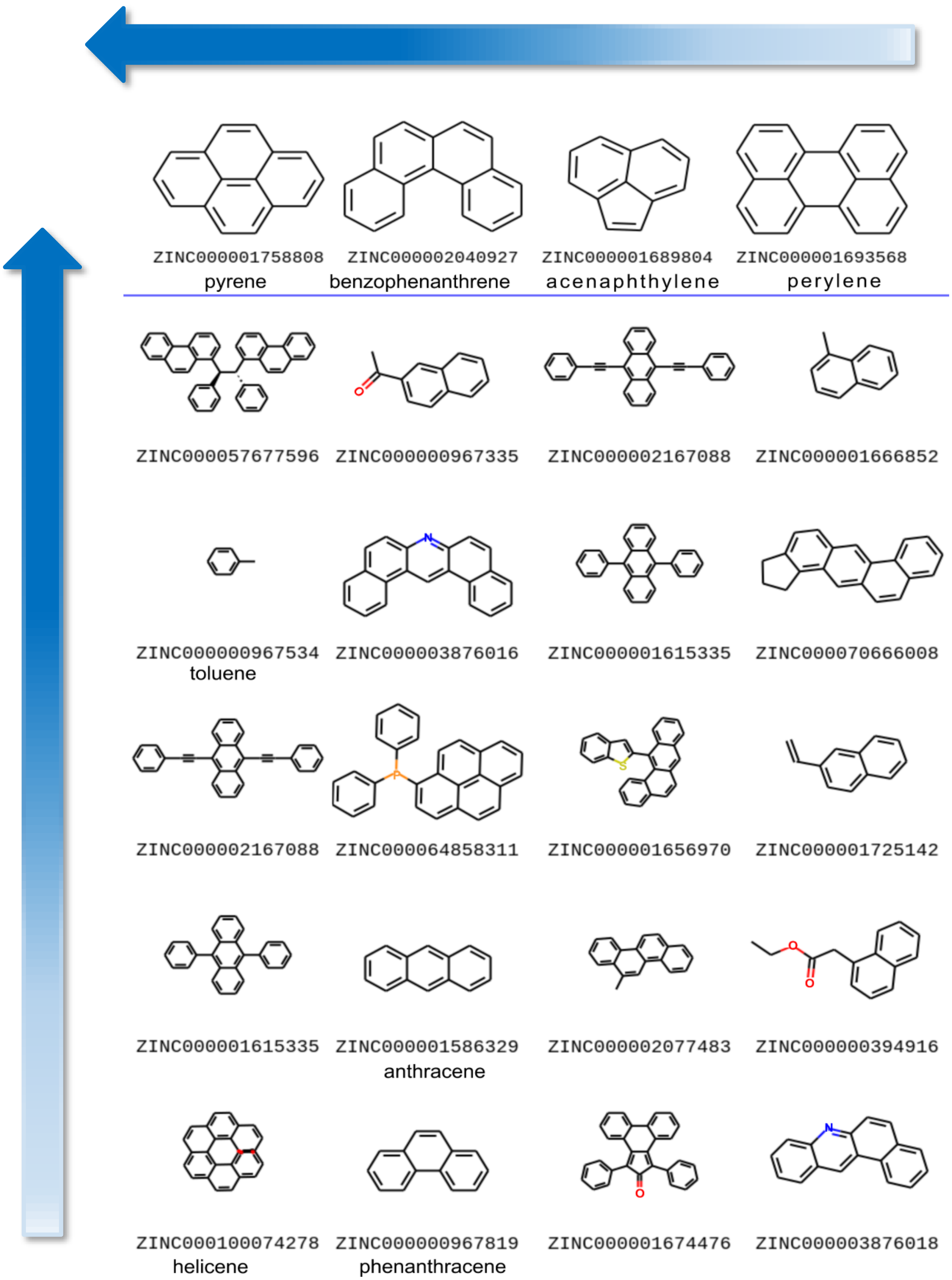

Figure S18. Molecular pairs formed by the most popular co-formers as predicted using the deep learning approach. Pyrene was identified as the most popular co-former as the majority of the possible pyrene co-crystals were assigned with high scores. The arrows indicate the direction of higher score (vertical arrow) and higher popularity (horizontal arrow).

Table S5. Top scoring pairs with no constrains.

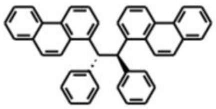 ZINC000008034701	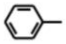 ZINC000000967534	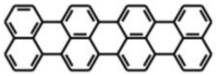 ZINC000064624955	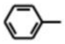 ZINC000000967534	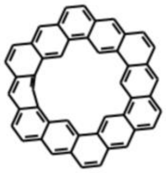 ZINC000100074226	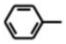 ZINC000000967534
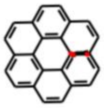 ZINC000100074278	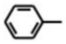 ZINC000000967534	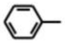 ZINC000000967534	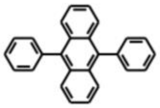 ZINC000001615335	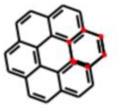 ZINC000100074293	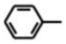 ZINC000000967534
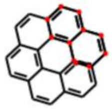 ZINC000100074301	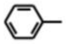 ZINC000000967534	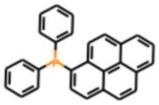 ZINC000064858311	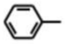 ZINC000000967534	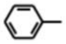 ZINC000000967534	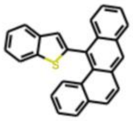 ZINC000001656970

Table S6. Top scoring pairs after removing the benzene-like solvents.

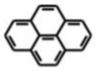 ZINC000001758808	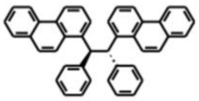 ZINC000057677596	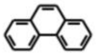 ZINC000000967819	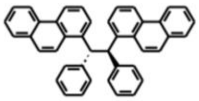 ZINC000008034701	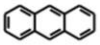 ZINC000001586329	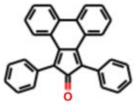 ZINC000001674476
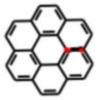 ZINC000100074278	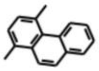 ZINC000001849773	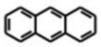 ZINC000001586329	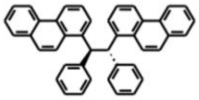 ZINC000057677596	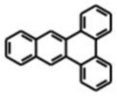 ZINC000002242728	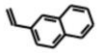 ZINC000001725142
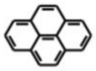 ZINC000001758808	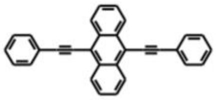 ZINC000002167088	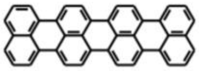 ZINC000064624955	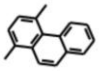 ZINC000001849773	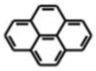 ZINC000001758808	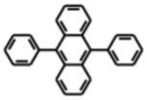 ZINC000001615335

Table S7. Top-scored predictions that include heteroatoms.

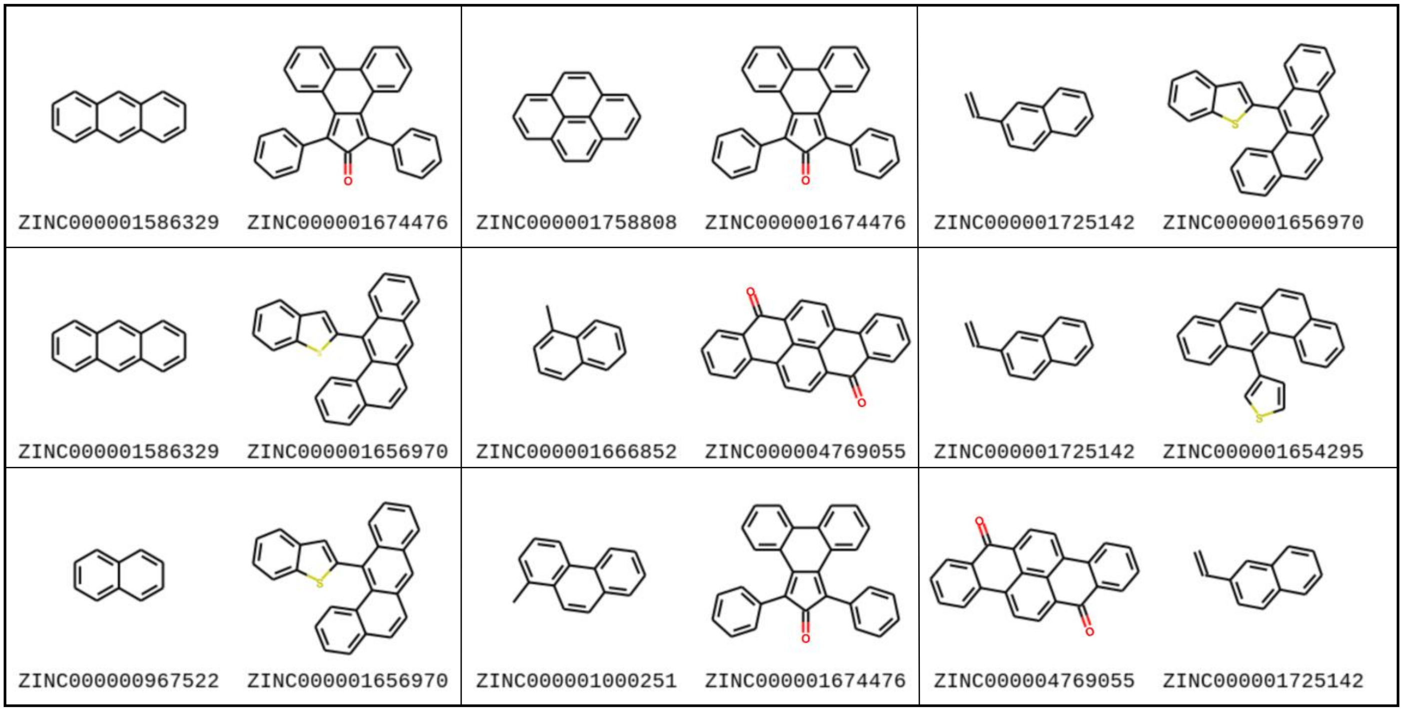

Table S8. Top scored pairs with at least one of the initial molecules.

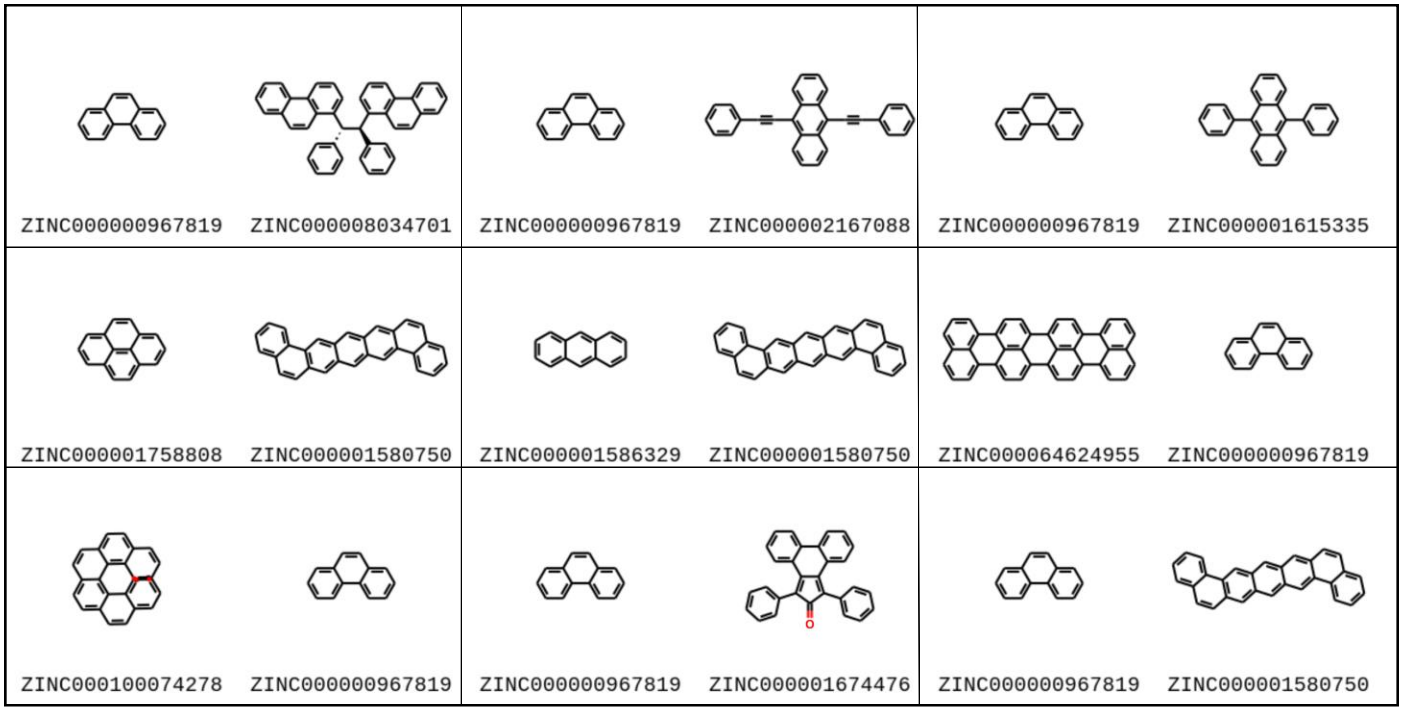

Table S9: Predictions which include 9,10-dicyanoathracene molecule.

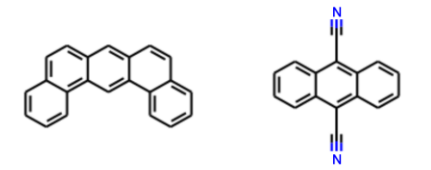 ZINC000001580747 ZINC000002584246	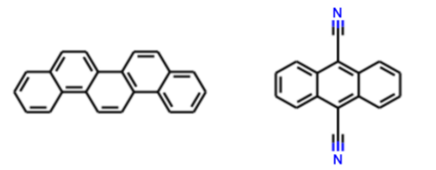 ZINC000001598876 ZINC000002584246	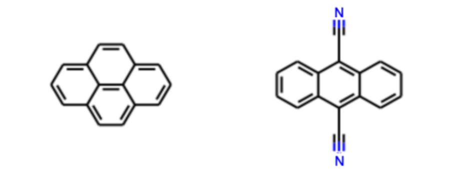 ZINC000001758808 ZINC000002584246
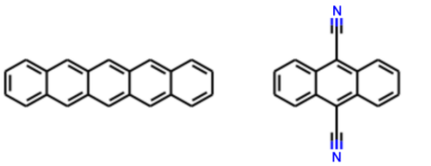 ZINC000001581013 ZINC000002584246	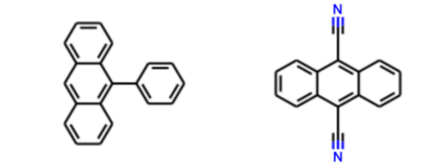 ZINC000001581017 ZINC000002584246	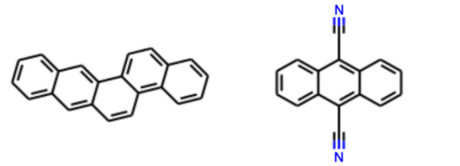 ZINC000001570231 ZINC000002584246
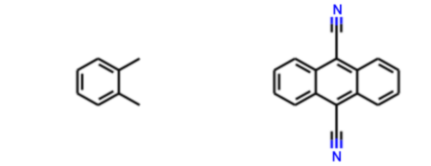 ZINC000000968282 ZINC000002584246	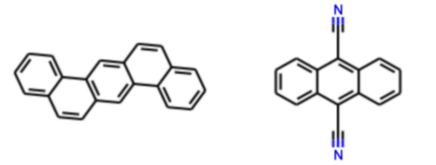 ZINC000001590020 ZINC000002584246	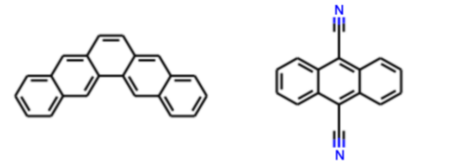 ZINC000002558787 ZINC000002584246

Table S10: Predictions which include 6H-benzo[c]chromen-6-one molecule.

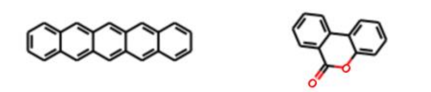 ZINC000001581013 ZINC000000401218	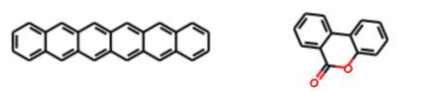 ZINC000064622647 ZINC000000401218	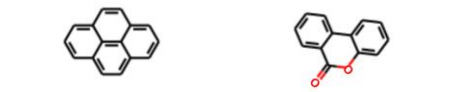 ZINC000001758808 ZINC000000401218
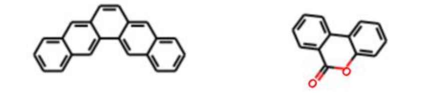 ZINC000002558787 ZINC000000401218	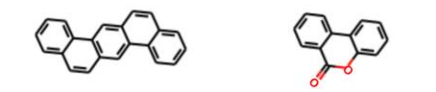 ZINC000001590020 ZINC000000401218	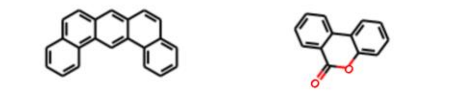 ZINC000001580747 ZINC000000401218
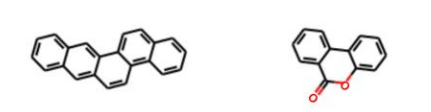 ZINC000001570231 ZINC000000401218	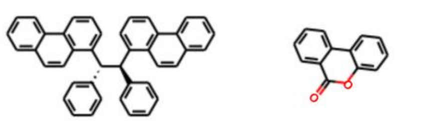 ZINC000008034701 ZINC000000401218	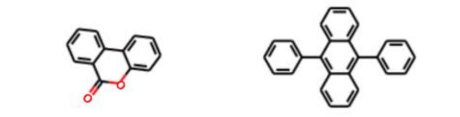 ZINC000000401218 ZINC000001615335

Table S11: Predictions which include pyrene.

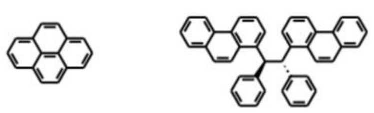<br>ZINC000001758808 ZINC000057677596	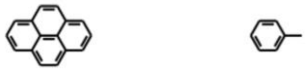<br>ZINC000001758808 ZINC000000967534	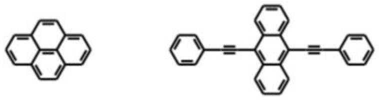<br>ZINC000001758808 ZINC000002167088
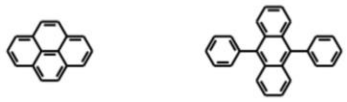<br>ZINC000001758808 ZINC000001615335	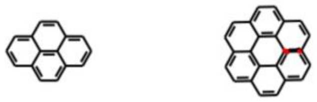<br>ZINC000001758808 ZINC000100074278	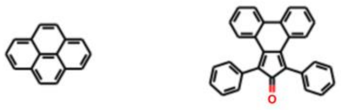<br>ZINC000001758808 ZINC000001674476
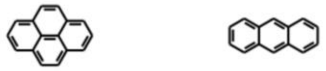<br>ZINC000001758808 ZINC000001586329	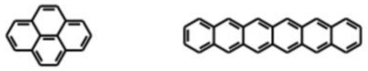<br>ZINC000001758808 ZINC000064622647	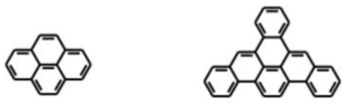<br>ZINC000001758808 ZINC000070667148

4. Predicting Molecular Stoichiometry

For the prediction of molecular stoichiometry on the labelled dataset the XGBoost classifier was implemented. The hyperparameters were optimized using the hyperopt library.

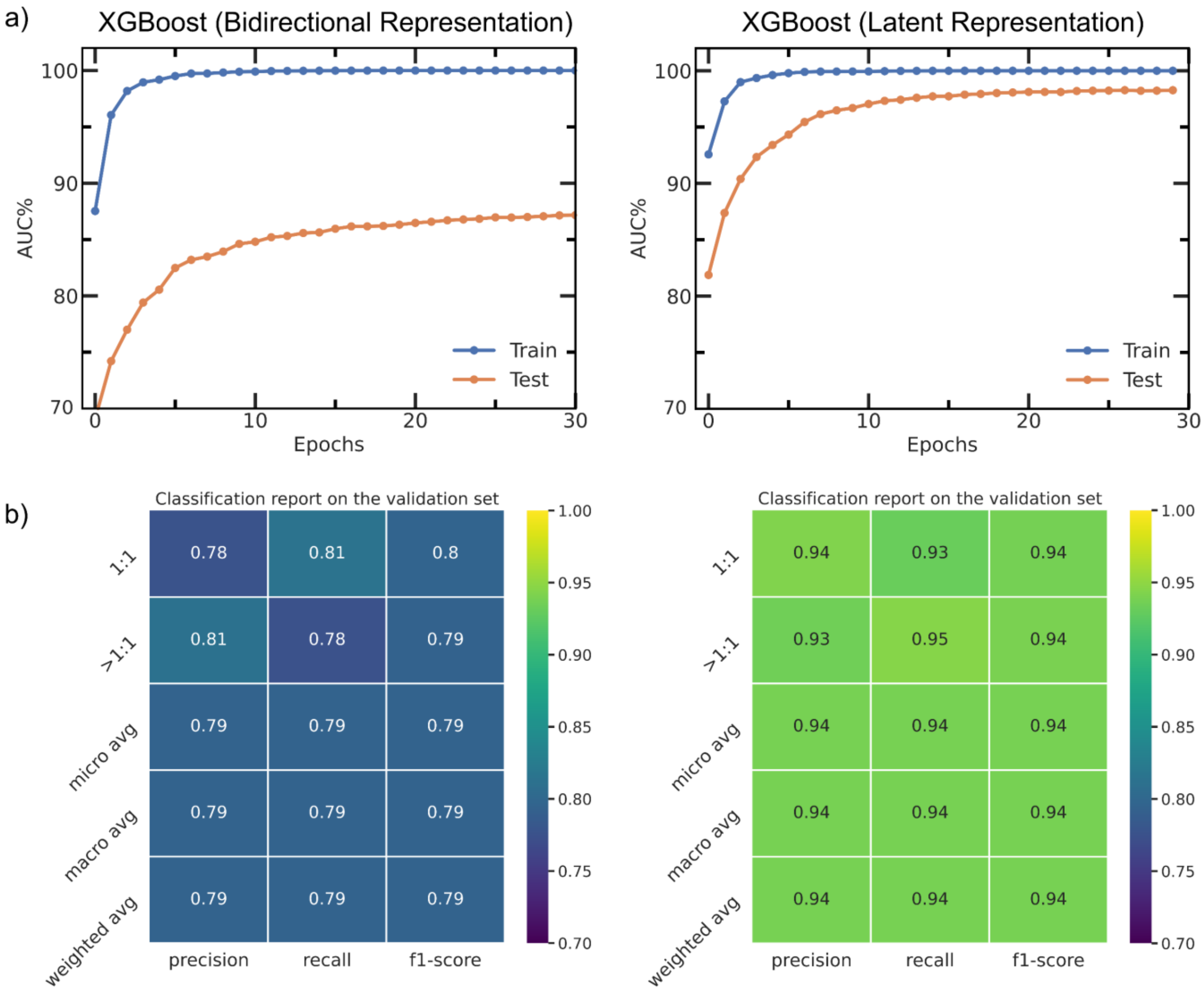

Figure S19. Evaluation metrics on the ratios prediction. a) Training/test accuracy with XGBoost Classifier on the prediction of ratios using the initial bidirectional dataset (left) and after using the latent representation (right). The highest accuracy achieved on the test set was 77% whilst overfitting on the training data. When the latent dimension of the deep network was used as the input the training was more effective, achieving more than 90% accuracy and no-overfitting. It can be postulated that using an Attention-based encoder for capturing the relation between the molecular pairs, a better representation can be achieved. b) Classification reports on the validation set using the bidirectional dataset (left) and the latent representation (right).

Table S12: Predicted ratios for the high-scored pyrene co-crystals.

Molecular Pair	Ratio
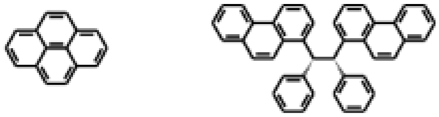	**1:1**
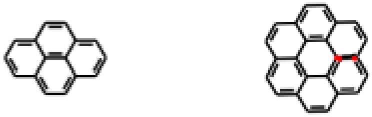	**1:1**
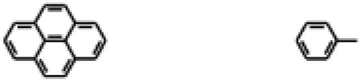	**1:1**
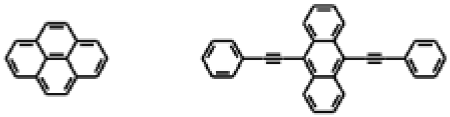	**1:1**
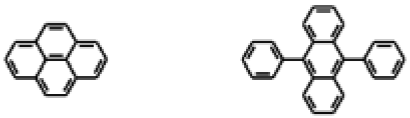	**1:1**
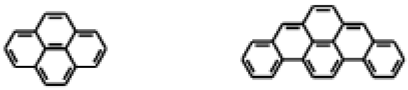	**>1:1**
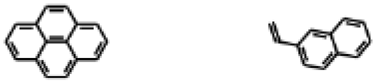	**>1:1**

5. Interpretability

SHAP (Shapley Additive exPlanations) was implemented as a model interpretation framework for providing chemical insights into predictions. Rationalizing model decisions would assign priority to meaningful experimental attempts and help the chemists to choose the next molecular pair worth testing. SHAP is a model independent method, meaning that it is not takes into consideration the feature weights but measures the influence each feature change has on the final decision of the model. In other words, by calculating Shapley values, the contribution of each feature of each combination to the final score is estimated.

The overall SHAP formula is shown in equation (1), where g is the explanation model, M is the number of simplified input features, $\varphi_i \in \mathbb{R}$ is the feature attribution for a feature i, $z' \in \{0,1\}^M$, and φ_0 represents the model output with all the simplified inputs missing.

$$g(z') = \varphi_0 + \sum_{i=1}^{M} \varphi_i z'_i \qquad (1)$$

To obtain the contribution of a feature i, all operations by which a feature might have been added to the set (N!) and a summation over all possible sets (S) is considered. For any feature sequence, the marginal contribution through addition of feature i is given by [f(S∪{i}) − f(S)], where f(S) corresponds to the output of the ML model. The resulting quantity is weighted by the different possibilities the set could have been formed prior to feature i's addition (|S|!) and the remaining features could have been added ((|N| − |S| − 1)!). Hence, the importance of a given feature is defined by equation (2):

$$\varphi_i = \frac{1}{N!} \sum_{S \subseteq \mathbf{N} \setminus \{\mathbf{i}\}} |S|!\,(|N| - |S| - 1)!\,[f(S \cup \{i\}) - f(S)] \qquad (2)$$

It follows that Shapley values represent a unique way to divide a model's output among feature contributions satisfying three axioms: local accuracy (or additivity), consistency (or symmetry), and nonexistence (or null effect).

Using the SHAP approach, the identification and prioritization of features that determine the pairs ranking is enabled. In that way we can extract the connection between the molecular properties and co-crystallisation. In addition to model accuracy, the interpretability of the predictions is adding value to any machine learning model.

High negative Shapley values are driving the model towards outliers, whereas as high positive values are supporting the decision for inlierness. Initially, we tried to get the whole picture of the model and have an indication for the important features that dominate the training set. GradientExplainer method was used on the whole bidirectional dataset such that the position of the molecule will not matter. The summary plot with the features' contribution in descending order of importance is shown in Figure S20. The red and blue values indicate high and low values respectively, with high positive red enhancing the decision of inlierness and high negative red driving towards the decision of anomaly. The feature value reflects the contribution the feature makes to the final score of the molecular pair.

Model interpretation inherently depends on the interpretability of the implemented descriptors or features. Herein, we used all the available Dragon descriptors as we wanted to compare with previous statistical analysis on the CSD based on similar descriptors and also to gain a physical meaning for the PAHs co-crystals to enable an experimental chemist to understand dominating patterns and prioritize the experimental work.

5.1 Interpreting the labelled dataset.

A density scatter plot of SHAP values was employed for illustrating the feature importance. Herein, it can be seen the impact each feature has on the model scoring for each individual pair in the labelled dataset. Features are sorted by the sum of the SHAP value magnitudes across all samples. In the plot the density of the datapoints can also be observed on the lumpier areas, as on the y axis the distribution of the datapoints is shown.

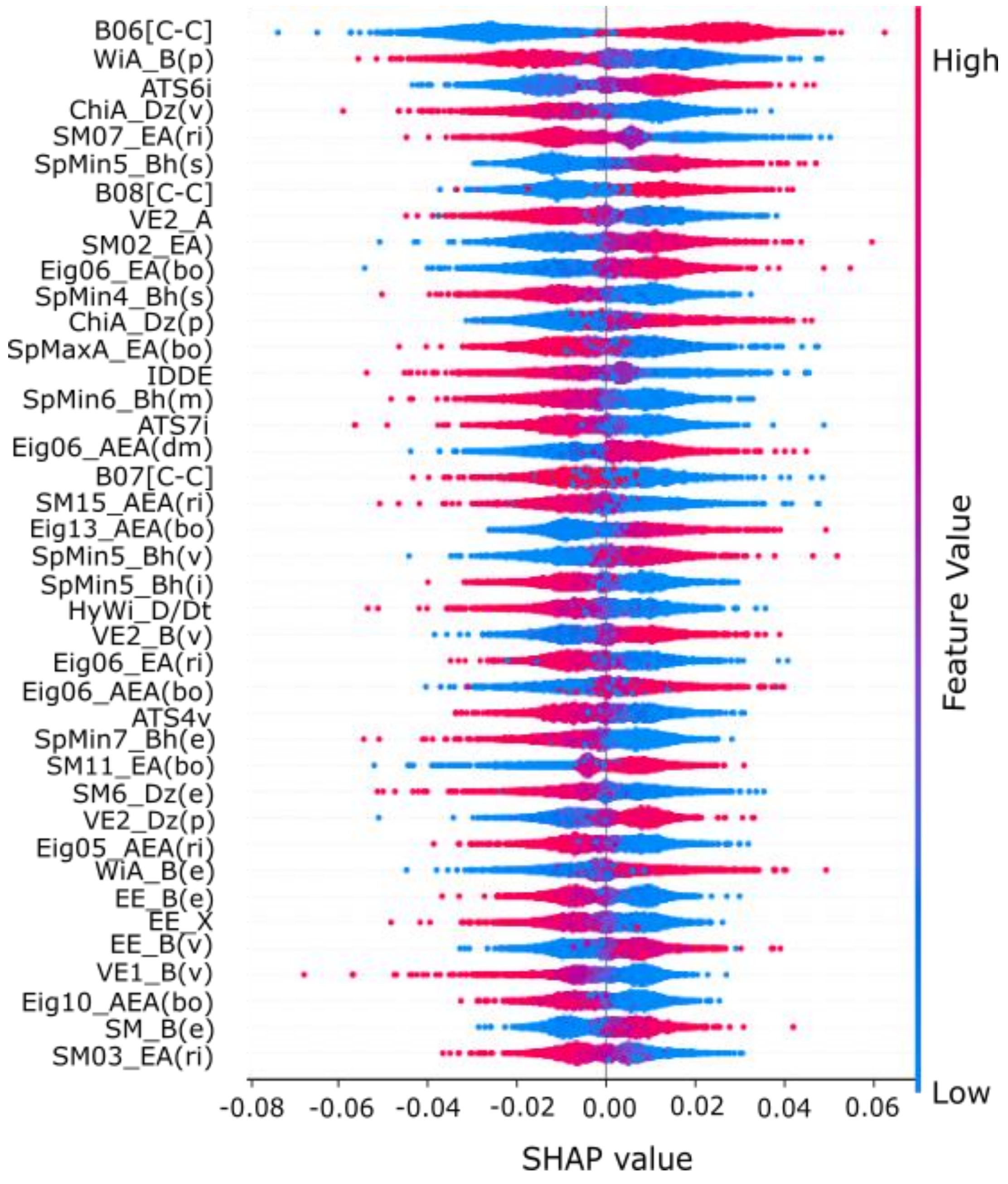

Figure S20. Summary plot of Shapley values for global interpretation of the bidirectional dataset. The Shapley value of each feature represents its contribution towards the model's output. Red positive values are driving to higher scores and boosting inlierness, whereas red negative values tend to descrease the final score and hence outlierness. Blue values are idicative of low feature value. As for many of the features calculated by Dragon software a physical meaning is hard to be extracted, the correlations among the most significant descriptors with those that are more general is calculated.

5.2 Interpreting the pyrene co-crystals subset.

The advantage of using the Shapley analysis is that we can zoom in to a subset of interest and gain some knowledge about the important features in some molecular pairs of interest. In the work, as pyrene was identified as a popular co-former and was used for the experimental screening, the pyrene co-crystals family of materials was further investigated to extract the feature importance and understanding which properties dominate in the existing pyrene pairs. As the pyrene is a set molecule, it took the first place on the molecular vector and the pairing molecules were always second. As we are interested in the contribution of the pairing molecule only the features related to them are shown in the summary plot below. For those molecules where shape matters, it has more impact than the existence of heteroatoms. It can be postulated that in cases where we have a pairing molecule with no heteroatoms, then the shape will play an importanct role in the pyrene co-crystal formation.

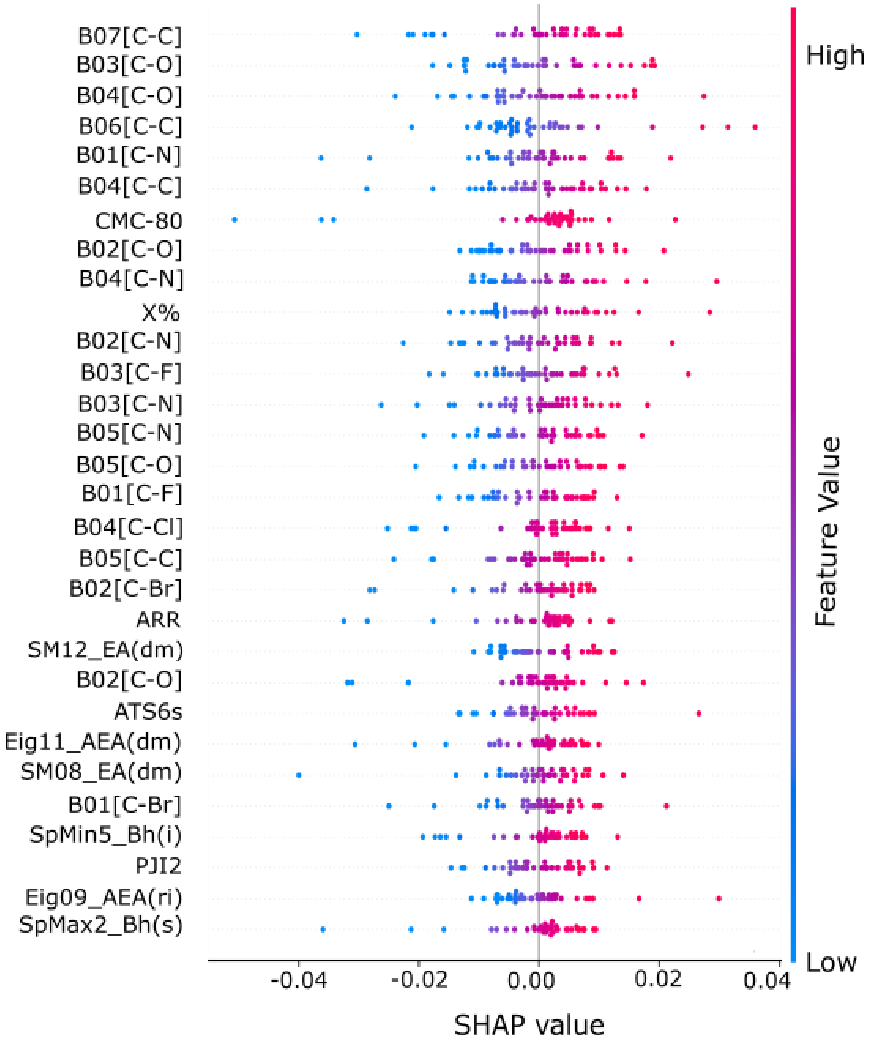

Figure S21. Shapley values showing the important descriptors for molecules pairing with Pyrene in the labelled dataset. Only the contributions of the second co-formers are shown here. The presence of heteroatoms in several topological distances in the molecule are those that seem to contribute more, as indicated by the B04[C-O], B03[C-O], B01[C-N], B04[C-N], X%, B02[C-F] descriptors. The notable elements are N and O. So we could expect that molecules with these groups in the certain topological distances and high scores (as the score is the outcome of the consideration of all the known features) are good candidated for forming co-crystals with Pyrene.

5.3 Important correlations between descriptors

Table S13. Descriptors correlated to the descriptors identified as important for the decisions of the deep learning model. The correlation between the descriptors follows a previously reported method.[17]

Descriptor	Correlated Descriptors	Correlation	Description	Related Physical Meaning
B06[C-C]	B07[C-C]	0.857434	Presence/absence of C - C at topological distance 7	atom pairs descriptors that describe pairs of atoms and bond types connecting them in 2D space
	B05[C-C]	0.812225	Presence/absence of C - C at topological distance 5	atom pairs descriptors that describe pairs of atoms and bond types connecting them in 2D space
ATS6i	ATS6e	0.998216	Broto-Moreau autocorrelation of lag 6 (log function) weighted by Sanderson electronegativity	electronegativity
	ATS5e	0.983335	Broto-Moreau autocorrelation of lag 5 (log function) weighted by Sanderson electronegativity	electronegativity
	ATS5i	0.981890	Broto-Moreau autocorrelation of lag 5 (log function) weighted by ionization potential	ionization potential
	SpMax8_Bh(i)	0.928269	largest eigenvalue n. 8 of Burden matrix weighted by ionization potential	Ionization potential
	SpMax8_Bh(p)	0.923641	largest eigenvalue n. 8 of Burden matrix weighted by polarizability	polarizability
	ATS8e	0.927747	Broto-Moreau autocorrelation of lag 8 (log function) weighted by Sanderson electronegativity	electronegativity
	Vx	0.913402	McGowan volume	shape
	Si	0.945914	sum of first ionization potentials (scaled on Carbon atom)	Ionization potential
	Se	0.940544	sum of atomic Sanderson electronegativities (scaled on Carbon atom)	electronegativity
	nBT	0.934793	number of bonds	general
	Sp	0.923744	sum of atomic polarizabilities (scaled on Carbon atom)	polarizability
	Sv	0.913610	sum of atomic van der Waals volumes (scaled on Carbon atom)	shape
	IAC	0.900917	total information index on atomic composition	composition

	S1K	0.887118	1-path Kier alpha-modified shape index	Shape
	Eta_epsi	0.875800	eta electronegativity measure	electronegativity
	SAtot	0.871258	total surface area from P_VSA-like descriptors	polarity
	Pol	0.863927	polarity number	polarity
	nSK	0.853433	number of non-H atoms	general
	MW	0.828710	Molecular weight	general
Eig06_AEA(dm):	Eig05_AEA(dm)	0.956601	eigenvalue n. 5 from augmented edge adjacency mat. weighted by dipole moment	dipole moment
	Eig7_AEA(dm)	0.938136	eigenvalue n. 7 from augmented edge adjacency mat. weighted by dipole moment	dipole moment
	Eig08_AEA(dm)	0.918267	eigenvalue n. 8 from augmented edge adjacency mat. weighted by dipole moment	dipole moment
	Ram	0.792930	Ramification	branching
	Eta_B	0.778573	eta branching index	Shape
ChiA_Dz(p)	SpMaxA_B(p)	0.910006	normalized leading eigenvalue from Burden matrix weighted by polarizability	polarizability
	WiA_B(p)	0.908640	average Wiener-like index from Burden matrix weighted by polarizability	polarizability
	ChiA_Dz(e)	0.901665	average Randic-like index from Barysz matrix weighted by Sanderson electronegativity	electronegativity
	UNIP	0.933653	unipolarity	Polarity
	Sv	0.822757	sum of atomic van der Waals volumes (scaled on Carbon atom)	shape
	MW	0.822103	Molecular weight	molecular weight
	VvdwMG	0.819518	van der Waals volume from McGowan volume	Shape
	Vx	0.819518	McGowan volume	shape
	Si	0.815686	sum of first ionization potentials (scaled on Carbon atom)	Ionization potential
	Pol	0.805521	polarity number	polarity
	Sp	0.795808	sum of atomic polarizabilities (scaled on Carbon atom)	polarizability

SpMin5_Bh (s)	ATS3i	0.921903	Broto-Moreau autocorrelation of lag 3 (log function) weighted by ionization potential	ionization potential
	ATS3e	0.917570	Broto-Moreau autocorrelation of lag 3 (log function) weighted by Sanderson electronegativity	electronegativity
	SpMin5_Bh (e)	0.915201	smallest eigenvalue n. 5 of Burden matrix weighted by Sanderson electronegativity	electronegativity
	Sv	0.898829	sum of atomic van der Waals volumes (scaled on Carbon atom)	shape
	Sp	0.895652	sum of atomic polarizabilities (scaled on Carbon atom)	polarizability
	Si	0.882950	sum of first ionization potentials (scaled on Carbon atom)	Ionization potential
	Se	0.881810	sum of atomic Sanderson electronegativities (scaled on Carbon atom)	electronegativity
	Vx	0.878079	McGowan volume	shape
	VvdwMG	0.878079	van der Waals volume from McGowan volume	shape
	MW	0.803832	Molecular weight	molecular weight
	Ram	0.800056	Ramification	shape
Eig06_EA(bo)	Pol	0.888838	Polarity number	polarity
	CSI	0.887028	eccentric connectivity index	shape
	UNIP	0.871951	unipolarity	polarity
	Sv	0.859414	sum of atomic van der Waals volumes (scaled on Carbon atom)	shape
	MW	0.834828	Molecular weight	general
	Ram	0.831023	Ramification	branching
	Vx	0.818124	van der Waals volume from McGowan volume	shape
	VvdwMG	0.818124	van der Waals volume from McGowan volume	Shape
	Sp	0.811851	sum of atomic polarizabilities (scaled on Carbon atom)	polarizability

5.4 Descriptor distributions

As the main principle of machine learning is to encounter some underlying structure in the data, we visualize the distribution of the labelled dataset, used as the training set and the extracted distribution of the unlabelled dataset to compare the general patterns. The investigated properties are separated into the following categories: i) General descriptors, ii) Shape descriptors, iii) Polarity descriptors, iv) Size descriptors and v) Electronic descriptors.

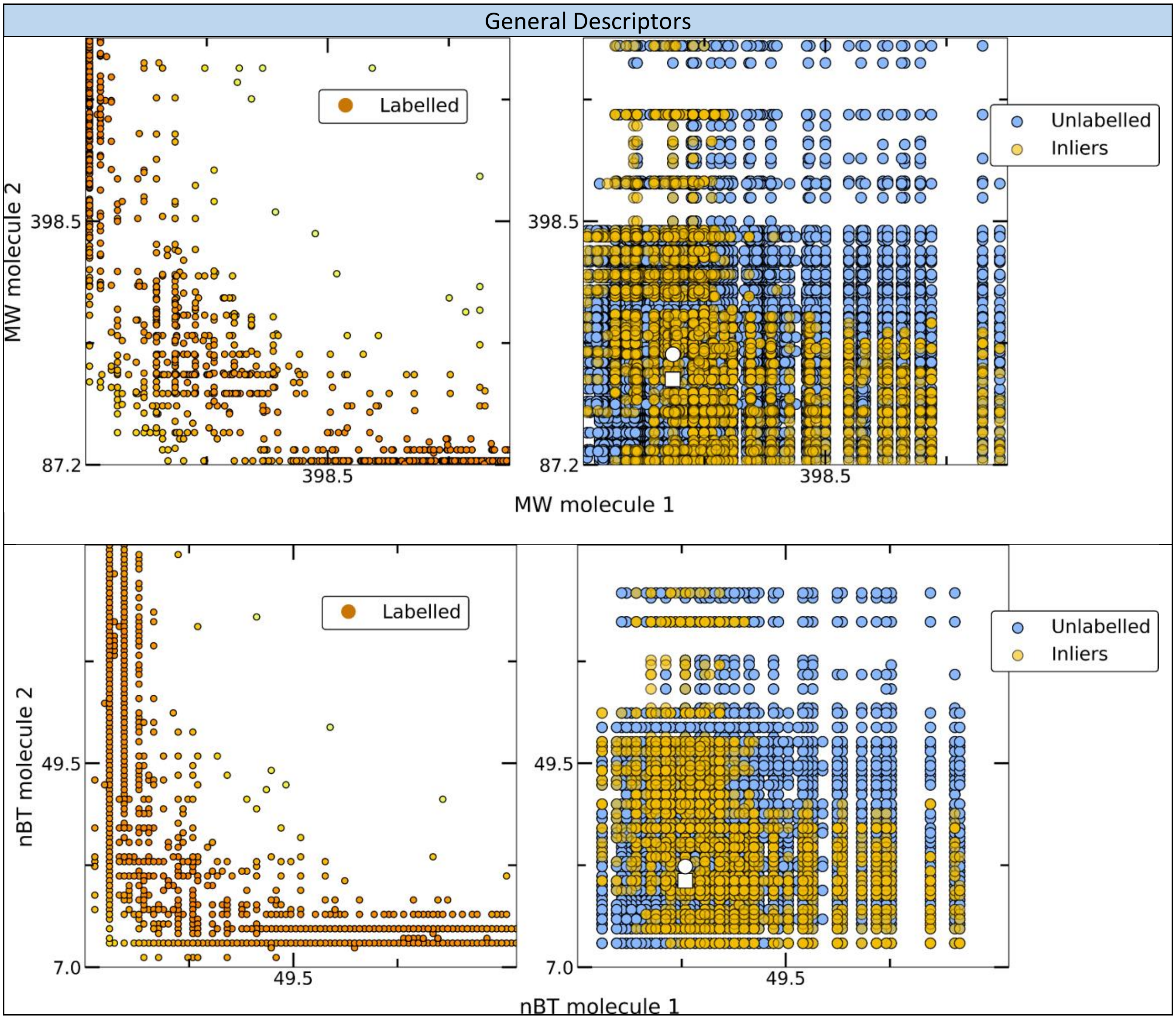

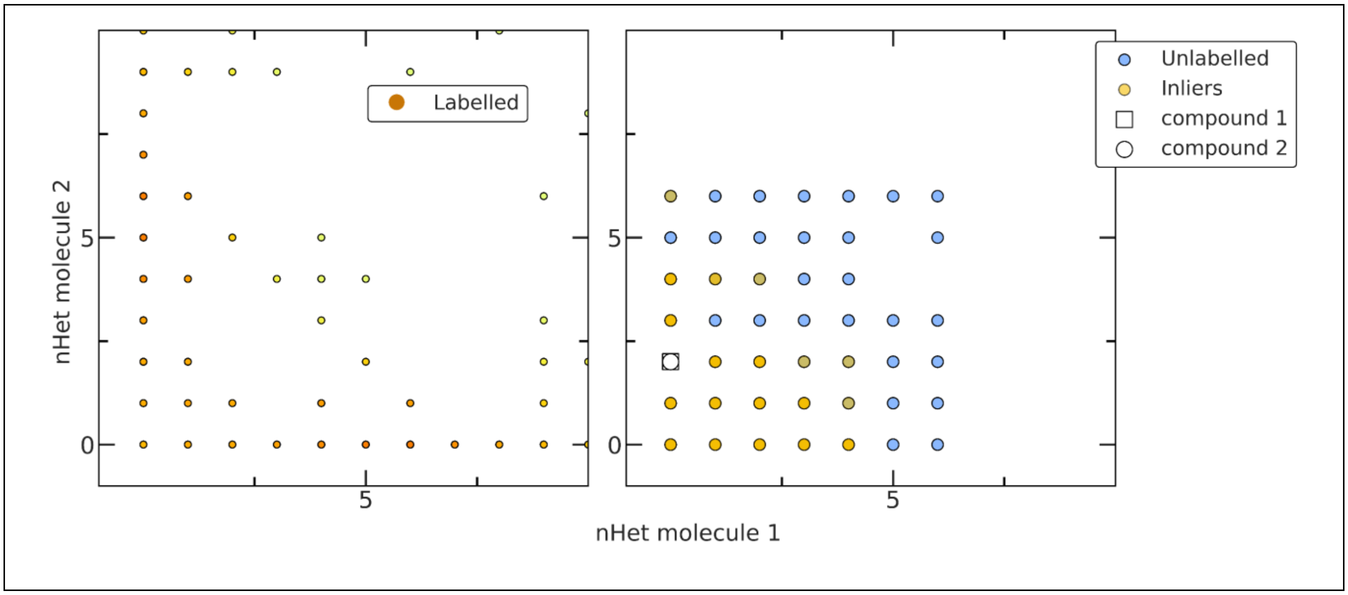

Figure S22. Extracted Patterns from the Deep learning model for some important general descriptors.

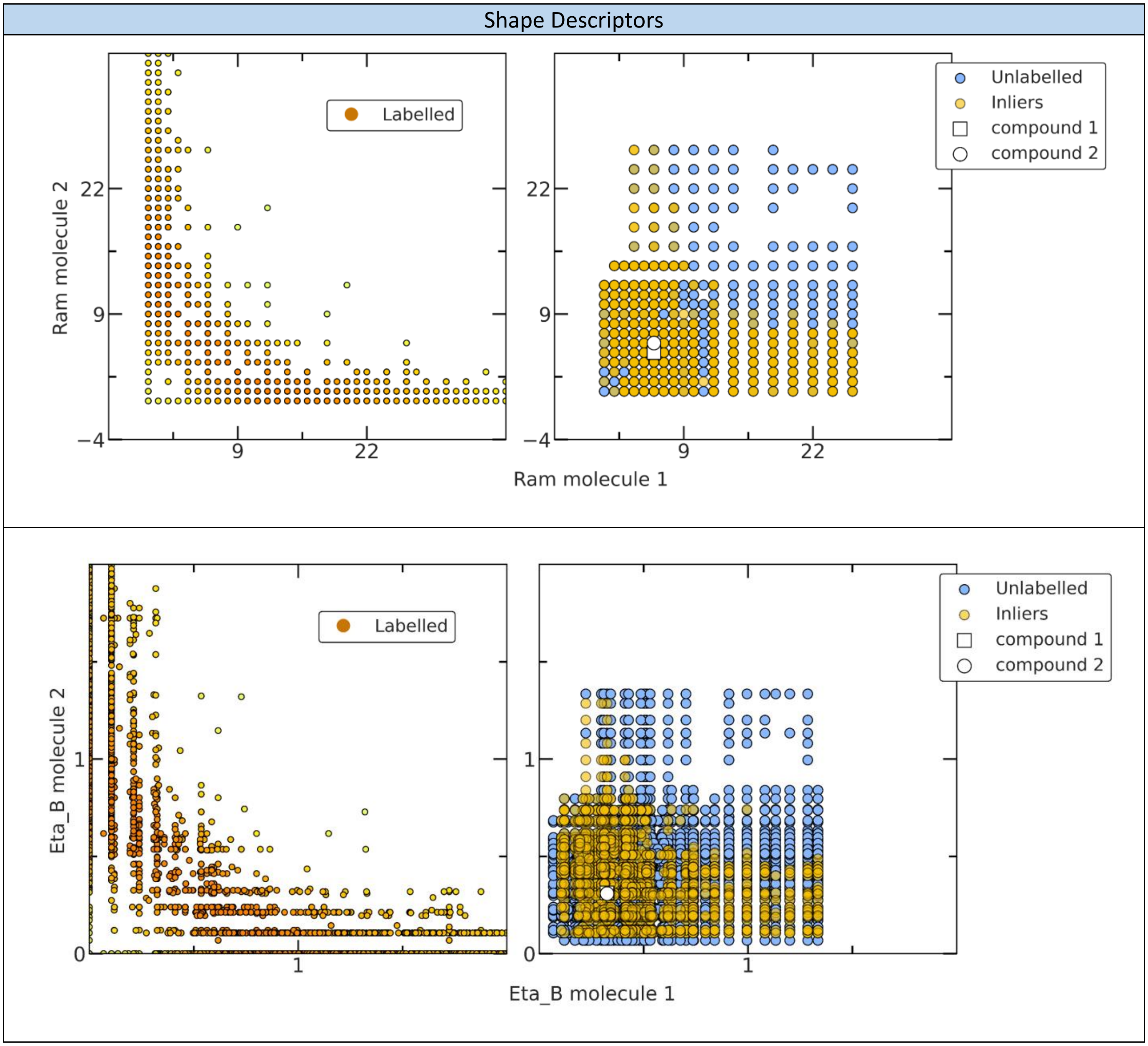

Figure S23. Extracted Patterns from the Deep learning model for shape descriptors.

Polarity Descriptors

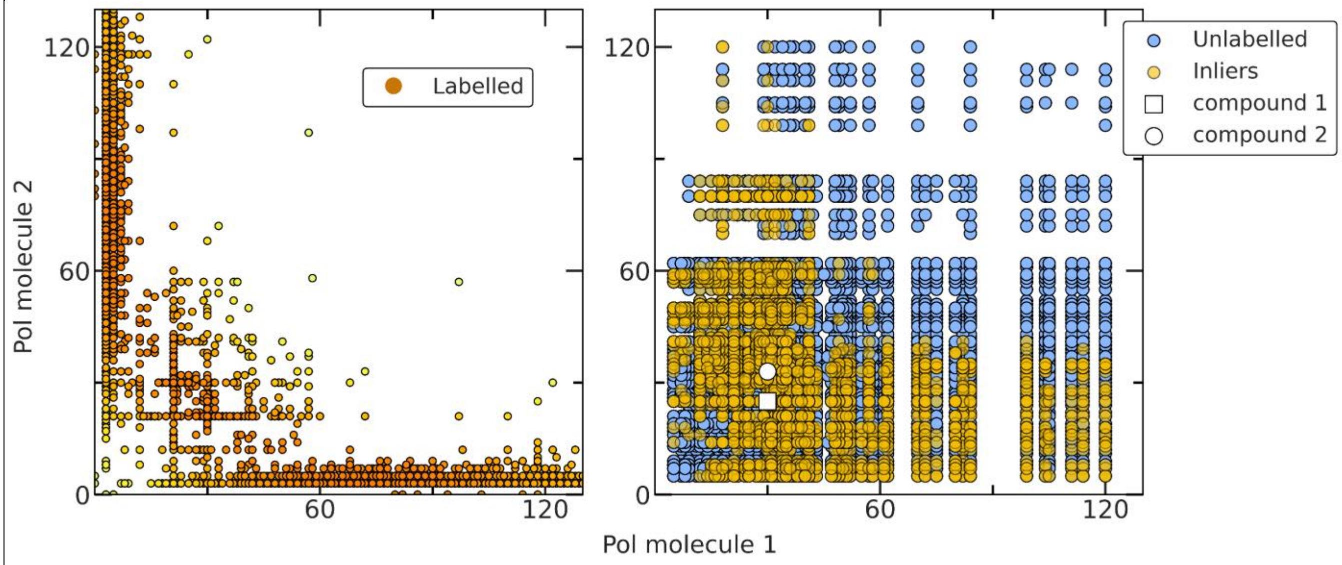

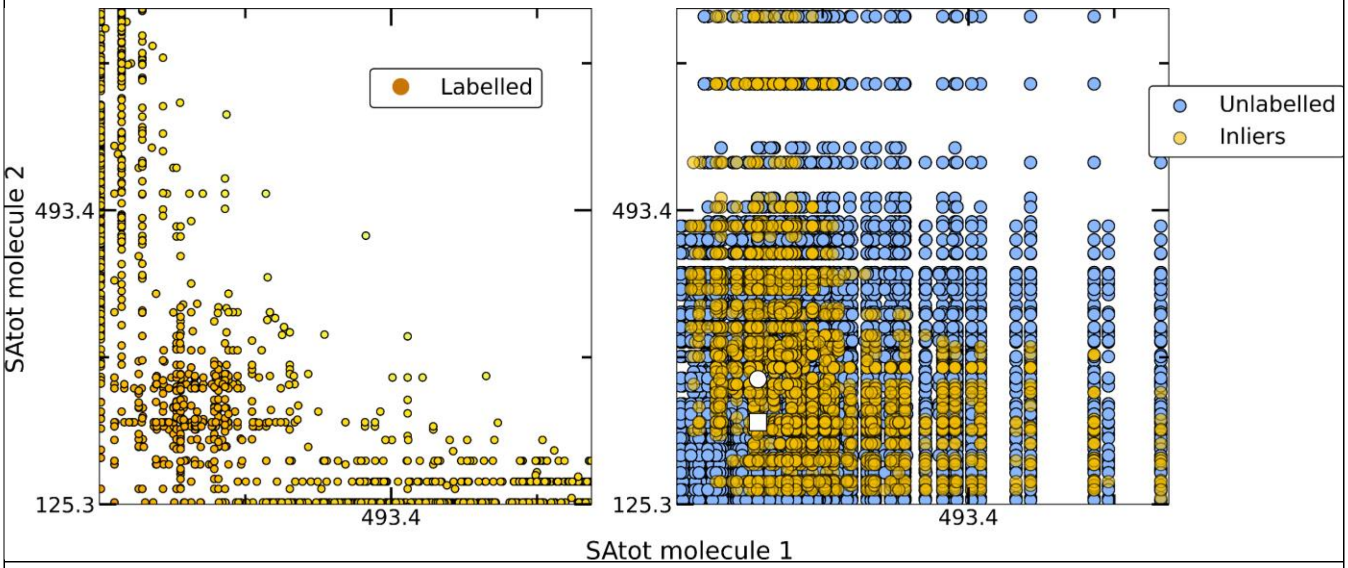

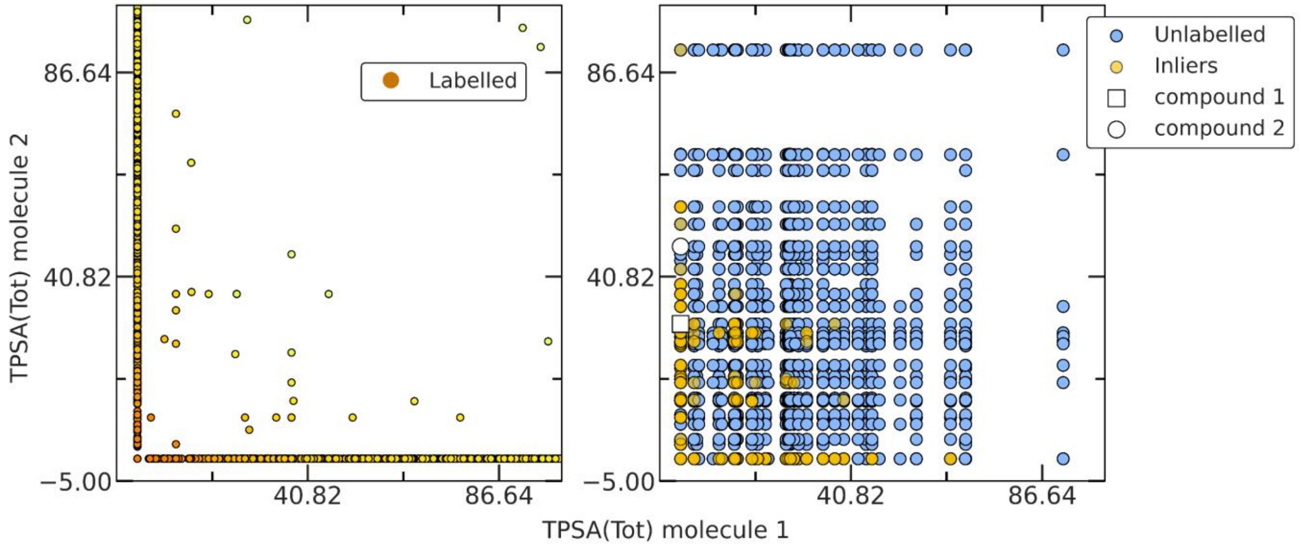

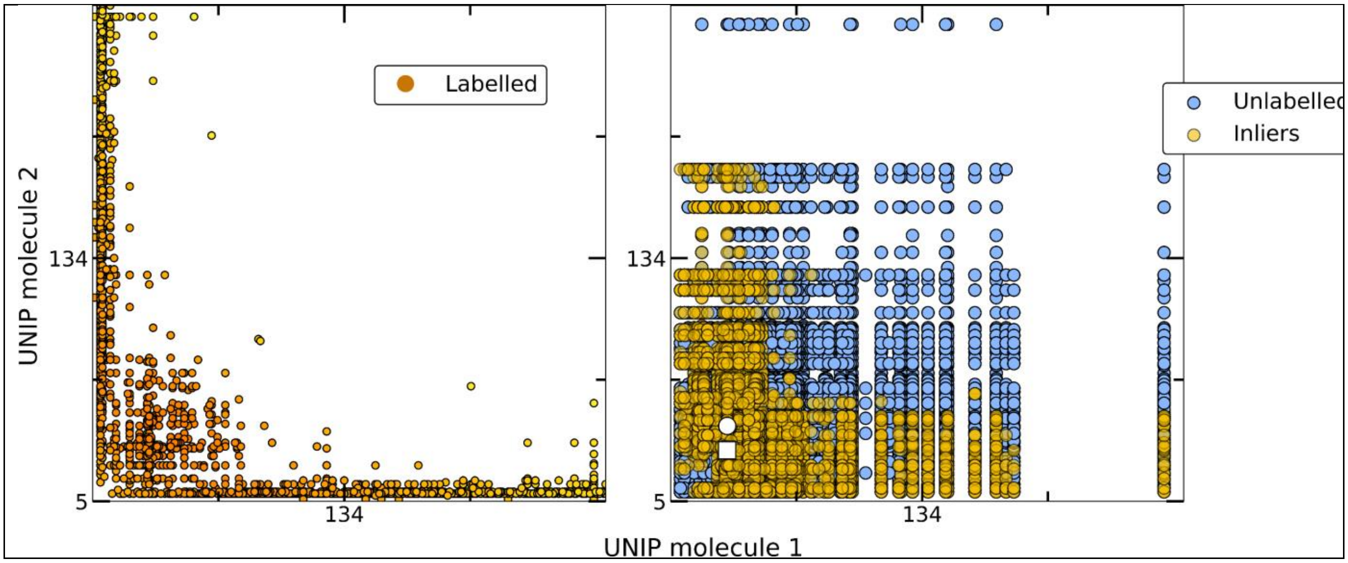

Figure S24. Polarity descriptors distribution.

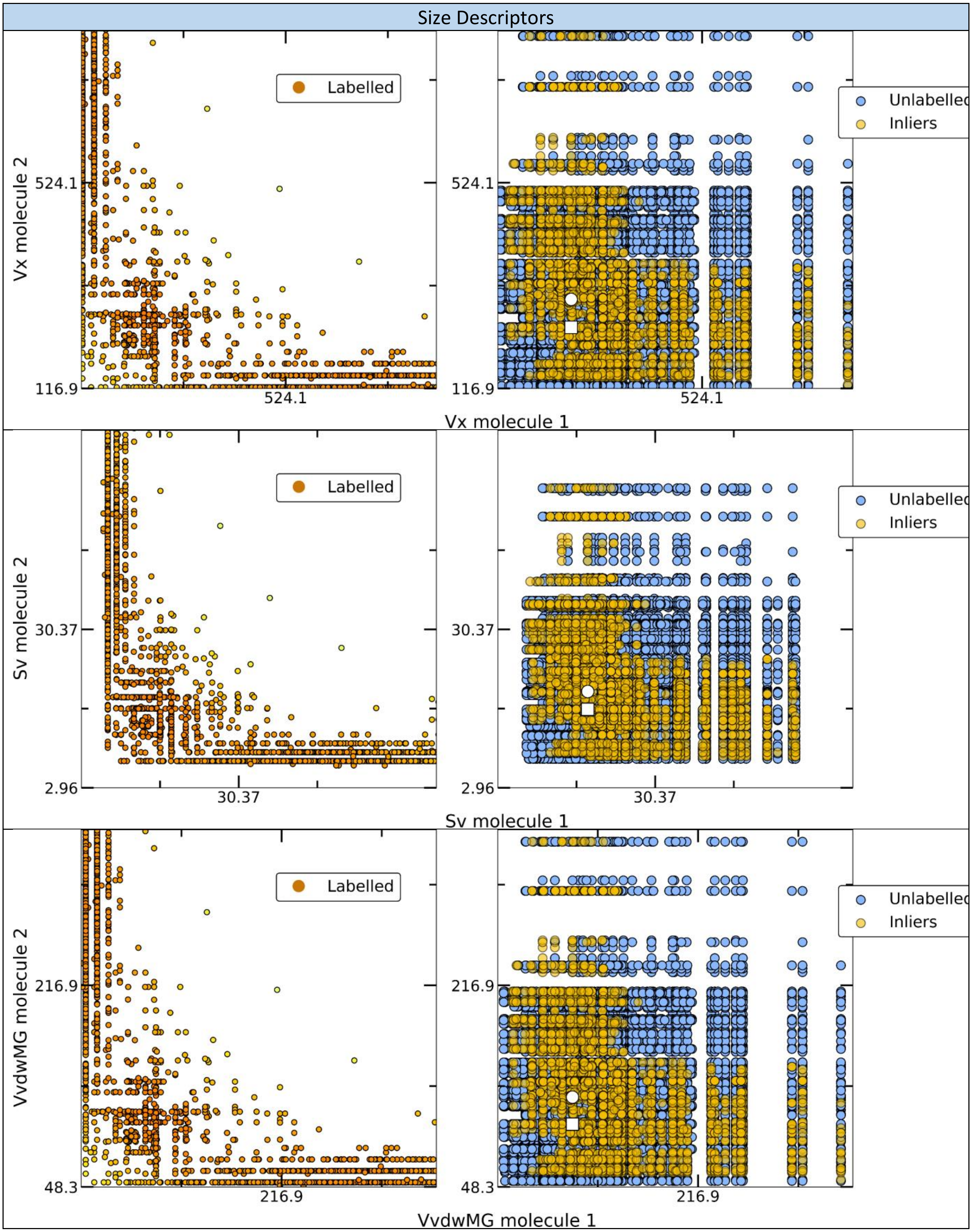

Figure S25. Size descriptors.

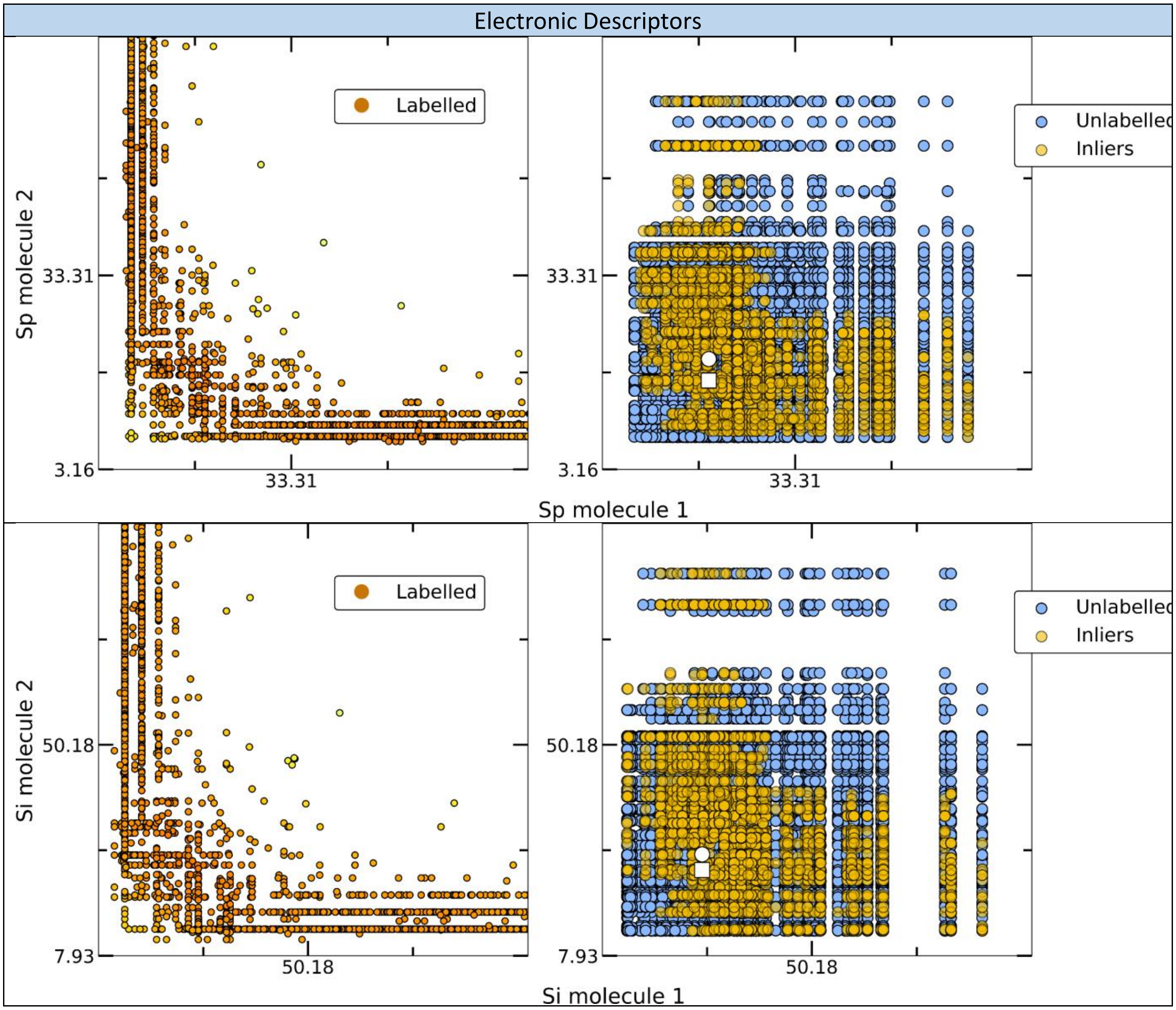

Figure S26. Electronic descriptors.

6. Experimental Realization (Pareto Optimization)

As our target is to design functional materials, for the selection of the co-formers to be experimentally tested some further important parameters are taken into consideration. These parameters refer to common factors that a synthetic chemist will use as a guideline for the experimental design: quick availability, novelty and possible electronic properties. The decision making was driven by a commonly used criterion for determining solutions to multi-objective optimization problems, the Pareto optimality.[18] The co-formers to be tested are the Pareto Optimal points regarding the high score and the similarity to TCNQ molecule, which is well-known for the interesting electronic properties as a co-crystal co-former.[18] A point is regarded as Pareto optimal in cases where there is no other point such that the desired objectives are improved simultaneously, *i.e.* both score and structural similarity to TCNQ are maximized.

Table S14. Pareto ranking when optimizing 5 parameters as acquired from Pipeline Pilot. (optimized parameters: Price -> maximized, number of_cocrystal -> minimized, Tamimoto similarity to known co-formers with pyrene -> minimized, tanimoto similarity to TCNQ -> maximized). The calculation was performed only for the co-formers with prices less than £200/1g and we are focucing on those with zero number of reported co-crystals. It can be observed that the five molecules we attempted to use in synthetic work are in ParetoFront 1 and 2. The molecules that were screened experimentally (1-6) are highlighted with bold.

smiles	price(/1g)	number_of_cocrystals	scores	tanimoto to known pyrene coformers	distance to TCNQ	Pareto Front	Crowding Distance
**c1ccc(cc1)c2c3ccccc3cc4ccccc24 (4)**	21	0	0.79273593	0.393939394	0.106382979	1	1E+99
c1ccc(cc1)P(c2ccccc2)c3ccc4ccccc4c3c5c(ccc6ccccc56)P(c7ccccc7)c8ccccc8	17	0	0.7981133	0.301204819	0.058139535	1	1E+99
**N#Cc1c2ccccc2c(C#N)c3ccccc13 (2)**	35	0	0.72066504	0.265306122	0.2	1	1E+99
S=C=Nc1cccc2ccccc12	30.9	0	0.3786586	0.195876289	0.154929577	1	1E+99
o1ccc2ccccc12	4.18	0	0.7044418	0.175675676	0.14516129	1	1E+99
C1Cc2ccccc12	129	0	0.7443961	0.15	0.133333333	1	1E+99
CN(Cc1ccc(cc1)C(C)(C)C)Cc2cccc3ccccc23	83	0	0.7984278	0.247863248	0.098039216	1	2.823559
O=C1c2ccccc2C=Cc3ccccc13	0.99	0	0.7741672	0.302083333	0.12345679	1	1.477933
C=Cc1ccc2ccccc2c1	85.2	0	0.83732426	0.189473684	0.144927536	1	1.296141
Cc1cccc2cccc(C)c12	72.9	0	0.78758216	0.236842105	0.147058824	1	1.153976

COc1ccc2ccccc2c1	0.2	0	0.77328277	0.189473684	0.144927536	1	0.272085
[C-]#[N+]c1ccc2ccccc2c1	65.4	0	0.6639398	0.189473684	0.144927536	2	1E+99
Cc1c2ccccc2c(C)c3c1ccc4ccccc34	82	0	0.7849483	0.307692308	0.108695652	2	3.953052
**O=C1Oc2ccccc2c3ccccc13 (1)**	120	0	0.8051945	0.257731959	0.128205128	2	1.710217
**O=C1CCCc2ccc3ccccc3c12 (3)**	120	0	0.78516996	0.238095238	0.128205128	2	1.053558
[O-][N+](=O)c1ccc2ccccc2c1	56	0	0.64138496	0.276315789	0.14084507	2	0.80574
Cc1cc2ccccc2cc1C	48.7	0	0.7717488	0.196428571	0.114285714	2	0.622167
**C=Cc1cccc2ccccc12 (5)**	67.1	0	0.75620985	0.202531646	0.144927536	2	0.574148
CCOC(=O)Cc1cccc2ccccc12	3	0	0.73698723	0.191011236	0.139240506	2	0.535242
Cc1nccc2ccccc12	24.8	0	0.72139645	0.194444444	0.134328358	3	1E+99
CS(=O)(=O)c1ccccc1	5.68	0	0.60952055	0.227272727	0.109375	3	1E+99
O=C1C(=O)c2c3ccccc3cc4cccc1c24	59.2	0	0.7254807	0.365853659	0.11627907	3	3.64843
O(c1ccccc1)c2cccc(Oc3ccccc3)c2	7.1	0	0.7290225	0.339805825	0.106382979	3	0.860812
C1CC1c2cccc3ccccc23	40	0	0.6346386	0.195876289	0.138888889	3	0.795675
O=C1C=C(Oc2c1ccc3ccccc23)c4ccccc4	12	0	0.62570065	0.320754717	0.104166667	3	0.766259
[O-][N+](=O)c1c2ccccc2cc3ccccc13	14.9	0	0.67084086	0.26744186	0.120481928	3	0.761211
CC(=O)c1ccc2cc3ccccc3cc2c1	80.8	0	0.686586	0.20952381	0.134146341	4	1E+99
O=C1CCc2c(O1)ccc3ccccc23	196	0	0.7204851	0.223880597	0.128205128	4	1E+99
C[n+]1c2ccccc2c(c3c4ccccc4[n+](C)c5ccccc35)c6ccccc16	67	0	0.561863	0.431034483	0.081967213	4	1E+99

[O-][N+](=O)c1cc(c2ccccc2c1)[N+](=O)[O-]	50.2	0	0.5639656	0.440677966	0.1	4	1E+99
Cc1ccccc1	0.05	1	0.8746085	0.193548387	0.122807018	1	1E+99
o1ncc2ccccc12	10.94	1	0.46706015	0.144736842	0.126984127	1	1E+99
Clc1ccccc1	0.03	1	0.71085477	0.157894737	0.122807018	1	1E+99
Clc1ccccc1Cl	0.03	1	0.6358253	0.212765957	0.137931034	1	2.925607
CC(=O)c1ccc2ccccc2c1	3.2	1	0.8166031	0.197530864	0.157142857	1	2.469127
COc1ccccc1	0.05	1	0.5714836	0.147727273	0.116666667	1	1.327223
c1ccc2cc3ccccc3cc2c1	13	1	0.85548186	0.276595745	0.131578947	1	1.029108
Fc1ccccc1F	1.77	1	0.55542886	0.157894737	0.137931034	1	0.910736
Clc1cccc2ccccc12	0.19	1	0.67669845	0.211267606	0.151515152	1	0.828215
Brc1ccccc1	65.5	1	0.70618486	0.152941176	0.122807018	1	0.809937
c1ccc(cc1)c2ccccc2	0.56	1	0.8196181	0.295454545	0.142857143	1	0.780638
Ic1ccccc1	0.48	1	0.6327518	0.152941176	0.122807018	1	0.758601
Cc1ccc2ccccc2c1	0.15	1	0.755266	0.246376812	0.151515152	1	0.482223
Cc1ccccc1C	24.4	1	0.7887064	0.157894737	0.137931034	1	0.41945
Brc1cccc(Br)c1	22.18	1	0.80622596	0.195652174	0.137931034	1	0.207208
c1ccc(cc1)C#Cc2c3ccccc3c(C#Cc4ccccc4)c5ccccc25	25	1	0.8624107	0.4	0.116666667	2	1E+99
c1cc2cccc3c4cccc5cccc(c(c1)c23)c45	44	1	0.8162625	0.301886792	0.106382979	2	1E+99
c1ccc2cc3cc4ccccc4cc3cc2c1	192	1	0.83921814	0.294117647	0.113636364	2	1E+99
O=S(=O)(c1ccccc1)c2ccccc2	0.16	1	0.5948882	0.273684211	0.12987013	2	1E+99
c1ccc2ccccc2c1	25.4	1	0.74946034	0.255813953	0.15625	2	1E+99
Fc1ccccc1	2.32	1	0.534221	0.152941176	0.122807018	2	1E+99
[O-][N+](=O)c1cccc2ccccc12	0.29	1	0.67537975	0.347222222	0.14084507	2	3.03596
Fc1cccc2ccccc12	4	1	0.57493293	0.210526316	0.151515152	2	1.19521
C1CCC(CC1)c2cccc3ccccc23	37.63	1	0.70464814	0.177570093	0.12195122	2	0.962084
c1ccc2cnncc2c1	10	1	0.59604543	0.153846154	0.121212121	2	0.917912
Cc1ccc(C)c2ccccc12	15.28	1	0.7854327	0.222222222	0.147058824	2	0.776378

c1ccc2c(c1)ccc3ccc4ccccc4c23	61.69	1	0.81538904	0.346938776	0.113636364	2	0.737554
c1ccc2c(c1)ccc3ccccc23	30	1	0.8118669	0.304347826	0.131578947	2	0.711468
c1ccc2cccc2cc1	73.7	1	0.6458365	0.227272727	0.15625	2	0.654148
C1=Cc2cccc3cccc1c23	10	1	0.80281925	0.230769231	0.142857143	2	0.554704
o1c2ccccc2c3ccccc13	0.72	1	0.6466993	0.258064516	0.136986301	2	0.510453
O(c1ccccc1)c2ccccc2	0.495	1	0.69433314	0.285714286	0.136986301	2	0.45795
Brc1cnc2ccccc2c1	2	1	0.65355504	0.181818182	0.117647059	2	0.457255
Brc1ccccc1Br	1	1	0.65333426	0.222222222	0.137931034	2	0.320085
O=C1c2ccccc2Oc3ccccc13	0.8	1	0.7725264	0.257731959	0.128205128	2	0.190267
O=C1C(=C2C(=C1c3ccccc3)c4ccccc4c5ccccc25)c6ccccc6	29.1	1	0.84744203	0.452173913	0.081300813	3	1E+99
c1ccc(cc1)c2c3ccccc3c(c4ccccc4)c5ccccc25	47.5	1	0.8568815	0.444444444	0.089285714	3	1E+99
c1ccc2c(c1)c3cccc4cccc2c34	1.08	1	0.6798372	0.431818182	0.12195122	3	1E+99
c1ccc2cc3cc4cc5ccccc5cc4cc3cc2c1	189	1	0.8038188	0.309090909	0.1	3	1E+99
c1ccc2c(c1)sc3ccccc23	1.52	1	0.4949149	0.258064516	0.136986301	3	1E+99
Cc1cccc2c(C)cccc12	106.4	1	0.78139895	0.236842105	0.147058824	3	1E+99
c1cc2ccc3ccc4ccc5cccc6c(c1)c2c3c4c56	112	1	0.7856543	0.285714286	0.1	3	2.200523
c1cc2ccc3ccc4ccc5ccc6ccc1c7c6c5c4c3c27	181	1	0.79979837	0.261538462	0.094339623	3	1.898673
c1ccc(cc1)c2ccc(cc2)c3ccccc3	6.4	1	0.7370869	0.375	0.113636364	3	1.067325
Cc1ccc2ccccc2c1C	50.8	1	0.73880076	0.222222222	0.147058824	3	1.059426
O=C1c2ccccc2C(=O)c3cc4ccccc4cc13	28.36	1	0.7645904	0.36	0.108695652	3	0.788438

Cc1cc(C)c2ccccc2c1	11	1	0.7110152	0.205479452	0.114285714	3	0.763774
c1ccc(cc1)c2ccc(cc2)c3ccc(cc3)c4ccccc4	15	1	0.786963	0.363636364	0.094339623	3	0.622031
c1ccc2c(c1)cnc3ccccc23	39	1	0.7358611	0.263157895	0.131578947	3	0.600971
Brc1c2ccccc2c(Br)c3ccccc13	14	1	0.72304547	0.265306122	0.125	3	0.593683
c1ccc2cc3c(ccc4ccccc34)cc2c1	42	1	0.80535185	0.32	0.113636364	3	0.591964
S1c2ccccc2Sc3ccccc13	1.55	1	0.6450059	0.25	0.131578947	3	0.589532
c1ccc2nc3ccccc3cc2c1	5.12	1	0.6871157	0.263157895	0.131578947	3	0.265507
c1ccc2nc3ccccc3nc2c1	6.5	1	0.68423283	0.25	0.131578947	3	0.159964
c1ccc2c(c1)cc3ccc4cccc5ccc2c3c45	175	1	0.75803137	0.314285714	0.106382979	4	1E+99
**c1ccc2c3ccccc3c4ccccc4c2c1 (6)**	35	1	0	0.375	0.113636364	4	1E+99
O(B(c1ccccc1)c2ccccc2)B(c3ccccc3)c4ccccc4	134	1	0	0.382608696	0.086956522	5	1E+99
c1ccc(cc1)[S+](c2ccccc2)c3ccccc3	170	1	0	0.391752577	0.10989011	5	1E+99

7. Experimental Section

Table S15. Crystallographic data for co-crystal **1** and **2**.

	**1**	**2**
Formula	$C_{16}H_{10} \cdot 2(C_{13}H_8O_2)$	$C_{16}H_{10} \cdot C_{16}H_8N_2$
M_W	594.63	430.48
Crystal System	Monoclinic	Triclinic
Space group	$P2_1/c$	$P\bar{1}$
a/Å	8.2950 (5)	7.3505 (4)
b/Å	16.3146(11)	9.1897 (6)
c/Å	21.1379 (18)	17.0347 (11)
α/°	90	94.567 (6)
β/°	91.514 (7)	91.046 (5)
γ/°	90	113.509 (6)
V/Å^3	2859.6 (4)	1050.24 (12)
Z	4	2
Z'	1	1
T/K	100	100
λ/Å	0.71073	0.71073
D_c/g cm^{-3}	1.381	1.361
μ(Mo-K$_\alpha$)/ mm^{-1}	0.09	0.08
Meas. refl.	6525	3952
Obs. refl. [$I>2\sigma(I)$]	4739	2482
θ range for data collection/ °	2.3-27.5	2.4-25.7
$wR(F^2)$	0.331	0.236
$R[F^2 > 2s(F^2)]$	0.114	0.090
S	1.08	1.05
$\Delta\rho_{max,min}$/ eÅ^{-3}	1.53, -0.56	0.45,-0.29
CCDC Deposit. Number	2014577	2014576

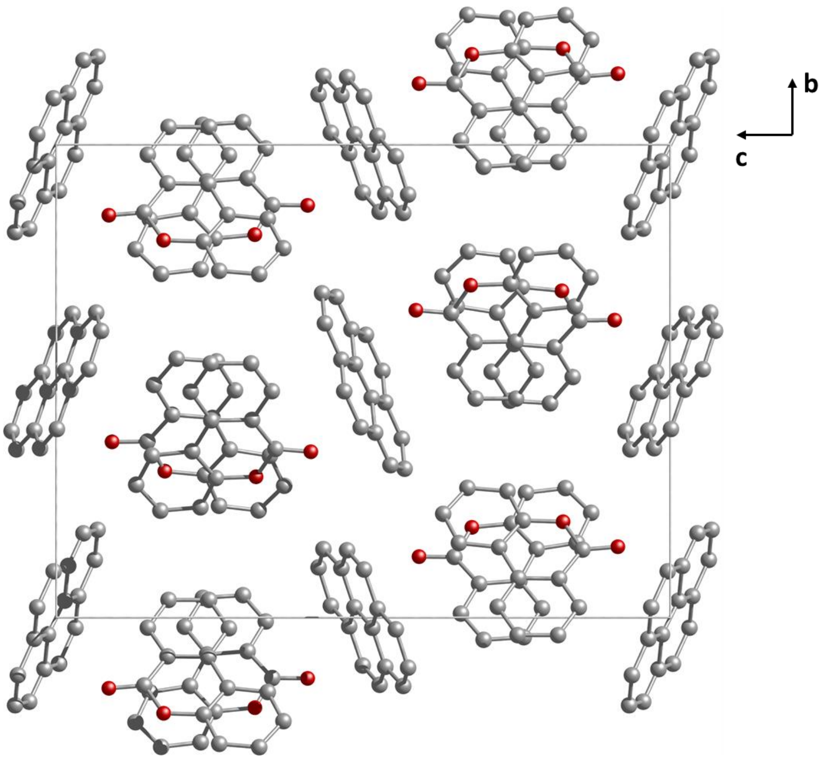

Figure S27. The crystal packing of **1** looking down the *a* axis. Hydrogen atoms are omitted for clarity. O, red; C, grey.

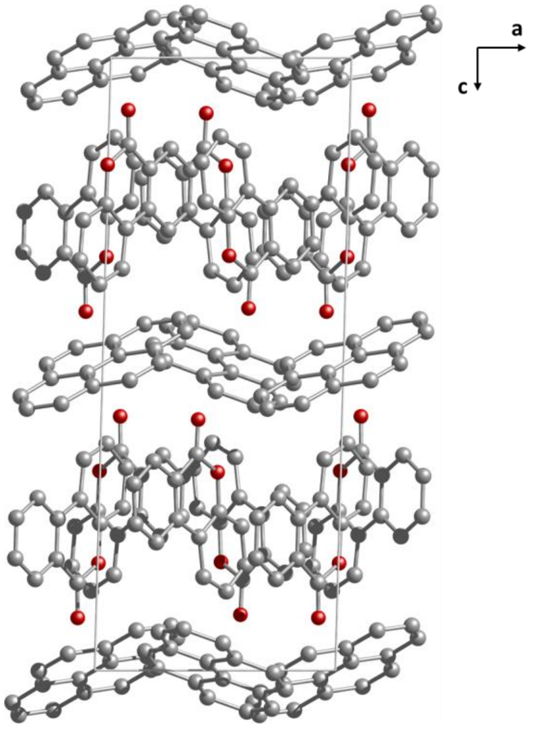

Figure S28. The crystal packing of **1** looking down the *b* axis. Hydrogen atoms are omitted for clarity. O, red; C, grey.

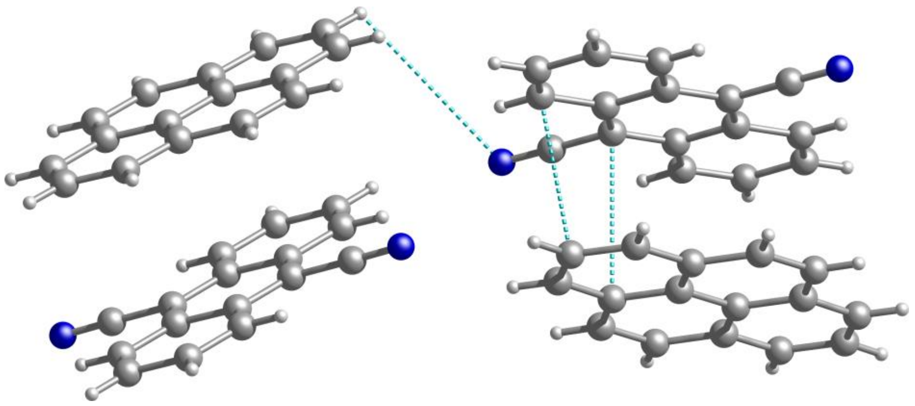

Figure S29. Molecular structure of co-crystal **2** highlighting the π-π and C-H···N interactions**.** Hydrogen atoms are omitted for clarity. N, dark blue; C, grey.

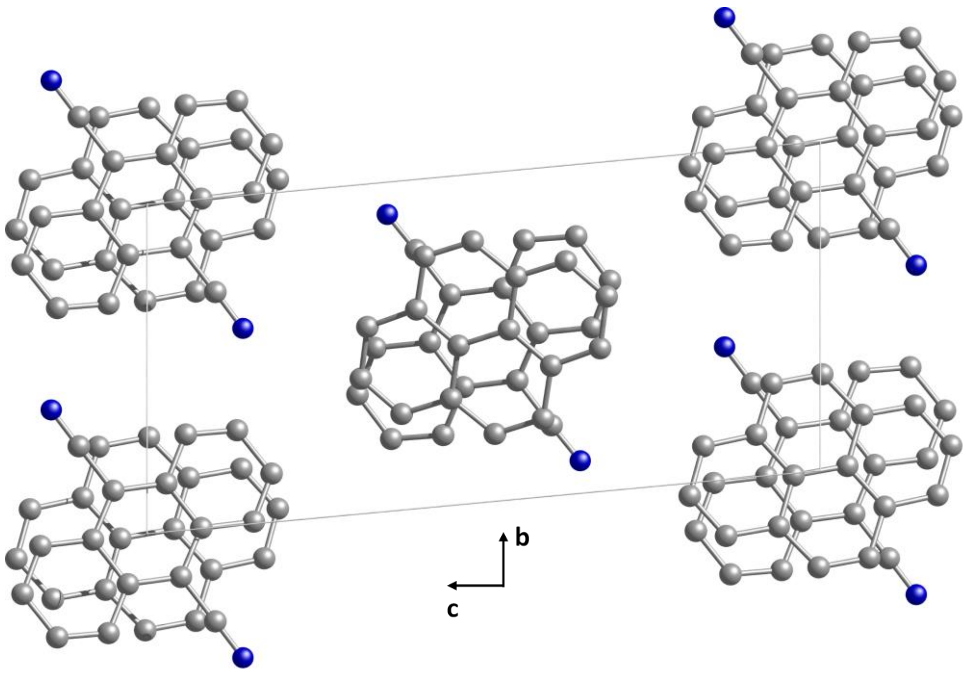

Figure S30. The crystal packing of **2** looking down the *a* axis. Hydrogen atoms are omitted for clarity. N, dark blue; C, grey.

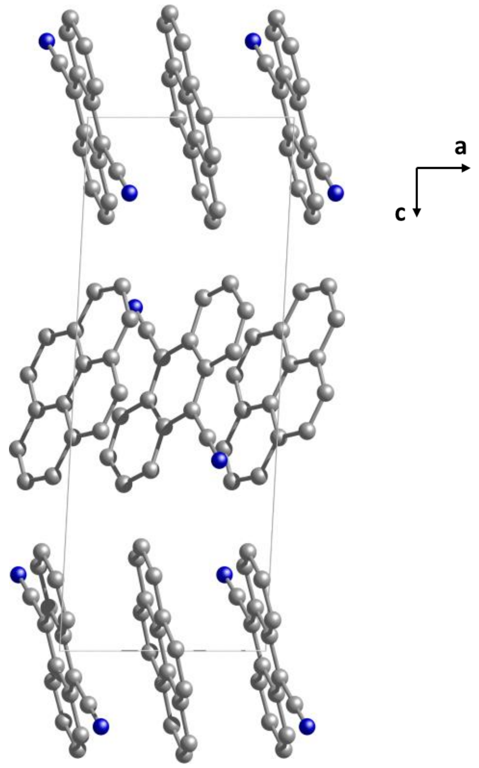

Figure S31. The crystal packing of **2** looking down the *b* axis. Hydrogen atoms are omitted for clarity. N, dark blue; C, grey.

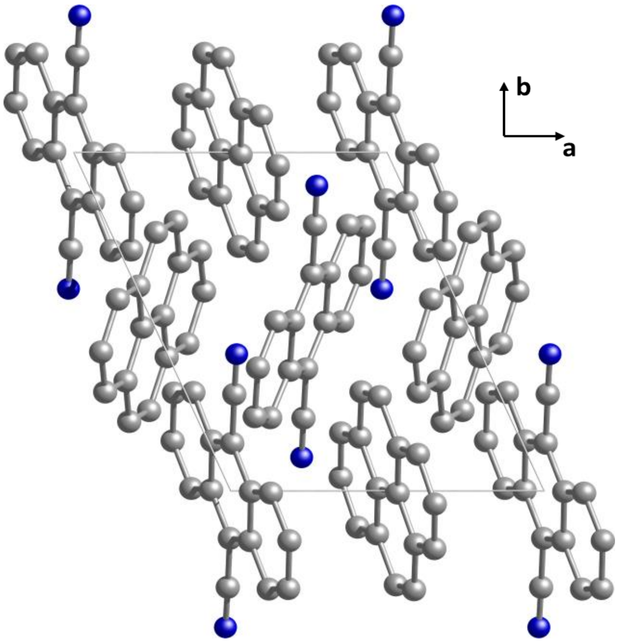

Figure S32. The crystal packing of **2** looking down the *c* axis. Hydrogen atoms are omitted for clarity. N, dark blue; C, grey.

8. Comparison with known CSD co-crystals

8.1 Pyrene-based co-crystals

Cambridge Structural Database (CSD, 2019 release) was investigated in the search for the known pyrene-based co-crystals. The graph of PYRENE entry, including hydrogen atoms, was used as starting query in the ConQuest software. The filters: 3D coordinates determined, not polymeric, no ions and only organics, applied to the results leads to the list reported in Table S16.

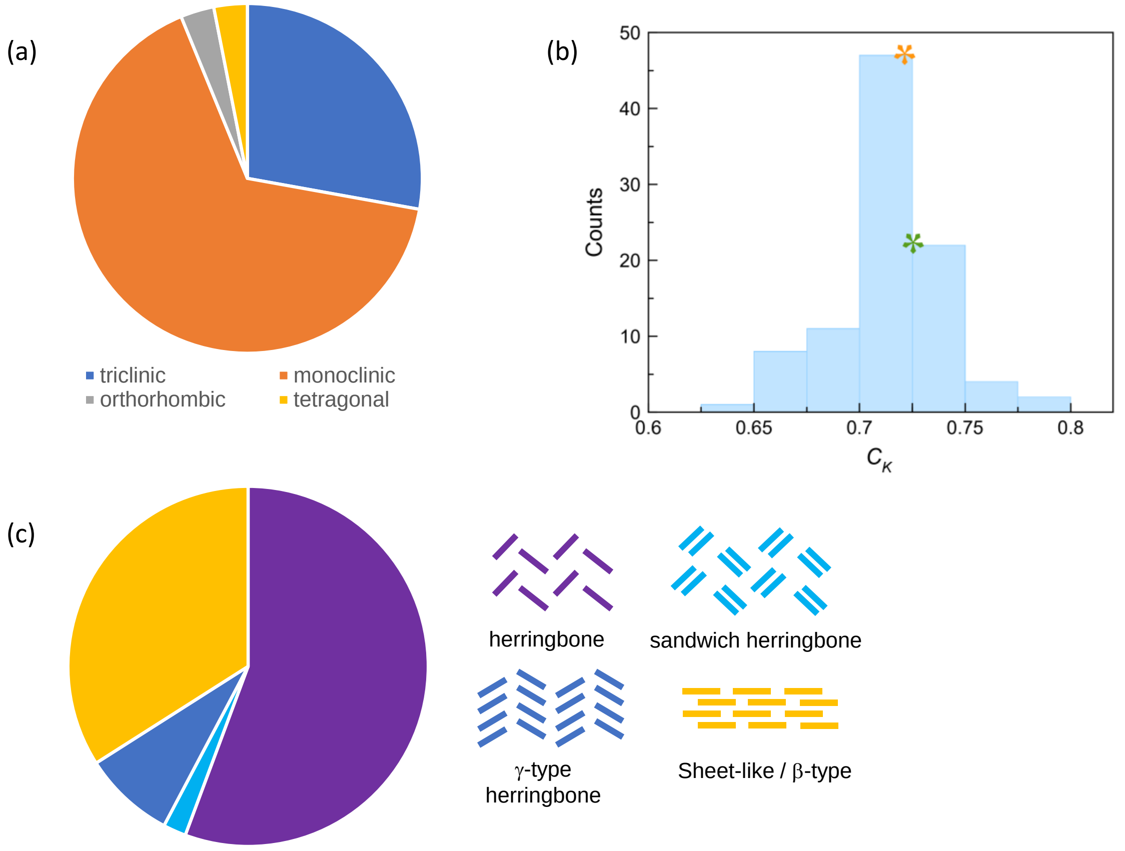

Figure S33. (a) Pie chart of the symmetry system of Pyrene co-crystal reported to literature [CCDC 2019 release, two independent chemical units]. (b) Histogram showing the range of packing coefficient (C_K) of pyrene cocrystal [CCDC 2019 release, two independent chemical units], the orange and green stars refer to **1** and **2** respectively. (c) Pie chart of the different packing types of pyrene co-crystals. Colour code: herringbone, violet; sandwich herringbone, light blue; γ-type herringbone, blue; sheet-like/β-type, yellow.

Table S16. List of the structural parameter of pyrene co-crystal reported in CCDC database (2019 release).

CCDC ref. code	T [K]	Space Group	C_K	*a* [Å]	*b* [Å]	*c* [Å]	α [°]	β [°]	γ [°]	vol [Å³]	Ref
**CUSZUM**	180	*P*1	0.68	9.8401	11.3738	11.4241	115.037	91.454	91.791	1156.817	[19]
**BITBUD**	100	$P\bar{1}$	0.72	7.004	10.09	11.783	107.42	106.46	93.44	752.352	[20]
**ELUGOJ**	110	$P\bar{1}$	0.72	13.8522	15.6089	15.8464	65.532	83.496	89.872	3094.711	[21]
**GUQQEQ**	110	$P\bar{1}$	0.72	9.155	13.793	13.924	91.993	105.843	90.323	1690.229	[22]
**XETTEW**	113	$P\bar{1}$	0.72	8.393	9.7237	12.9654	94.018	91.57	110.732	985.624	[23]
**PINJUU03**	115	$P\bar{1}$	0.73	7.1106	17.278	17.748	62.924	82.368	82.571	1918.431	[24]
**ECUVIH**	120	$P\bar{1}$	0.72	6.725	8.864	9.488	107.51	105.23	106.82	476.902	[25]
**GUQRAN**	150	$P\bar{1}$	0.72	7.046	8.334	8.623	116.29	90.15	102.722	439.92	[22]
**WOQQAX**	150	$P\bar{1}$	0.67	13.5717	15.3754	17.5775	65.787	68.112	82.586	3102.85	[26]
**MUGBAS**	173	$P\bar{1}$	0.75	8.1578	8.203	10.141	89.462	76.889	80.215	651.014	[27]
**EHETEQ**	174	$P\bar{1}$	0.74	7.3295	8.55	19.185	88.15	79.18	87.08	1179.047	[28]
**PINJUU02**	220	$P\bar{1}$	0.72	7.1779	17.415	17.827	62.427	81.939	82.145	1949.264	[24]
**ISISAG**	240	$P\bar{1}$	0.71	7.367	8.555	15.803	94.02	102.77	89.86	968.867	[29]
**GUMNUY**	273	$P\bar{1}$	0.70	7.9341	9.1661	10.3306	89.439	88.443	72.669	716.916	[30]
**UZEGOX**	273	$P\bar{1}$	0.68	8.7758	12.0214	13.3155	66.461	74.489	74.462	1220.151	[31]
**BEFGIC**	295	$P\bar{1}$	0.65	10.085	10.646	11.037	98.73	92.61	107.36	1112.713	[32]
**FETYAE**	295	$P\bar{1}$	0.70	8.046	15.067	16.433	82.03	89.1	87.52	1970.972	[33]
**GAFJAY**	295	$P\bar{1}$	0.69	10.172	13.798	9.302	92.56	117.24	108.8	1069.821	[34]
**PYRTNB**	295	$P\bar{1}$	0.71	6.77	16.35	8.55	93	101.3	95.6	921.141	[35]
**PYTQIM**	295	$P\bar{1}$	0.70	7.393	8.037	20.873	99.6	92.95	95.13	1215.171	[36]

**TEXPOB**	295	$P\bar{1}$	0.73	7.092	8.378	8.664	112.922	92.86	92.078	472.678	[37]
**TEXPOB10**	295	$P\bar{1}$	0.73	7.092	8.378	8.664	112.922	92.86	92.078	472.678	[38]
**XAGMAT**	295	$P\bar{1}$	0.66	9.727	10.854	11.62	106.3	104.11	104.43	1073.432	[39]
**TUYVUF**	298	$P\bar{1}$	0.72	8.4846	11.1538	15.236	69.813	82.882	83.346	1338.721	[40]
**QOLPUF**	298	$P\bar{1}$	0.70	7.457	7.942	11.259	71.93	74.01	89.57	607.163	[41]
**QOLQOA**	298	$P\bar{1}$	0.69	7.317	7.754	11.041	104.81	101.31	91.4	592.011	[41]
**QOLRER**	298	$P\bar{1}$	0.70	7.506	7.856	10.872	69.79	76.82	89.71	583.864	[41]
**MUFZIX**	173	$P2_1$	0.72	14.799	8.197	25.036	90	90.744	90	3036.796	[27]
**REQVOZ**	123	Pc	0.73	8.3157	38.967	14.2436	90	91.718	90	4613.391	[42]
**AYEGAM**	173	Pc	0.70	7.851	7.657	16.296	90	111.09	90	914.016	[43]
**PYRPMA04**	19	$P2_1/n$	0.74	13.664	9.281	14.42	90	91.8	90	1827.778	[44]
**AGORAS01**	100	$P2_1/c$	0.74	14.058	10.1	15.429	90	92.03	90	2189.324	[45]
**AGOREW01**	100	$P2_1/c$	0.73	15.694	10.7983	20.1481	90	90.421	90	3414.377	[45]
**MIDDIP**	100	$P2_1/n$	0.71	8.973	26.857	17.476	90	100.268	90	4144.056	[46]
**PYRTCQ02**	100	$P2_1/n$	0.72	6.9917	10.069	14.671	90	103.52	90	1004.209	[20]
**CENTOH**	103	$P2_1/c$	0.80	7.2231	8.419	19.036	90	95.086	90	1153.046	[47]
**PYRCYE02**	105	$P2_1/a$	0.72	14.136	7.169	7.866	90	91.73	90	796.785	[48]
**PYRCYE03**	105	$P2_1/a$	0.46	14.136	7.169	7.866	90	91.73	90	796.785	[48]
**GUQQAM**	110	$P2_1/c$	0.72	9.1973	13.6331	14.3279	90	112.02	90	1665.49	[22]
**GUQQIU**	110	$P2_1/c$	0.71	12.267	15.636	9.2024	90	97.593	90	1749.606	[22]
**GUQQOA**	110	$P2_1/c$	0.72	11.9762	15.3782	9.7871	90	99.867	90	1775.851	[22]
**GUQQUG**	110	$P2_1/c$	0.72	14.458	8.874	17.339	90	126.716	90	1783.258	[22]

**PYRPMA10**	110	$P2_1/n$	0.71	13.667	9.13	14.404	90	91.5	90	1796.711	[49]
**ZZZGKE02**	110	$P2_1/c$	0.72	6.8822	13.238	9.2058	90	106.261	90	805.157	[22]
**DIZZOD**	120	$P2_1/n$	0.74	7.2226	16.1783	21.334	90	92.461	90	2490.566	[50]
**MOBWEI**	120	$P2_1/n$	0.72	10.7122	18.7549	12.3835	90	91.632	90	2486.913	[51]
**REDCIM01**	150	$P2_1/n$	0.72	10.8636	14.0746	12.4274	90	109.674	90	1789.235	[52]
**WOQPOK**	150	$P2_1/n$	0.69	21.8778	9.0276	25.1726	90	92.949	90	4965.106	[26]
**BORPII**	173	$P2_1/n$	0.72	8.747	6.94	15.327	90	104.356	90	901.36	[53]
**EHESIT**	173	$P2_1/n$	0.70	6.8334	15.809	17.147	90	90.58	90	1852.282	[28]
**EHESUF**	173	$P2_1/n$	0.72	13.516	9.669	14.451	90	99	90	1865.295	[28]
**PYRCBZ02**	173	$P2_1/c$	0.71	7.154	8.4	15.53	90	93.833	90	931.166	[54]
**EHESEP**	174	$P2_1/n$	0.73	6.7858	15.487	17.092	90	91.26	90	1795.793	[28]
**EHESOZ**	174	$P2_1/n$	0.73	6.8295	16.236	17.096	90	96.37	90	1883.965	[28]
**EHETAM**	174	$P2_1/c$	0.72	14.955	17.564	14.329	90	95.93	90	3743.652	[28]
**WAWPAM**	174	$P2_1/n$	0.73	7.0679	15.983	8.907	90	104.78	90	972.898	[55]
**PYRCBZ01**	178	P1121/b	0.68	7.27	15.36	8.38	90	90	94	933.492	[56]
**PYRFLR01**	200	$P2_1/n$	0.74	7.797	6.973	14.723	90	94.84	90	797.613	[57]
**PYRPMA11**	200	$P2_1/c$	0.72	7.268	9.35	13.757	90	92.71	90	933.822	[58]
**REDCIM**	200	$P2_1/n$	0.71	10.893	14.114	12.49	90	109.53	90	1809.781	[59]
**REDFIP**	200	$P2_1/c$	0.74	7.469	9.007	13.853	90	96.17	90	926.538	[57]
**ZZZGKE01**	200	$P2_1/c$	0.71	6.9467	13.331	9.301	90	106.67	90	825.133	[60]
**PINJUU01**	230	$P2_1/n$	0.75	7.1751	9.1122	15.1404	90	99.0425	90	977.591	[24]
**AGOREW**	100	C2/c	0.73	24.36	10.9124	15.583	90	124.426	90	3416.861	[45]

**PAYYOG**	200	C2/c	0.72	12.019	20.022	8.703	90	99.99	90	2062.574	[58]
**PYRPCT02**	293	*Pc*	0.74	17.303	6.6434	16.85	90	110.791	90	1810.791	[61]
**MURPYR**	295	*Pc*	0.64	9.71	8	15.04	90	117	90	1040.969	[62]
**PYRCBZ**	290	$P112_1/b$	0.66	7.27	15.57	8.44	90	90	93.6	953.471	[56]
**FARNIX**	293	$P2_1/c$	0.69	10.277	15.593	9.715	90	114.844	90	1412.746	[63]
**QEVXOH**	293	$P2_1/c$	0.67	7.8395	14.7083	17.2969	90	102.132	90	1949.888	[64]
**BAZCUA**	295	$P2_1/c$	0.71	10.057	7.86	15.168	90	106.35	90	1150.513	[65]
**BAZDAH**	295	$P2_1/c$	0.72	9.9	7.833	14.929	90	106.72	90	1108.75	[65]
**CEKBUP**	295	$P2_1/a$	0.73	10.536	12.877	7.314	90	114.1	90	905.81	[66]
**CILRAQ**	295	$P2_1/n$	0.71	10.633	16.336	11.683	90	94.62	90	2022.751	[67]
**PYRBPC**	295	$P2_1/c$	0.70	8.189	21.07	14.607	90	91.7	90	2519.215	[68]
**PYRCLN**	295	$P112_1/b$	0.74	7.52	13.68	8.93	90	90	96.5	912.756	[69]
**PYRCYE10**	295	$P2_1/a$	0.71	14.333	7.242	7.978	90	92.36	90	827.411	[70]
**PYRFLR**	295	$P2_1/a$	0.73	17.308	7.066	7.825	90	121.82	90	813.158	[71]
**PYRPMA02**	295	$P2_1/a$	0.71	13.885	9.303	7.307	90	93.5	90	942.1	[72]
**PYRPMA03**	295	$P2_1/a$	0.66	13.885	9.303	7.307	90	93.5	90	30942.1	[72]
**PYRTCQ**	295	$P112_1/b$	0.71	7.14	14.73	10.01	90	90	102.5	1027.819	[73]
**FARNOD**	296	$P2_1/c$	0.67	8.3885	18.3017	13.1838	90	105.268	90	1952.588	[63]
**MIDDEL**	296	$P2_1/n$	0.74	6.5612	18.654	8.5145	90	99.983	90	1026.334	[74]
**OPUQUN**	296	$P2_1/c$	0.51	7.609	25.637	11.675	90	101.03	90	2235.394	[75]
**PYRTCQ03**	296	$P2_1/n$	0.71	6.9996	10.0807	14.6724	90	103.567	90	1006.409	[76]
**QOLQEQ**	298	$P2_1/n$	0.72	7.564	7.574	22.841	90	96.9	90	1299.077	[41]

**PINJUU**	300	$P2_1/n$	0.78	7.2509	9.1166	15.2982	90	99.659	90	996.929	[24]
**PYRPMA01**	300	$P2_1/a$	0.68	13.89	9.33	7.34	90	93.5	90	949.444	[77]
**AGOREW02**	338	$P2_1/c$	0.69	15.653	11.046	20.774	90	91.3	90	3590.963	[45]
**CUNWUD**	295	$C2/c$	0.68	17.846	15.449	16.27	90	95.59	90	4464.353	[78]
**HAYYOW**	296	$C2/c$	0.71	12.032	15.808	10.673	90	103.66	90	1972.604	[79]
**CUTBEZ**	180	$P2_12_12_1$	0.67	7.509	17.9935	19.0703	90	90	90	2576.649	[19]
**AGORAS**	100	$Pca2_1$	0.73	20.145	7.169	15.362	90	90	90	2218.572	[45]
**QEVWEW**	285	$Pbcn$	0.68	5.1221	17.608	22.7903	90	90	90	2055.456	[64]
**PYRBZQ01**	100	$P4_3$	0.75	7.5953	7.5953	25.2629	90	90	90	1457.381	[80]
**CORPIJ**	130	$P4_3$	0.75	7.5714	7.5714	26.8898	90	90	90	1541.487	[80]
**PYRBZQ**	295	$P4_1$	0.73	7.698	7.698	25.57	90	90	90	1515.258	[81]

8.2 UMAP projection of the co-crystals space

UMAP (Uniform Manifold Approximation and Projection for Dimension Reduction)[82] was implemented for a low-dimensional space encoding of the labelled dataset. Each point on the UMAP visualization is coloured according to the difference of the molecular descriptors. All the descriptors are normalized to [0,1] to be comparable. The implemented UMAP settings were selected based on the best distance preservation between the high dimensions and the two-dimensional embeddings. The distance preservation was measured by calculating the Pearson correlation coefficient of the distance matrix using the whole dimensionality and the distance matrix after the dimensionality reduction. The most effective settings were as follows (n_neighbours = 80, min_dist = 0.1, euclidean distance metric) resulting in Pearson correlation coefficient of 0.748.

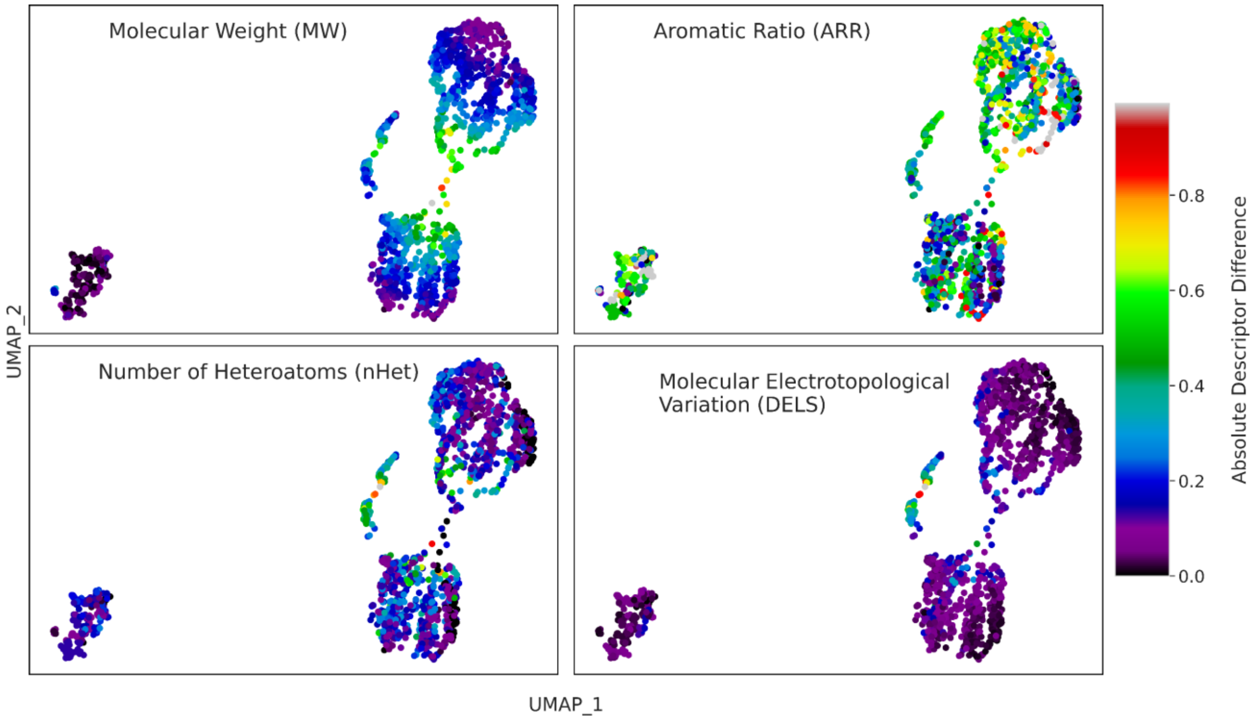

Figure S34. UMAP 2D projection showing the distribution of selected molecular descriptors across the co-crystal space. It can be observed that not all the descriptors show similar trends across the molecular pairs map.

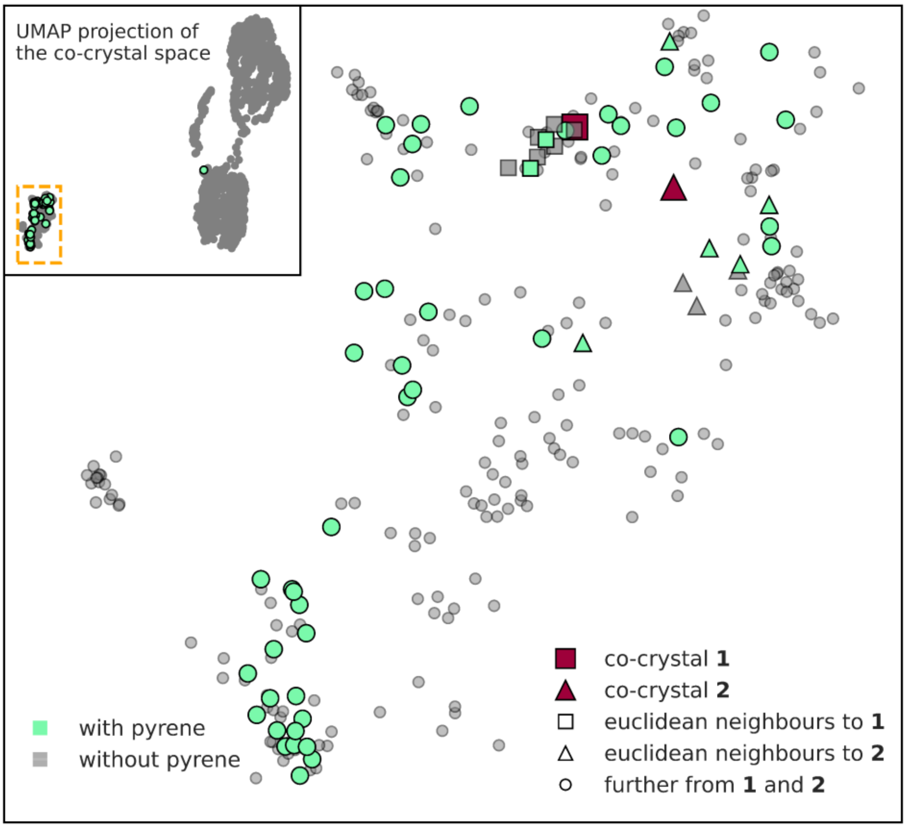

Figure S35. UMAP 2D visualization of the overall co-crystal dataset (inset) and zoomed view of the hightlighted cluster. **1** and **2** are represented with red square and triangle respectively. The closest neighbours to **1**, as calculated by the Euclidean distance of the descriptors, are visualized with smaller squares, whereas the closest neighbours to **2** with smaller triangles. The light green and grey color codes stand for molecular pairs containing pyrene and those without pyrene respectively. Intrestingly the majority of the pyrene co-crystals belong to the same cluster formed by molecules with similar characteristics. It was observed that even though **1** and **2** are quite similar feature-wise to known pyrene co-crystals, the crystal packing both of them adopt, (*i.e.*, the γ motif) was rare and more complex.

8.3 Comparison with known structures

The synthesized co-crystals **1** and **2** were compared to the known co-crystals consisting the labelled dataset. The comparison was performed using all the available molecular descriptors acquired from Dragon software.[83] As before, each molecular pair is represented by the concatenation of the molecular descriptors of each molecule in the pair. The distance between **1** and **2** and the known CSD structures is calculated by measuring the Euclidean distance of the vectors of the two new structures to the vectors of the labelled dataset.

The Euclidean Distance between two points p.q in **n** dimensional space is defined as:[84]

$$d(p,q) = \sqrt{(p_1 - q_1)^2 + (p_2 - q_2)^2 + \cdots + (p_3 - q_3)^2 + \cdots + (p_n - q_n)^2} = \sqrt{\sum_{i=1}^{n}(p_i - q_i)^2} \quad (3)$$

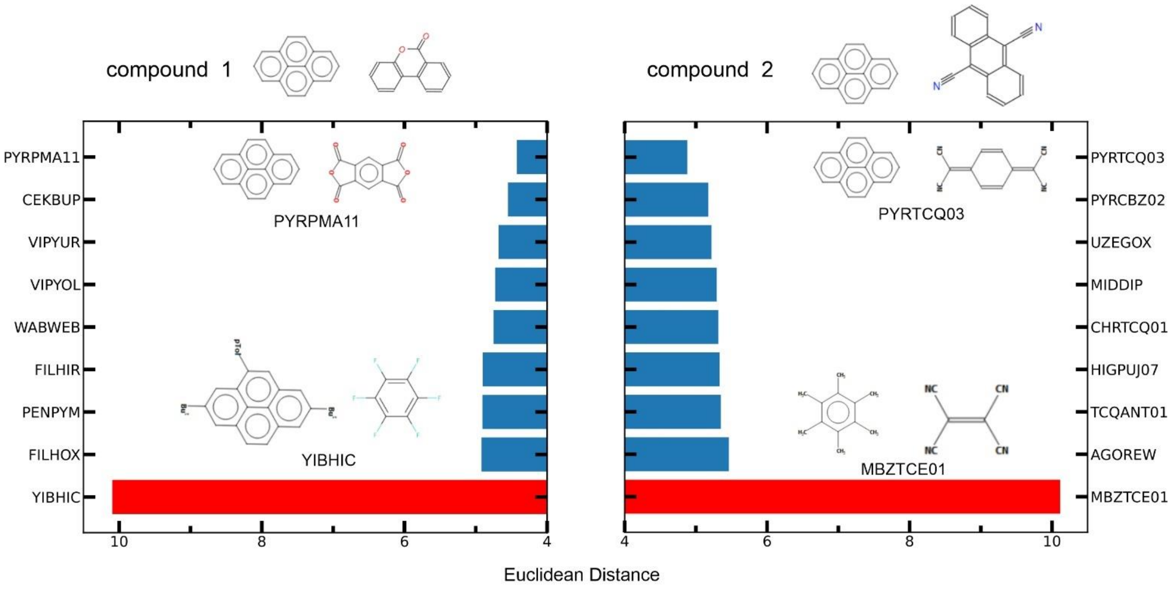

Figure S36. Euclidean distance of **1** and **2** to the closest known co-crystals (blue bars) of the labelled dataset. The red bar represents a more distant co-crystal for comparison purposes.

Table S17. List of the significant structural motifs and of the crystal packing coefficients (C_k) of **1** and of the most similar co-crystals in CSD database in terms of Euclidean distances.

	Co-formers ratio	π-π [Å]	C-H···π [Å]	C-H···O [Å]	C_k	Ref
**Co-crystal 1**	1:2	3.34-3.35	2.72-2.87	2.51-2.56	0.72	This work
**PYRPMA11**	1:1	3.34-3.39	-	2.473-2.667	0.72	[58]
**CEKBUP**	1:1	3.20-3.38	-	2.565-2.595	0.73	[66]
**VIPYUR**	1:1	3.31-3.38	-	2.551-2.667	0.74	[85]
**VIPYOL**	1:1	3.31-3.37	-	2.540-2.713	0.71	[85]
**WABWEB**	1:1	3.50-3.60	-	2.514-2.638	0.73	[86]
**FILHIR**	1:1	3.34-3.37	2.856	2.609	0.70	[87]
**PENPYM**	1:1	3.36-3.37	-	2.591-2.712	0.69	[88]
**FILHOX**	1:1	3.36-3.38	-	2.470	0.73	[87]

Table S18. List of the significant structural motifs and of the crystal packing coefficients (C_k) of **2** and of the most similar co-crystals in CSD database in terms of Euclidean distances.

	Co-formers ratio	π-π [Å]	C-H···π [Å]	C-H···N/O* [Å]	C_k	Ref
**Co-crystal 2**	1:1	3.67	-	2.57-2.65	0.73	This work
**PYRTCQ03**	1:1	3.50	-	2.571-2.693	0.71	[76]
**PYRCBZ02**	1:1	3.36	-	2.584	0.71	[54]
**UZEGOX**	1:1	-	2.834	2.604	0.68	[31]
**MIDDIP**	1:1	3.34-3.39	-	2.691-2.737	0.71	[46]
**CHRTCQ01**	1:1	3.34-3.39	-	2.664-2.729	0.75	[20]
**HIGPUJ07**	1:1	3.67	-	2.714	0.70	[89]
**TCQANT03**	1:1	3.506	-	2.672-2.715	0.73	[90]
**AGOREW**	1:1	3.397	-	2.401-2.602(*)	0.73	[45]

SI References:

1 I. R. Thomas, I. J. Bruno, J. C. Cole, C. F. Macrae, E. Pidcock and P. A. Wood, *J. Appl. Cryst*, 2010, **43**, 362–366.

2 T. Sterling and J. J. Irwin, *J. Chem. Inf. Model.*, 2015, **55**, 2324–2337.

3 D. Rogers and M. Hahn, *J. Chem. Inf. Model.*, 2010, **50**, 742–754.

4 Pipeline Pilot. http://accelrys.com/products/pipeline-pilot/.

5 Daylight Theory: SMARTS - A Language for Describing Molecular Patterns, https://www.daylight.com/dayhtml/doc/theory/theory.smarts.html, (accessed 15 June 2020).

6 S. Papadimitriou, H. Kitagawa, P. B. Gibbons and C. Faloutsos, *Proc. 19th Int. Conf. Data Eng. (Cat. No.03CH37405)*, 2003, 315–326.

7 M. M. Breunig, H.-P. Kriegel, R. T. Ng and J. Sander, in *Proceedings of the 2000 ACM SIGMOD international conference on Management of data - SIGMOD '00*, ACM Press, New York, New York, USA, 2000, pp. 93–104.

8 S. Ramaswamy, R. Rastogi and K. Shim, Association for Computing Machinery (ACM), 2000, pp. 427–438.

9 Z. He, X. Xu and S. Deng, *Pattern Recognit. Lett.*, 2003, **24**, 1641–1650.

10 S. S. Khan and M. G. Madden, *Knowl. Eng. Rev.*, 2014, **29**, 345–374.

11 M. Goldstein and A. Dengel, in *In: Wölfl S, editor. KI-2012: Poster and Demo Track. Online*, 2012, pp. 59–63.

12 F. T. Liu, K. M. Ting and Z. H. Zhou, in *Proceedings - IEEE International Conference on Data Mining, ICDM*, 2008, pp. 413–422.

13 A. Lazarevic and V. Kumar, in *Proceedings of the ACM SIGKDD International Conference on Knowledge Discovery and Data Mining*, ACM Press, New York, New York, USA, 2005, pp. 157–166.

14 R. Winter, F. Montanari, F. Noé and D. A. Clevert, *Chem. Sci.*, 2019, **10**, 1692–1701.

15 L. Ruff, R. A. Vandermeulen, N. Görnitz, L. Deecke, S. A. Siddiqui, A. Binder, E. Müller and M. Kloft, in *35th International Conference on Machine Learning, ICML 2018*, International Machine Learning Society (IMLS), 2018, vol. 10, pp. 6981–6996.

16 J. Lee, Y. Lee, J. Kim, A. R. Kosiorek, S. Choi and Y. W. Teh, Proceedings of the 36th International Conference on Machine Learning, 2019.

17 A. V. Stachulski, C. Pidathala, E. C. Row, R. Sharma, N. G. Berry, M. Iqbal, J. Bentley, S. A. Allman, G. Edwards, A. Helm, J. Hellier, B. E. Korba, J. E. Semple and J. F. Rossignol, *J. Med. Chem.*, 2011, **54**, 4119–4132.

18 F. Häse, L. M. Roch and A. Aspuru-Guzik, *Chem. Sci.*, 2018, **9**, 7642–7655.

19 T. Friščić, R. W. Lancaster, L. Fábián and P. G. Karamertzanis, *Proc. Natl. Acad. Sci.*, 2010, **107**, 13216 LP – 13221.

20 M. A. Dobrowolski, G. Garbarino, M. Mezouar, A. Ciesielski and M. K. Cyrański, *CrystEngComm*, 2014, **16**, 415–429.

21 S. Roy, H. M. Titi and I. Goldberg, *CrystEngComm*, 2016, **18**, 3372–3382.

22 X. Pang, H. Wang, W. Wang and W. J. Jin, *Cryst. Growth Des.*, 2015, **15**, 4938–4945.

23 E. Curtis, L. R. Nassimbeni, H. Su and J. H. Taljaard, *Cryst. Growth Des.*, 2006, **6**, 2716–2719.

24 J. Harada, N. Yoneyama, S. Sato, Y. Takahashi and T. Inabe, *Cryst. Growth Des.*, 2019, **19**, 291–299.

25 J. C. Collings, K. P. Roscoe, R. L. Thomas, A. S. Batsanov, L. M. Stimson, J. A. K. Howard and T. B. Marder, *New J. Chem.*, 2001, **25**, 1410–1417.

26 Q. Huang, W. Li, Z. Mao, L. Qu, Y. Li, H. Zhang, T. Yu, Z. Yang, J. Zhao, Y. Zhang, M. P. Aldred and Z. Chi, *Nat. Commun.*, 2019, **10**, 3074.

27 Y. P. Nizhnik, J. Lu, S. V Rosokha and J. K. Kochi, *New J. Chem.*, 2009, **33**, 2317–2325.

28 D. Britton, W. E. Noland, M. J. Pinnow and V. G. Young Jr., *Helv. Chim. Acta*, 2003, **86**, 1175–1192.

29 B. Landeros-Rivera, R. Moreno-Esparza and J. Hernández-Trujillo, *RSC Adv.*, 2016, **6**, 77301–77309.

30 Y. Fujiki, S. Shinkai and K. Sada, *Cryst. Growth Des.*, 2009, **9**, 2751–2755.

31 Y. Ren, S. Lee, J. Bertke, D. L. Gray and J. S. Moore, *Acta Crystallogr. Sect. C*, 2016, **72**, 923–931.

32 R. Doherty, J. M. Stewart, A. D. Mighell, C. R. Hubbard and A. J. Fatiadi, *Acta Crystallogr. Sect. B*, 1982, **38**, 859–863.

33 J. C. Barnes and W. Golnazarians, *Acta Crystallogr. Sect. C*, 1987, **43**, 549–552.

34 C. Kabuto, Y. Fukazawa, T. Suzuki, Y. Yamashita, T. Miyashi and T. Mukai, *Tetrahedron Lett.*, 1986, **27**, 925–928.

35 C. K. Prout and I. J. Tickle, *J. Chem. Soc. Perkin Trans. 2*, 1973, 734–737.

36 A. E. Shvets, Y. Y. Bleidelis, E. Y. Markava, Y. F. Freimanis and D. V. Kanepe, *Zh.Strukt.Khim.*, 1980, **21**, 190.

37 H. Bock, W. Seitz, M. Sievert, M. Kleine and J. W. Bats, *Angew. Chemie Int. Ed. English*, 1996, **35**, 2244–2246.

38 H. Bock, W. Seitz, M. Sievert, M. Kleine and J. W. Bats, *Liebigs Ann.*, 1996, **1996**, 1929–1940.

39 J. Blömker and W. Frey, *Zeitschrift für Krist. - New Cryst. Struct.*, 2000, **215**, 263–264.

40 J. N. Moorthy, P. Natarajan and P. Venugopalan, *J. Org. Chem.*, 2009, **74**, 8566–8577.

41 S. Fan, Y. Kiyota, K. Iijima, S. Ryo, T. Kawamoto, Y. Le Gal, D. Lorcy and T. Mori, *CrystEngComm*, 2019, **21**, 5227–5234.

42 S. V Rosokha, S. M. Dibrov, T. Y. Rosokha and J. K. Kochi, *Photochem. Photobiol. Sci.*, 2006, **5**, 914–924.

43 D. Britton, *Acta Crystallogr. Sect. E*, 2004, **60**, o1117–o1118.

44 F. H. Herbstein, R. E. Marsh and S. Samson, *Acta Crystallogr. Sect. B*, 1994, **50**, 174–181.

45 M. Singh and D. Chopra, *Cryst. Growth Des.*, 2018, **18**, 6670–6680.

46 Q. Zhang, *CSD Commun.*, CCDC 1845459: Experimental Crystal Structure Determination, 2018, DOI: 10.5517/ccdc.csd.cc1zyby6.

47 P. Hu, S. Wang, A. Chaturvedi, F. Wei, X. Zhu, X. Zhang, R. Li, Y. Li, H. Jiang, Y. Long and C. Kloc, *Cryst. Growth Des.*, 2018, **18**, 1776–1785.

48 F. K. Larsen, R. G. Little and P. Coppens, *Acta Crystallogr. Sect. B*, 1975, **31**, 430–440.

49 F. H. Herbstein and J. A. Snyman, *Philos.Trans.R.Soc.London,Ser.A*, 1969, **264**, 635.

50 T. T. Clikeman, E. V Bukovsky, I. V Kuvychko, L. K. San, S. H. M. Deng, X.-B. Wang, Y.-S. Chen, S. H. Strauss and O. V Boltalina, *Chem. Commun.*, 2014, **50**, 6263–6266.

51 N. J. DeWeerd, E. V Bukovsky, K. P. Castro, I. V Kuvychko, A. A. Popov, S. H. Strauss and O. V Boltalina, *J. Fluor. Chem.*, 2019, **221**, 1–7.

52 R. Vaiyapuri, B. W. Greenland, J. M. Elliott, W. Hayes, R. A. Bennett, C. J. Cardin, H. M. Colquhoun, H. Etman and C. A. Murray, *Anal. Chem.*, 2011, **83**, 6208–6214.

53 S. V Rosokha, J. Lu, B. Han and J. K. Kochi, *New J. Chem.*, 2009, **33**, 545–553.

54 S. Varughese, M. S. R. N. Kiran, U. Ramamurty and G. R. Desiraju, *Chem. – An Asian J.*, 2012, **7**, 2118–2125.

55 D. Britton, *Acta Crystallogr. Sect. C*, 2005, **61**, o662–o664.

56 C. K. Prout, T. Morley, I. J. Tickle and J. D. Wright, *J. Chem. Soc. Perkin Trans. 2*, 1973, 523–527.

57 H. Bock, M. Sievert, H. Schodel and M. Kleine, *Zeitschrift fur Naturforschung, B Chem. Sci.*, 1996, **51**, 1521.

58 K. Kato, S. Hagi, M. Hinoshita, E. Shikoh and Y. Teki, *Phys. Chem. Chem. Phys.*, 2017, **19**, 18845–18853.

59 H. Bock, K. Ziemer, C. Nather, H. Schodel, M. Kleine and M. Sievert, *Zeitschrift fur Naturforschung, B Chem. Sci.*, 1996, **51**, 1538.

60 J. C. Collings, K. P. Roscoe, E. G. Robins, A. S. Batsanov, L. M. Stimson, J. A. K. Howard, S. J. Clark and T. B. Marder, *New J. Chem.*, 2002, **26**, 1740–1746.

61 U. Neupane and R. N. Rai, *J. Solid State Chem.*, 2018, **268**, 67–74.

62 A. Damiani, E. Giglio, A. Ripamonti, A. M. Liquori and P. De Santis, *Acta Crystallogr.*, 1965, **19**, 340–348.

63 Q. J. Shen, H. Q. Wei, W. S. Zou, H. L. Sun and W. J. Jin, *CrystEngComm*, 2012, **14**, 1010–1015.

64 L. Li, W. X. Wu, Z. F. Liu and W. J. Jin, *New J. Chem.*, 2018, **42**, 10633–10641.

65 T. M. Shchegoleva, Z. A. Starikova, V. K. Trunov, O. B. Lantratova and I. E. Pokrovskaya, *Zhurnal Strukt. Khimii*, 1981, **22**, 93–94.

66 F. H. Herbstein and G. M. Reisner, *Acta Crystallogr. Sect. C*, 1984, **40**, 202–204.

67 J. C. Barnes, J. A. Chudek, R. Foster, F. Jarrett, F. Mackie, J. Paton and D. R. Twiselton, *Tetrahedron*, 1984, **40**, 1595–1601.

68 F. H. Herbstein and M. Kaftory, *Acta Crystallogr. Sect. B*, 1975, **31**, 68–75.

69 K. Prout and I. J. Tickle, *J. Chem. Soc. Perkin Trans. 2*, 1973, 1212–1215.

70 I. Ikemoto and H. Kuroda, *Acta Crystallogr. Sect. B*, 1968, **24**, 383–387.

71 J. Bernstein and H. Regev, *Cryst. Struct. Commun.*, 1980, **9**, 581.

72 C. C. Allen, J. C. A. Boeyens and D. C. Levendis, *South African J. Chem.*, 1989, **42**, 38.

73 C. K. Prout, I. J. Tickle and J. D. Wright, *J. Chem. Soc. Perkin Trans. 2*, 1973, 528–530.

74 Q. Zhang, *CSD Commun.*, CCDC 1845460: Experimental Crystal Structure Determination, 2018, DOI: 10.5517/ccdc.csd.cc1zybz7.

75 S. Bhattacharjee, B. Maiti and S. Bhattacharya, *Nanoscale*, 2016, **8**, 11224–11233.

76 Q. Zhang, *CSD Commun.*, Q. Zhang, *CCDC 1845458 Experimental Cryst. Struct. Determ. CSD Commun.* **2018**. https://doi.org/10.5517/ccdc.csd.cc1zybx5.

77 F. H. Herbstein and J. A. Snyman, *Philosphical Trans. R. Soc. London, Ser. A*, 1969, **264**, 635.

78 J.-S. Lee and S. C. Nyburg, *Acta Crystallogr. Sect. C*, 1985, **41**, 560–567.

79 D. Britton, *Acta Crystallogr. Sect. E*, 2005, **61**, o4188–o4189.

80 N. Asano, T. Harada, T. Sato, N. Tajima and R. Kuroda, *Chem. Commun.*, 2009, 899–901.

81 J. Bernstein, H. Regev, F. H. Herbstein, P. Main, S. H. Rizvi, K. Sasvari and B. Turcsanyi, *Proc. R. Soc. London, Ser. A*, 1975, **347**, 419.

82 L. McInnes, J. Healy, N. Saul and L. Großberger, *J. Open Source Softw.*, 2018, **3**, 861.

83 A. Mauri, V. Consonni, M. Pavan and R. Todeschini, *Match*, 2006, **56**, 237–248.

84 B. O'Neill, in *Elementary Differential Geometry (Second Edition)*, ed. B. O'Neill, Academic Press, Boston, Second Edi., 2006, pp. 3–42.

85 T. N. Hill and A. Lemmerer, *Acta Crystallogr. Sect. E*, 2018, **74**, 1772–1777.

86 H. Hoier, D. E. Zacharias, H. L. Carrell and J. P. Glusker, *Acta Crystallogr. Sect. C*, 1993, **49**, 523–526.

87 I. V Bulgarovskaya, V. E. Zavodnik and V. M. Vozzhennikov, *Acta Crystallogr. Sect. C*, 1987, **43**, 766–768.

88 D. L. Evans and W. T. Robinson, *Acta Crystallogr. Sect. B*, 1977, **33**, 2891–2893.

89 B. Averkiev, R. Isaac, E. V Jucov, V. N. Khrustalev, C. Kloc, L. E. McNeil and T. V Timofeeva, *Cryst. Growth Des.*, 2018, **18**, 4095–4102.

90 S. Yokokura, Y. Takahashi, H. Nonaka, H. Hasegawa, J. Harada, T. Inabe, R. Kumai, H. Okamoto, M. M. Matsushita and K. Awaga, *Chem. Mater.*, 2015, **27**, 4441–4449.
